# Supplementary material for: Cembranoid Diterpenes from South China Sea Soft Coral Sarcophyton crassocaule
Source: Mar Drugs. 2024 Nov 29;22(12):536. doi: 10.3390/md22120536 (PMC11676429; doi:10.3390/md22120536)
Supplement: Supplementary file 1 [file marinedrugs-22-00536-s001.zip › marinedrugs-3286358-supplementary.pdf]

Supporting Information

Cembranoid Diterpenes from South China Sea Soft Coral *Sarcophyton crassocaule*

Hanyang Peng [1,2,†], Yanbo Zeng [1,3,*,†], Rui Zhang [2], Li Yang [1], Fei Wu [1], Cuijuan Gai [1], Jingzhe Yuan [1], Wenjun Chang [1], Haofu Dai [1,*] and Xiachang Wang [2,*]

[1] Hainan Provincial Key Laboratory for Functional Components Research and Utilization of Marine Bio-Resources & National Key Laboratory for Tropical Crop Breeding, Institute of Tropical Bioscience and Biotechnology, Chinese Academy of Tropical Agricultural Sciences, Haikou 571101, China

[2] Jiangsu Key Laboratory for Functional Substances of Chinese Medicine, Nanjing University of Chinese Medicine, Nanjing 210023, China

[3] Zhanjiang Experimental Station of Chinese Academy of Tropical Agricultural Sciences, Zhanjiang 524013, China

* Correspondence:
zengyanbo@itbb.org.cn (Y.Z.); daihaofu@itbb.org.cn (H.D.); xiachangwang@njucm.edu.cn (X.W.)

† These authors contributed equally to this work.

List of Supporting Information

**Figure S1.**	^{1}H NMR spectrum (500 MHz, $CDCl_3$) of compound **1**
**Figure S2.**	^{13}C NMR spectrum (125 MHz, $CDCl_3$) of compound **1**
**Figure S3.**	The HSQC spectrum of compound **1** in $CDCl_3$
**Figure S4.**	The ^{1}H-^{1}H COSY spectrum of compound **1** in $CDCl_3$
**Figure S5.**	The HMBC spectrum of compound **1** in $CDCl_3$
**Figure S6.**	The ROESY spectrum of compound **1** in $CDCl_3$
**Figure S7.**	The HRESIMS spectrum of compound **1**
**Figure S8.**	The IR spectrum of compound **1**
**Figure S9.**	The UV spectrum of compound **1**
**Figure S10.**	^{1}H NMR spectrum (500 MHz, $CDCl_3$) of compound **2**
**Figure S11.**	^{13}C NMR spectrum (125 MHz, $CDCl_3$) of compound **2**
**Figure S12.**	DEPT spectrum (125 MHz, $CDCl_3$) of compound **2**
**Figure S13.**	The HSQC spectrum of compound **2** in $CDCl_3$
**Figure S14.**	The ^{1}H-^{1}H COSY spectrum of compound **2** in $CDCl_3$
**Figure S15.**	The HMBC spectrum of compound **2** in $CDCl_3$
**Figure S16.**	The ROESY spectrum of compound **2** in $CDCl_3$
**Figure S17.**	The HRESIMS spectrum of compound **2**
**Figure S18.**	The IR spectrum of compound **2**
**Figure S19.**	The UV spectrum of compound **2**
**Figure S20.**	^{1}H NMR spectrum (500 MHz, $CDCl_3$) of compound **3**
**Figure S21.**	^{13}C NMR spectrum (125 MHz, $CDCl_3$) of compound **3**
**Figure S22.**	DEPT spectrum (125 MHz, $CDCl_3$) of compound **3**
**Figure S23.**	The HSQC spectrum of compound **3** in $CDCl_3$
**Figure S24.**	The ^{1}H-^{1}H COSY spectrum of compound **3** in $CDCl_3$
**Figure S25.**	The HMBC spectrum of compound **3** in $CDCl_3$
**Figure S26.**	The ROESY spectrum of compound **3** in $CDCl_3$
**Figure S27.**	The HRESIMS spectrum of compound **3**
**Figure S28.**	The IR spectrum of compound **3**
**Figure S29.**	The UV spectrum of compound **3**
**Figure S30.**	^{1}H NMR spectrum (500 MHz, $CDCl_3$) of compound **4**
**Figure S31.**	^{13}C NMR spectrum (125 MHz, $CDCl_3$) of compound **4**
**Figure S32.**	DEPT spectrum (125 MHz, $CDCl_3$) of compound **4**
**Figure S33.**	The HSQC spectrum of compound **4** in $CDCl_3$
**Figure S34.**	The ^{1}H-^{1}H COSY spectrum of compound **4** in $CDCl_3$
**Figure S35.**	The HMBC spectrum of compound **4** in $CDCl_3$
**Figure S36.**	The ROESY spectrum of compound **4** in $CDCl_3$
**Figure S37.**	The HRESIMS spectrum of compound **4**
**Figure S38.**	The IR spectrum of compound **4**
**Figure S39.**	The UV spectrum of compound **4**
**Figure S40.**	^{1}H NMR spectrum (500 MHz, $CDCl_3$) of compound **5**
**Figure S41.**	^{13}C NMR spectrum (125 MHz, $CDCl_3$) of compound **5**

**Figure S42.**	The HSQC spectrum of compound **5** in $CDCl_3$
**Figure S43.**	The ^1H-^1H COSY spectrum of compound **5** in $CDCl_3$
**Figure S44.**	The HMBC spectrum of compound **5** in $CDCl_3$
**Figure S45.**	The ROESY spectrum of compound **5** in $CDCl_3$
**Figure S46.**	The HRESIMS spectrum of compound **5**
**Figure S47.**	The IR spectrum of compound **5**
**Figure S48.**	The UV spectrum of compound **5**
**Figure S49.**	^1H NMR spectrum (500 MHz, $CDCl_3$) of compound **6**
**Figure S50.**	^{13}C NMR spectrum (125 MHz, $CDCl_3$) of compound **6**
**Figure S51.**	^1H NMR spectrum (500 MHz, $CDCl_3$) of compound **7**
**Figure S52.**	^{13}C NMR spectrum (125 MHz, $CDCl_3$) of compound **7**
**Figure S53.**	^1H NMR spectrum (500 MHz, $CDCl_3$) of compound **8**
**Figure S54.**	^{13}C NMR spectrum (125 MHz, $CDCl_3$) of compound **8**
**Figure S55.**	^1H NMR spectrum (500 MHz, $CDCl_3$) of compound **9**
**Figure S56.**	^{13}C NMR spectrum (125 MHz, $CDCl_3$) of compound **9**
**Figure S57.**	^1H NMR spectrum (500 MHz, $CDCl_3$) of compound **10**
**Figure S58.**	^{13}C NMR spectrum (125 MHz, $CDCl_3$) of compound **10**
**Table S1.**	Crystal data and structure refinement for compound **1**
**Table S2.**	Conformational analysis of the optimized isomers of **2**
**Table S3.**	The coordinates of the optimized conformers of **2**
**Table S4.**	Experimental and calculated ^{13}C NMR chemical shifts of **3**
**Table S5.**	Experimental and calculated ^1H NMR chemical shifts of **3**
**Table S6.**	Conformational analysis of the optimized isomers of **3**
**Table S7.**	The coordinates of the optimized conformers of 1*R*,4*S*,11*S*,12*R*-**3**
**Table S8.**	The coordinates of the optimized conformers of 1*R*,4*S*,11*R*,12*S*-**3**.
**Table S9.**	Conformational analysis of the optimized isomers of **4**
**Table S10.**	The coordinates of the optimized conformers of **4**
**Table S11.**	Conformational analysis of the optimized isomers of **5**
**Table S12.**	The coordinates of the optimized conformers of **5**

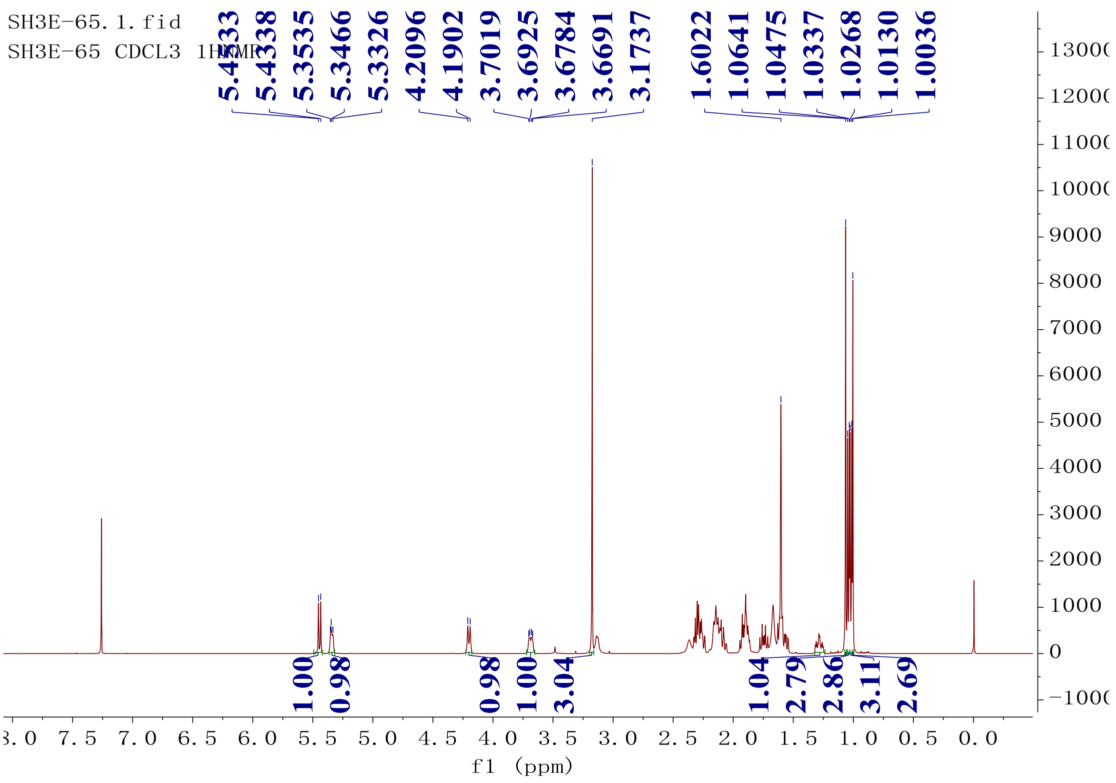

Figure S1. ^{1}H NMR spectrum (500 MHz, $CDCl_3$) of compound **1**

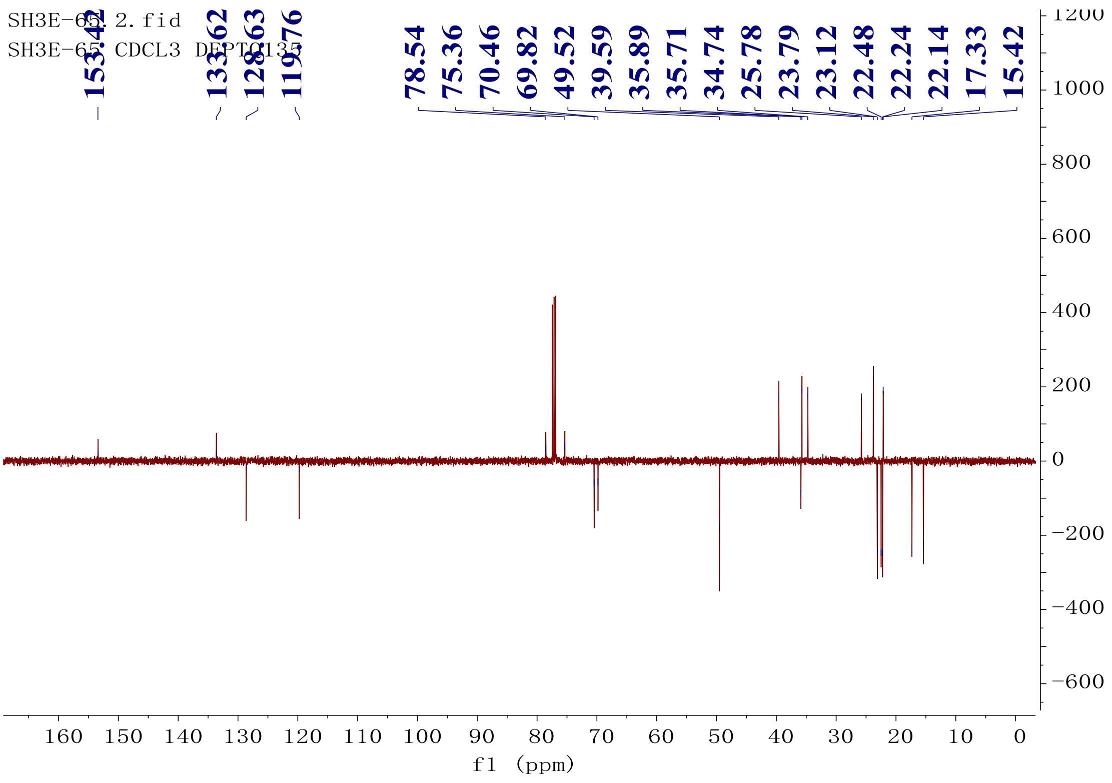

Figure S2. ^{13}C NMR spectrum (125 MHz, $CDCl_3$) of compound **1**

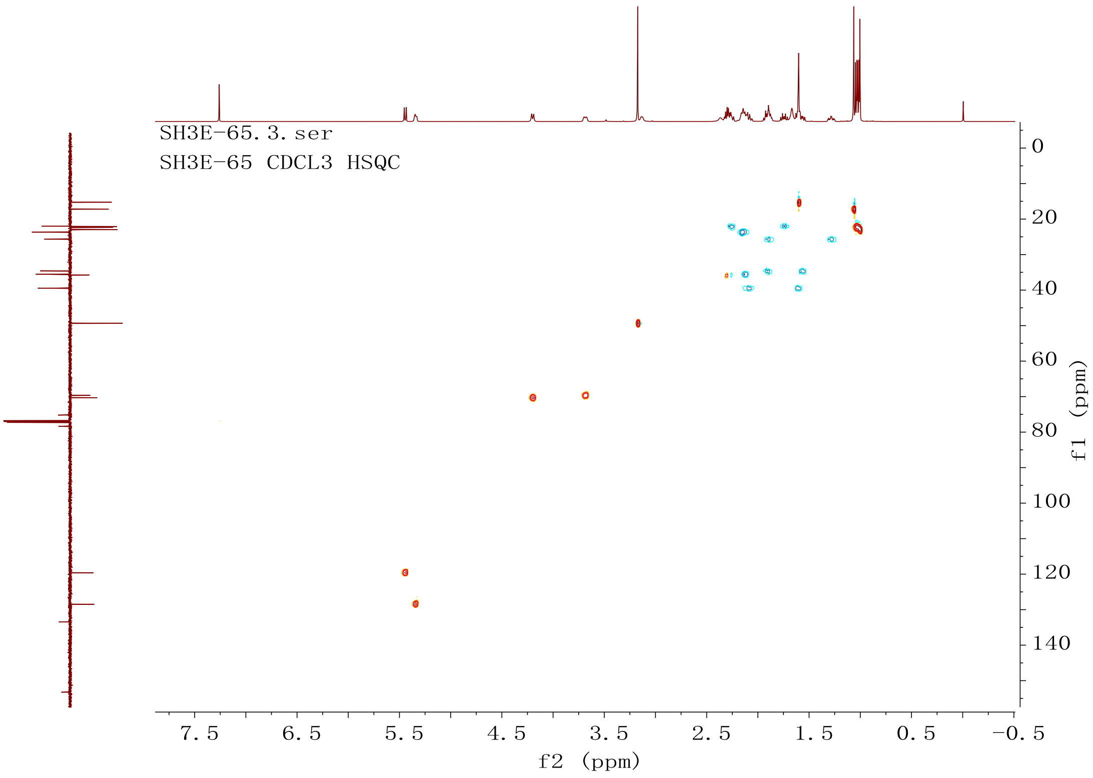

Figure S3. The HSQC spectrum of compound **1** in $CDCl_3$

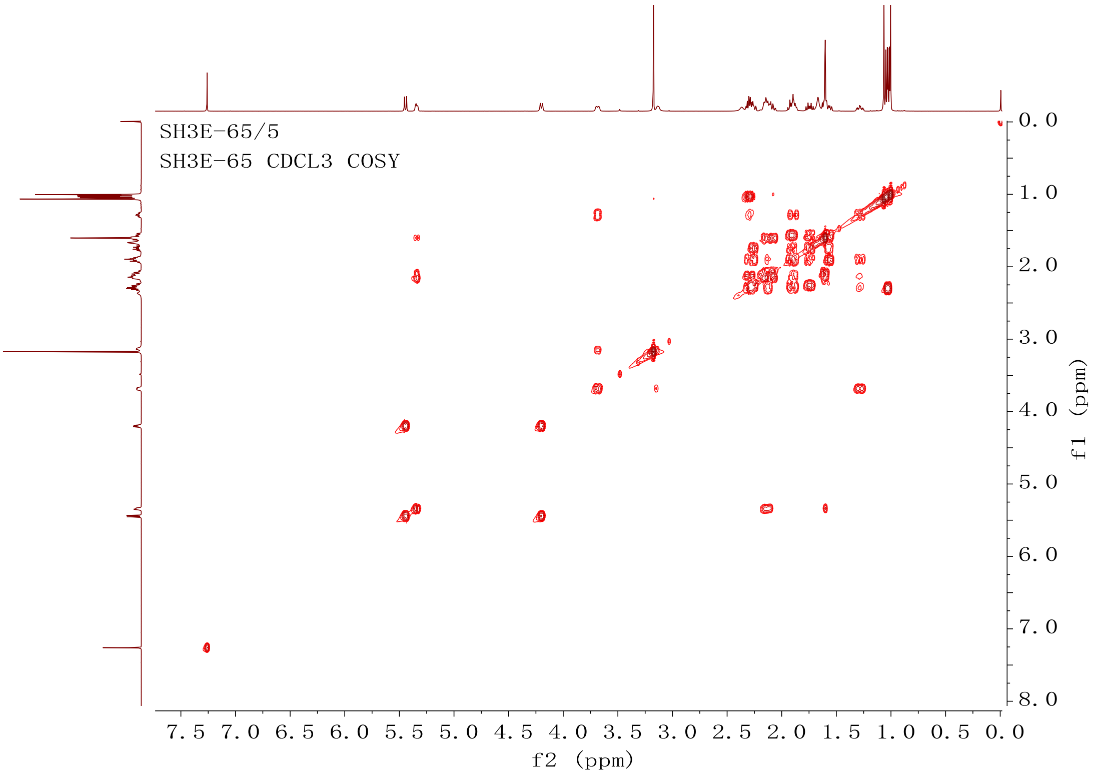

Figure S4. The ^1H-^1H COSY spectrum of compound **1** in $CDCl_3$

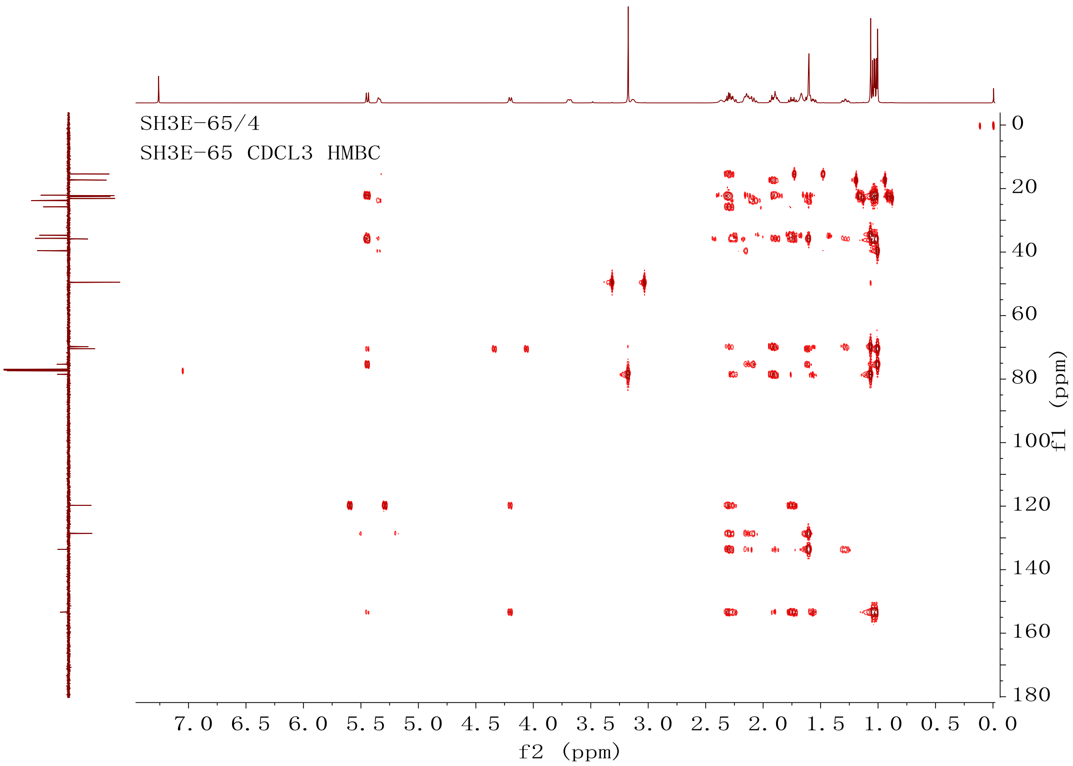

Figure S5. The HMBC spectrum of compound **1** in $CDCl_3$

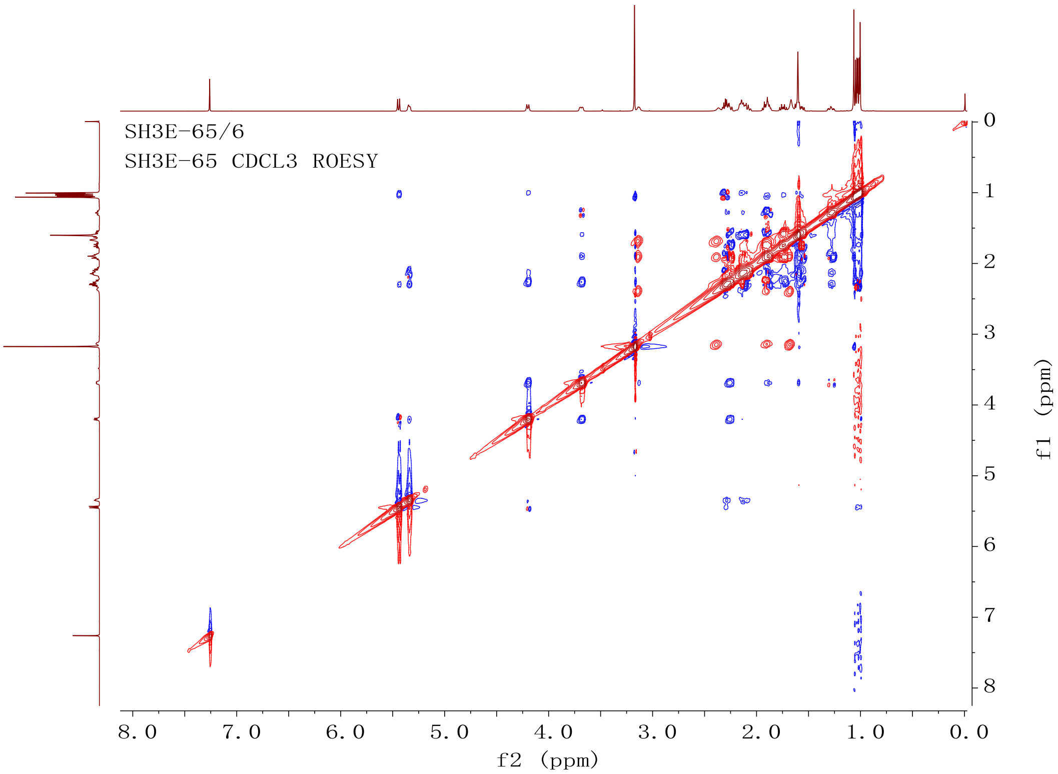

Figure S6. The ROESY spectrum of compound **1** in $CDCl_3$

Mass Spectrum SmartFormula Report

Analysis Info

Analysis Name	D:\Data\A501\WYL\2022\20221017\B502-SH3E-65_BE2_01_5118.d	Acquisition Date	2022-10-17 17:47:55	
Method	lc-ms_as_ms-0.5MIN.m	Operator	Demo User	
Sample Name	B502-SH3E-65	Instrument	compact	8255754.20156
Comment				

Acquisition Parameter

Source Type	ESI	Ion Polarity	Positive	Set Nebulizer	1.8 Bar
Focus	Not active	Set Capillary	4500 V	Set Dry Heater	220 °C
Scan Begin	50 m/z	Set End Plate Offset	-500 V	Set Dry Gas	4.0 l/min
Scan End	2500 m/z	Set Charging Voltage	2000 V	Set Divert Valve	Waste
		Set Corona	0 nA	Set APCI Heater	0 °C

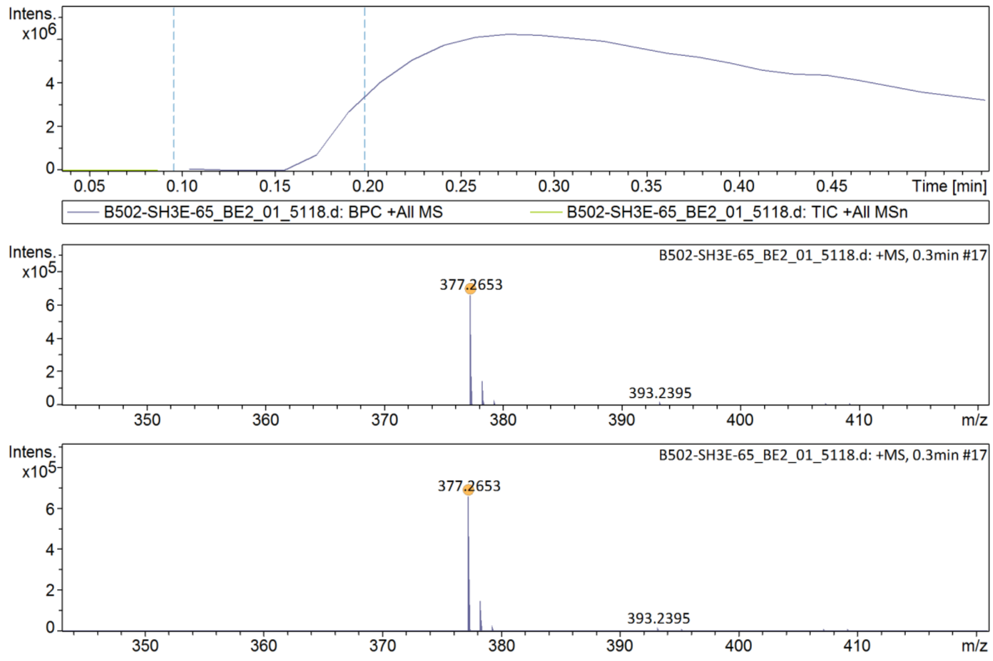

Meas. m/z	#	Ion Formula	m/z	err [ppm]	mSigma	# mSigma	Score	rdb	e⁻ Conf	N-Rule	Adduct
377.2653	1	C21H38NaO4	377.2662	2.4	4.9	1	100.00	3.0	even	ok	M+Na

Figure S7. The HRESIMS spectrum of compound **1**

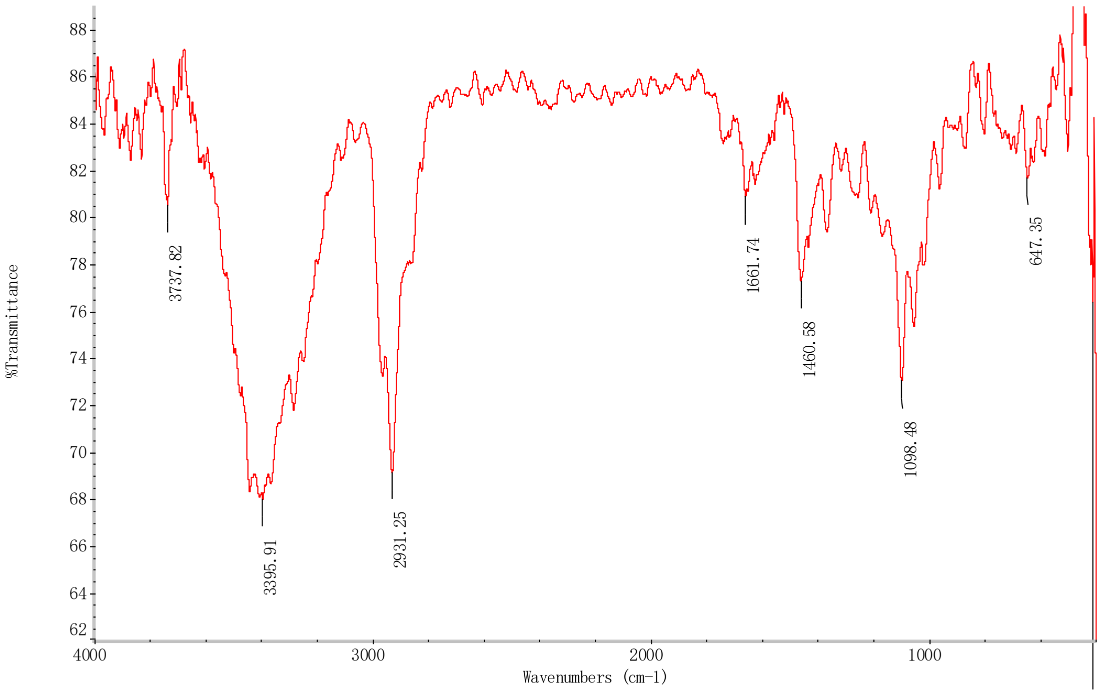

Figure S8. The IR spectrum of compound **1**

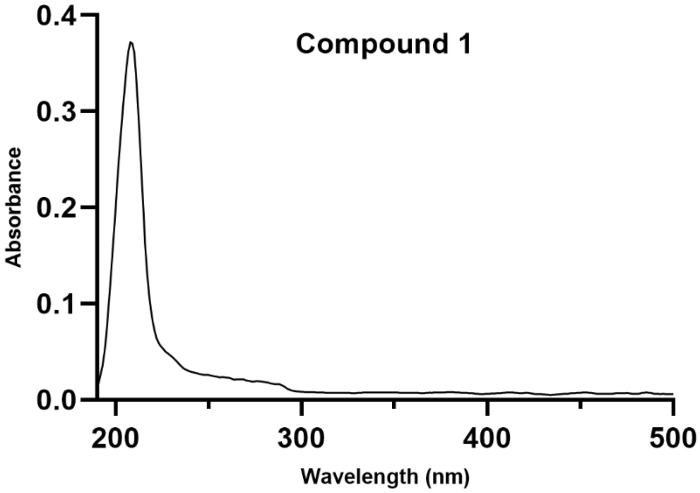

Figure S9. The UV spectrum of compound **1**

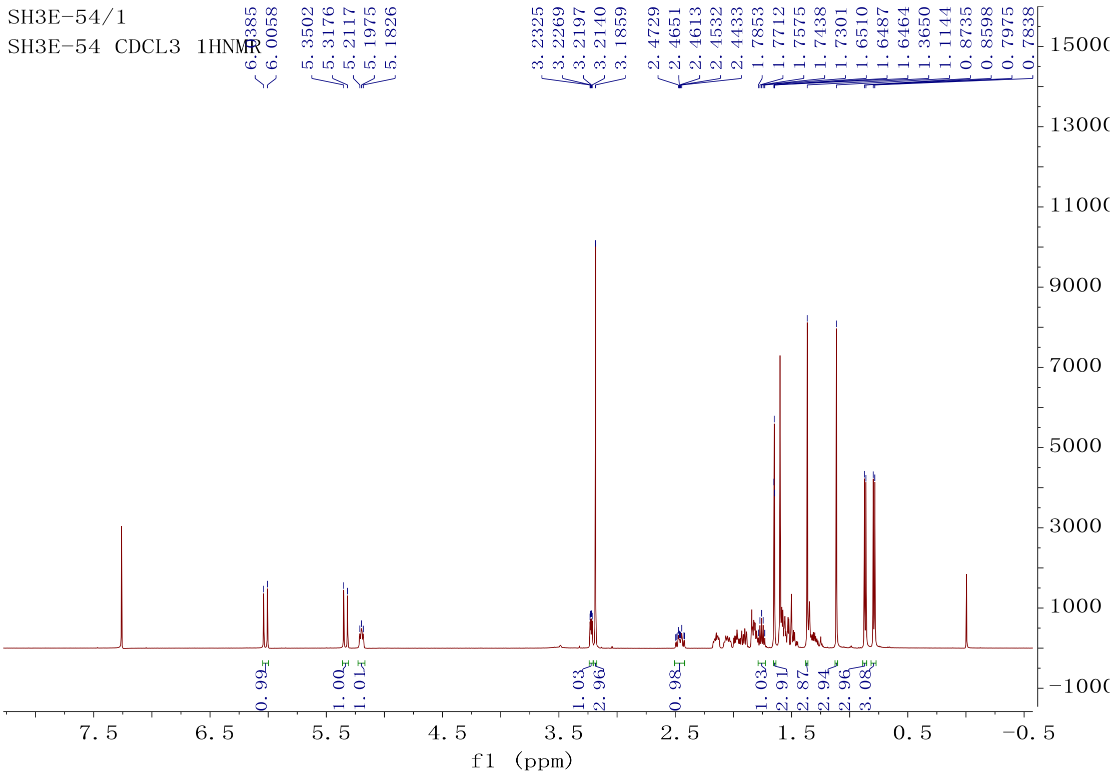

Figure S10. ^{1}H NMR spectrum (500 MHz, $CDCl_3$) of compound **2**

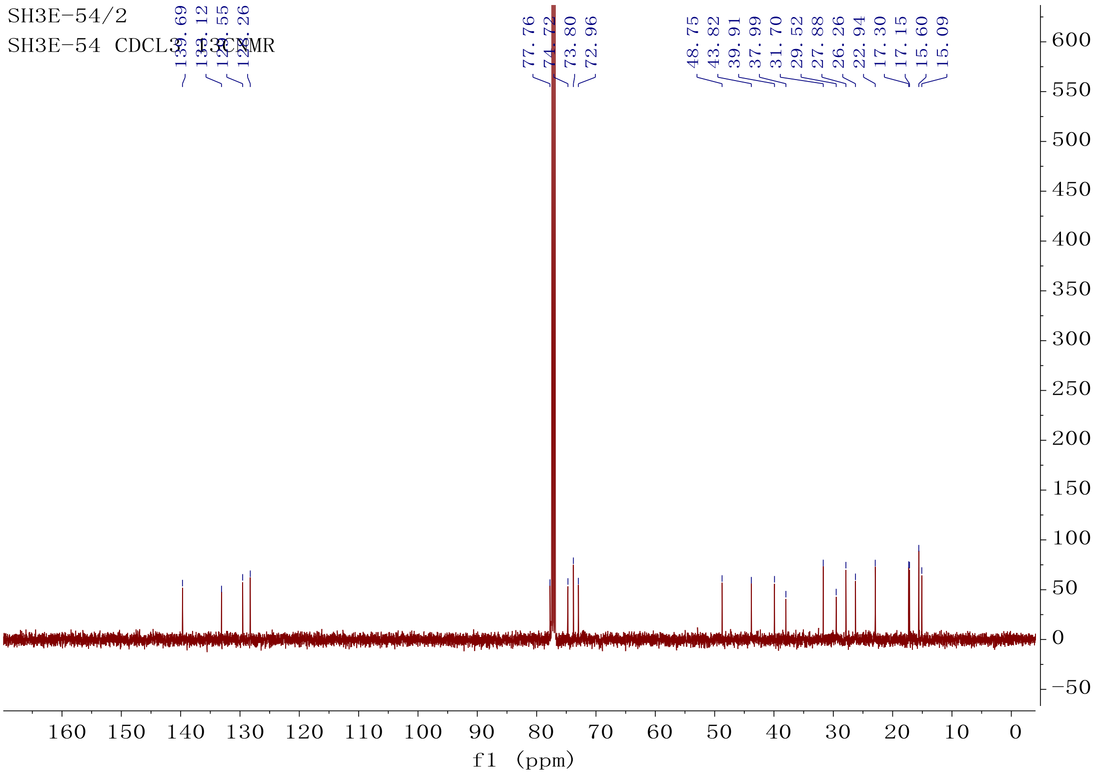

Figure S11. ^{13}C NMR spectrum (125 MHz, $CDCl_3$) of compound **2**

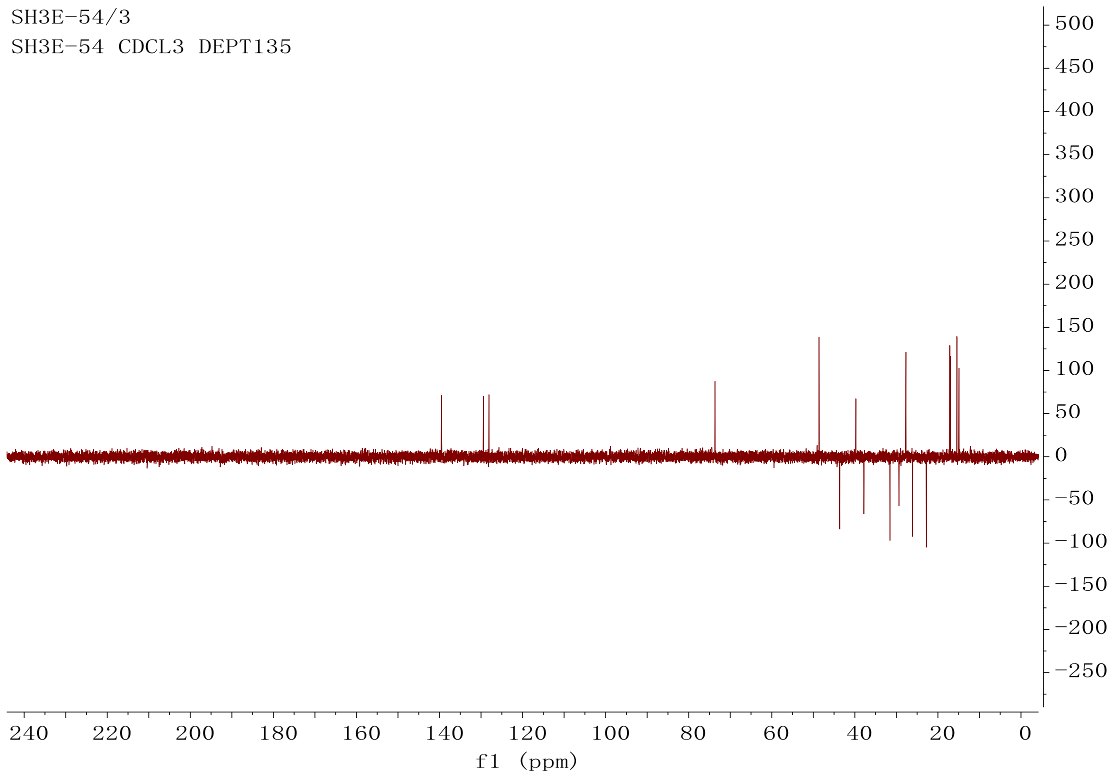

Figure S12. DEPT spectrum (125 MHz, $CDCl_3$) of compound **2**

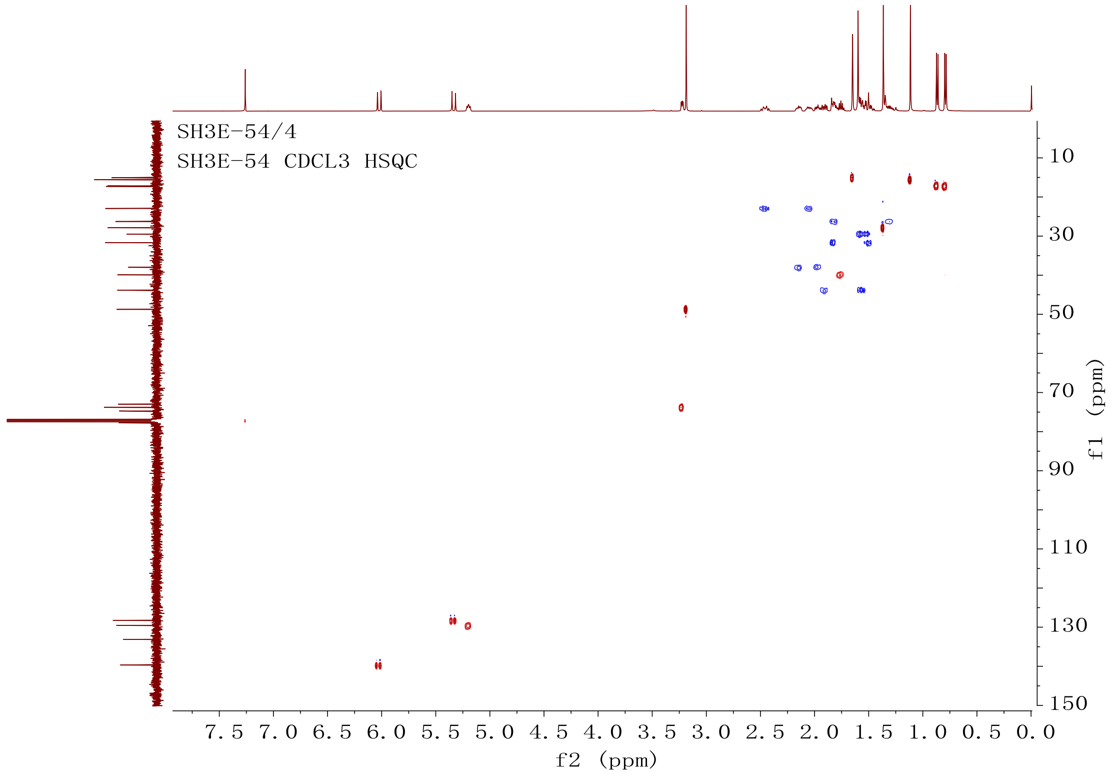

Figure S13. The HSQC spectrum of compound **2** in $CDCl_3$

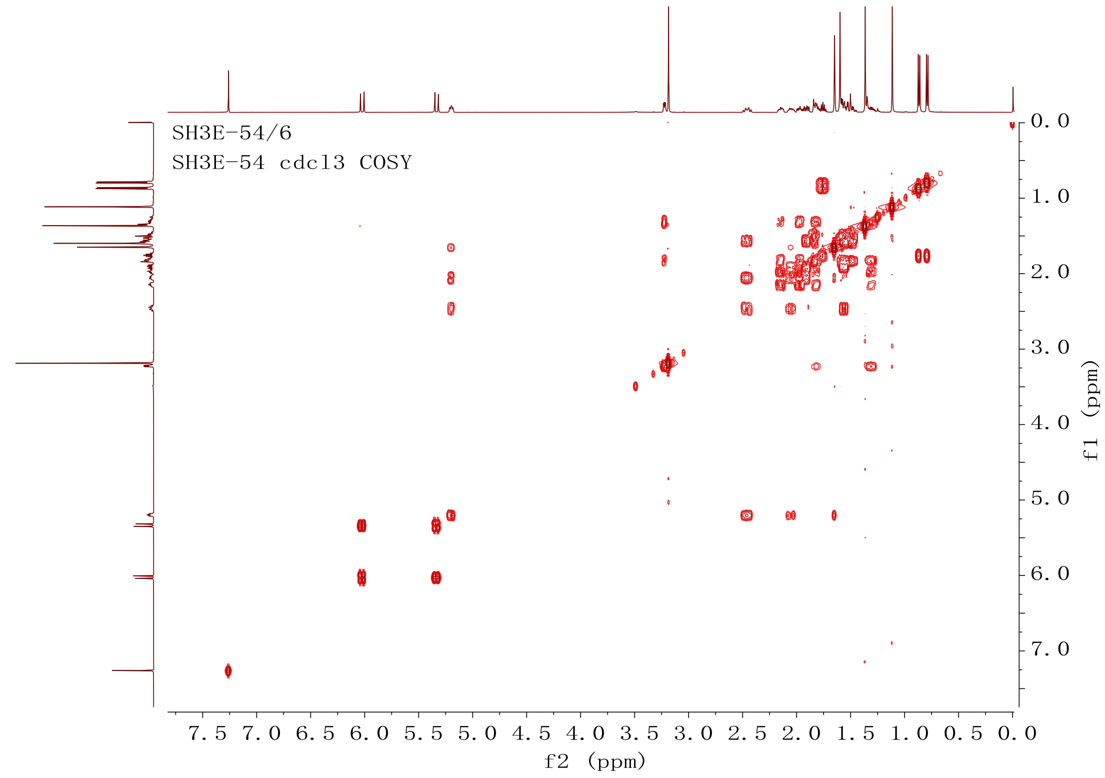

Figure S14. The ^{1}H-^{1}H COSY spectrum of compound **2** in $CDCl_3$

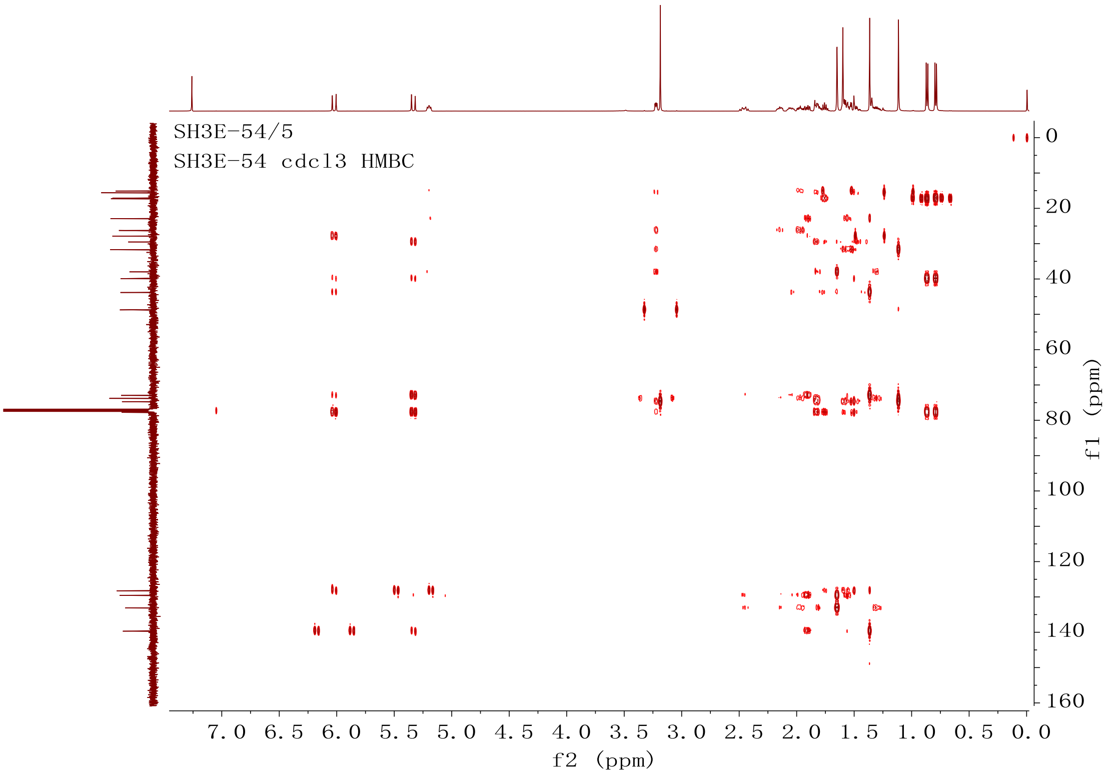

Figure S15. The HMBC spectrum of compound **2** in $CDCl_3$

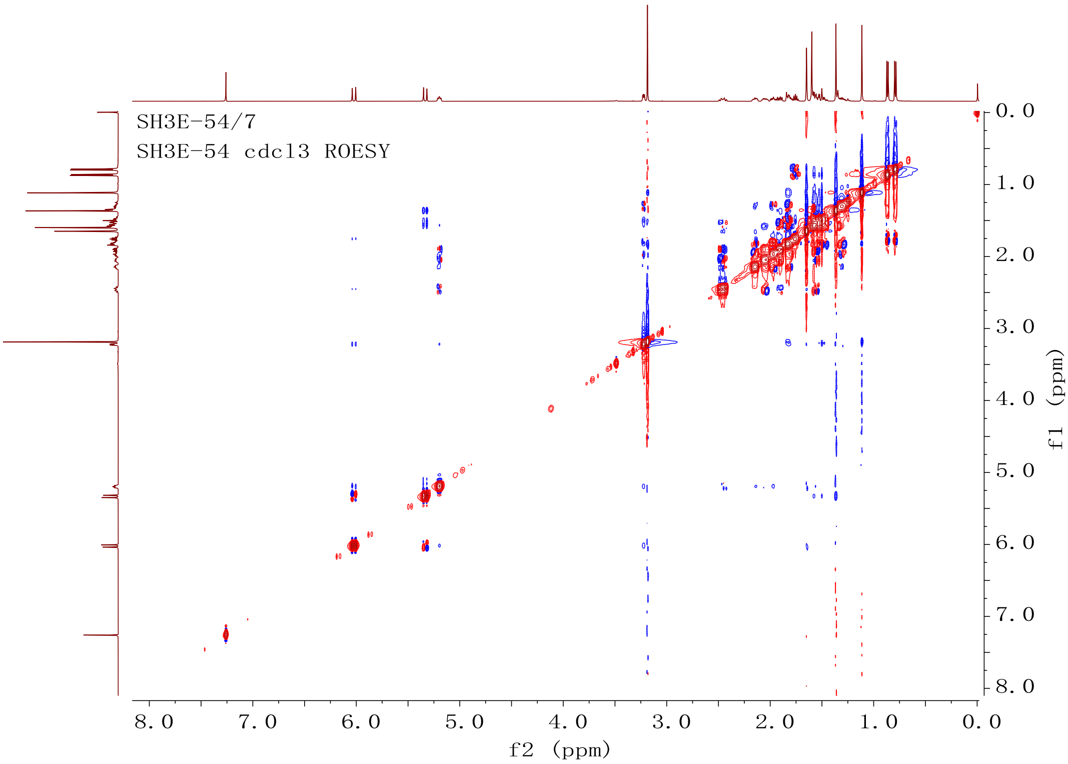

Figure S16. The ROESY spectrum of compound **2** in $CDCl_3$

Mass Spectrum SmartFormula Report

Analysis Info

		Acquisition Date	2022-11-22 16:41:30
Analysis Name	D:\Data\A501\WYL\2022\20221122\B502-SH3E-54_GA2_01_5681.d		
Method	lc-ms_as_ms-0.5MIN-20221111.m	Operator	Demo User
Sample Name	B502-SH3E-54	Instrument	compact 8255754.20156
Comment			

Acquisition Parameter

Source Type	ESI	Ion Polarity	Positive	Set Nebulizer	1.8 Bar
Focus	Not active	Set Capillary	3500 V	Set Dry Heater	220 °C
Scan Begin	50 m/z	Set End Plate Offset	-500 V	Set Dry Gas	4.0 l/min
Scan End	2500 m/z	Set Charging Voltage	2000 V	Set Divert Valve	Waste
		Set Corona	0 nA	Set APCI Heater	0 °C

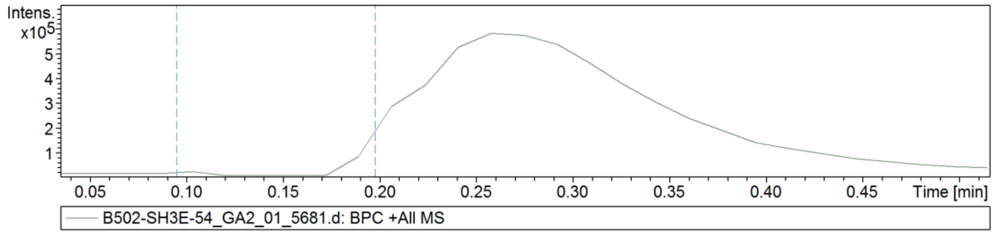

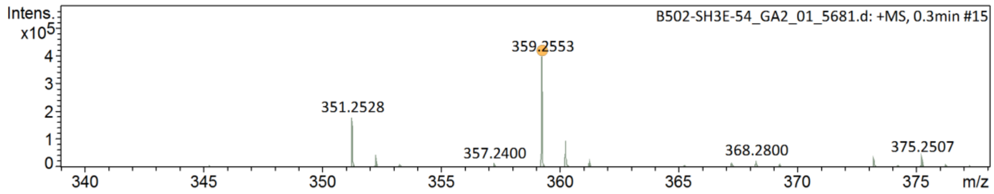

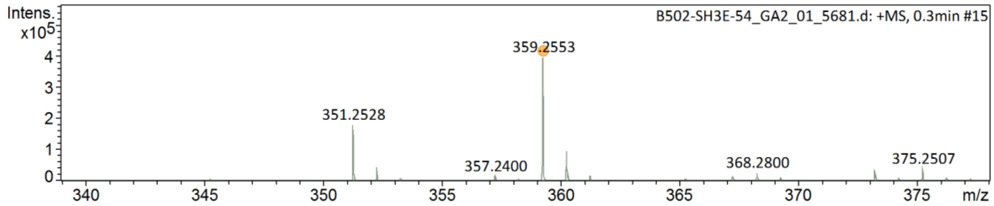

Meas. m/z	#	Ion Formula	m/z	err [ppm]	mSigma	# mSigma	Score	rdb	e⁻ Conf	N-Rule	Adduct
359.2553	1	C21H36NaO3	359.2557	1.0	5.0	1	100.00	4.0	even	ok	M+Na

Figure S17. The HRESIMS spectrum of compound **2**

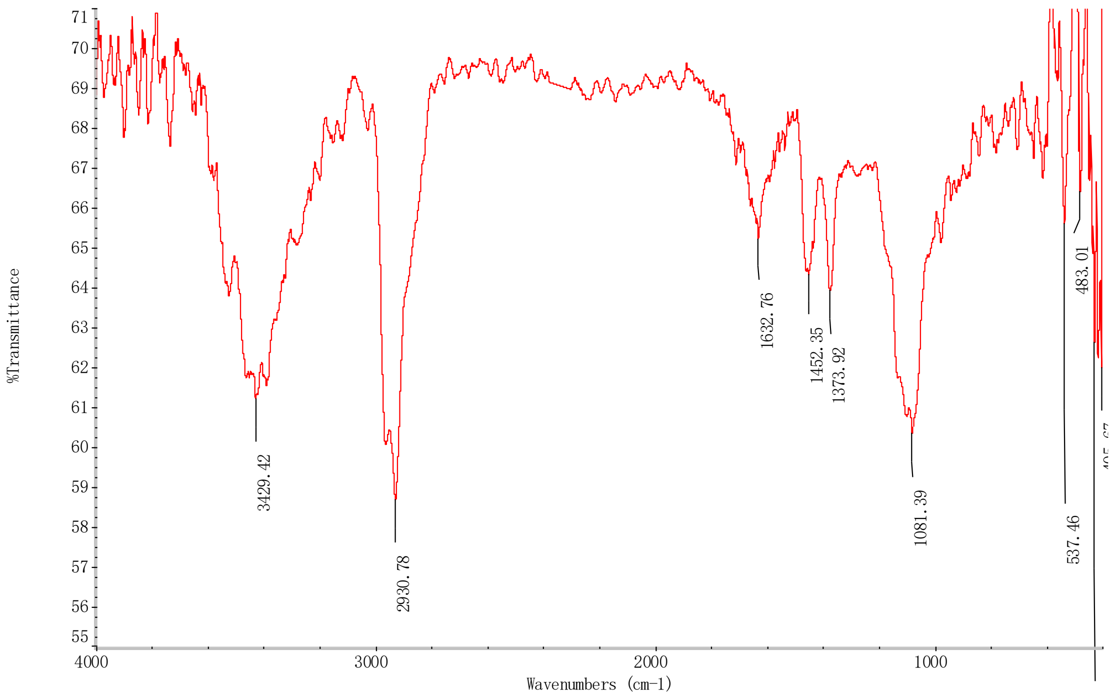

Figure S18. The IR spectrum of compound **2**

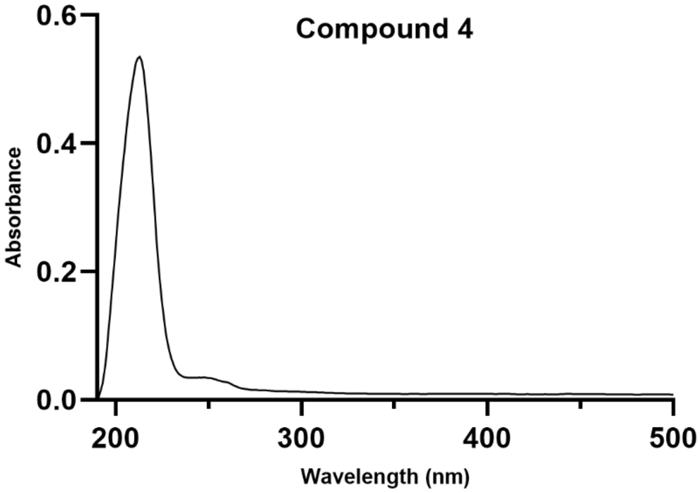

Figure S19. The UV spectrum of compound **2**

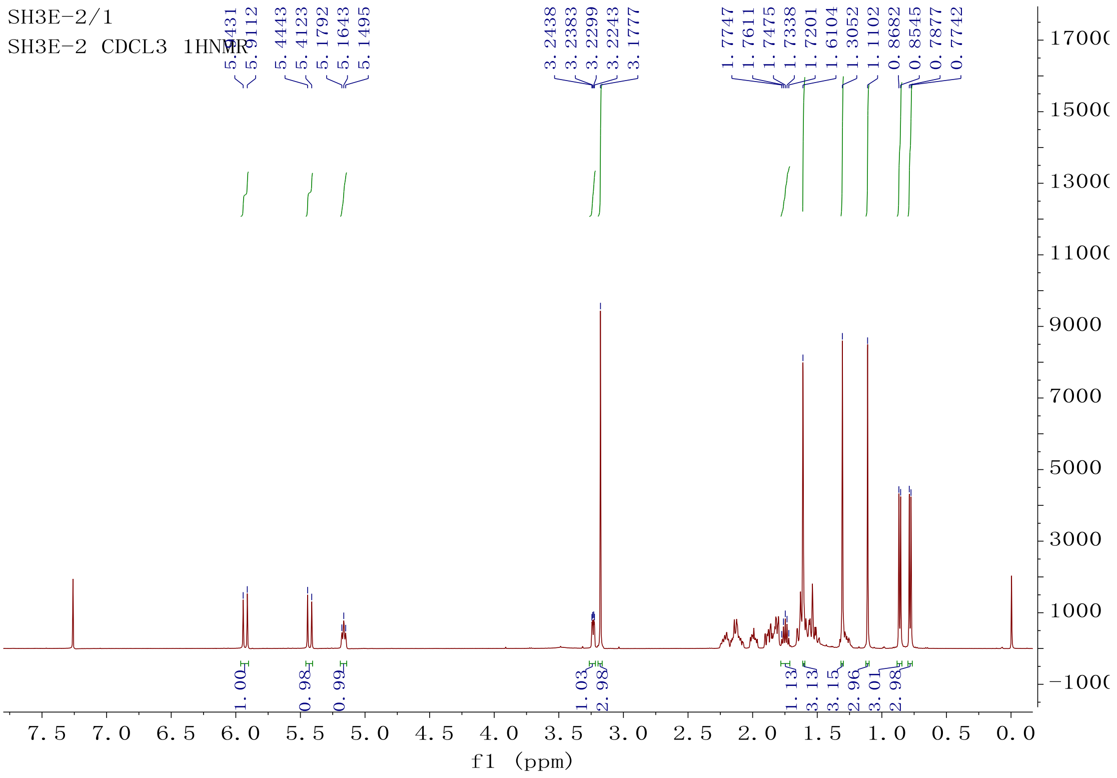

Figure S20. ^{1}H NMR spectrum (500 MHz, $CDCl_3$) of compound **3**

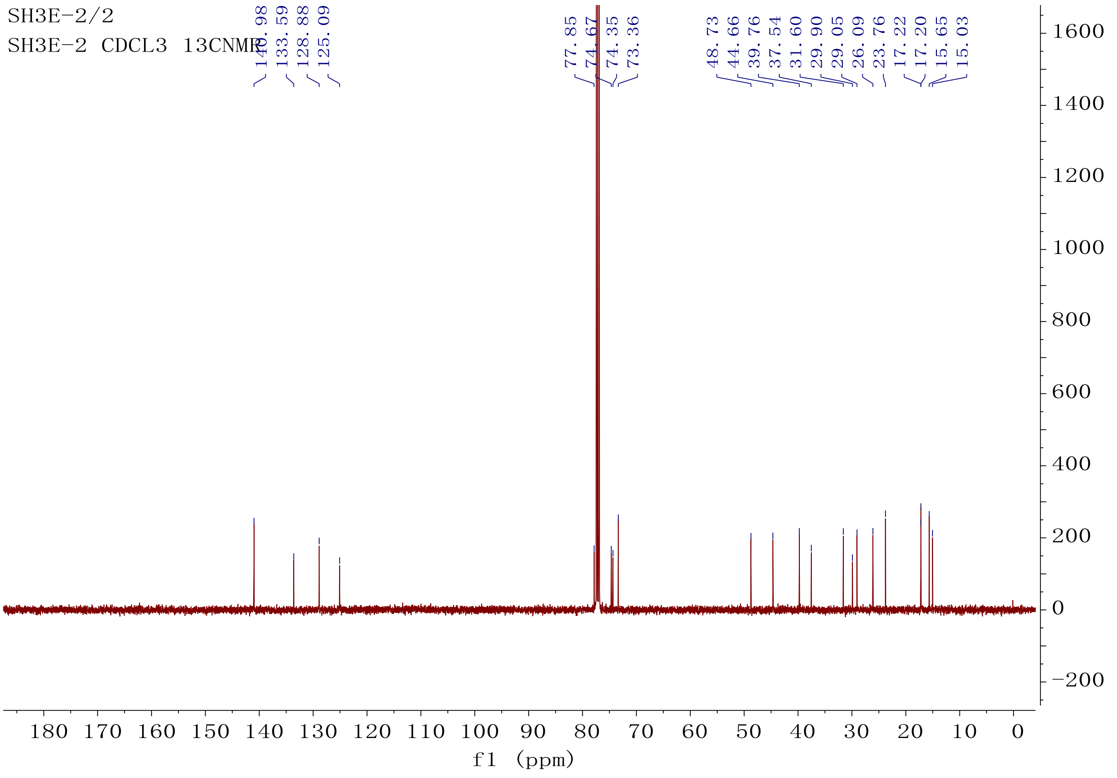

Figure S21. ^{13}C NMR spectrum (125 MHz, $CDCl_3$) of compound **3**

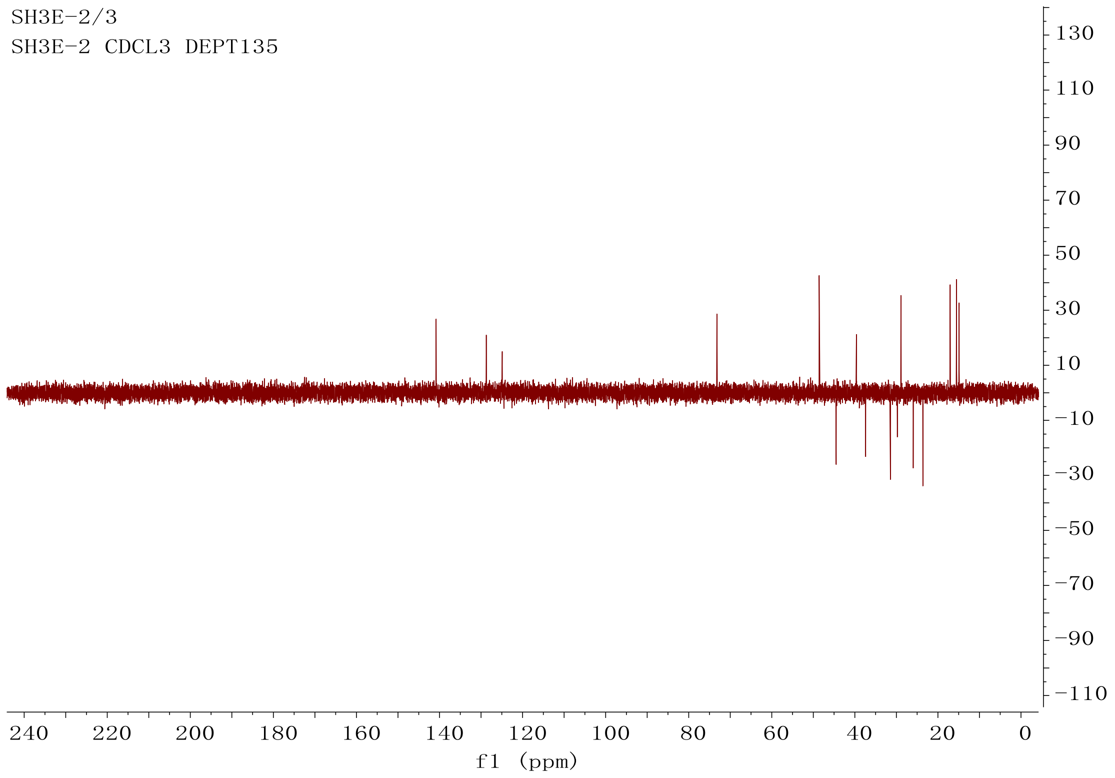

Figure S22. DEPT spectrum (125 MHz, $CDCl_3$) of compound **3**

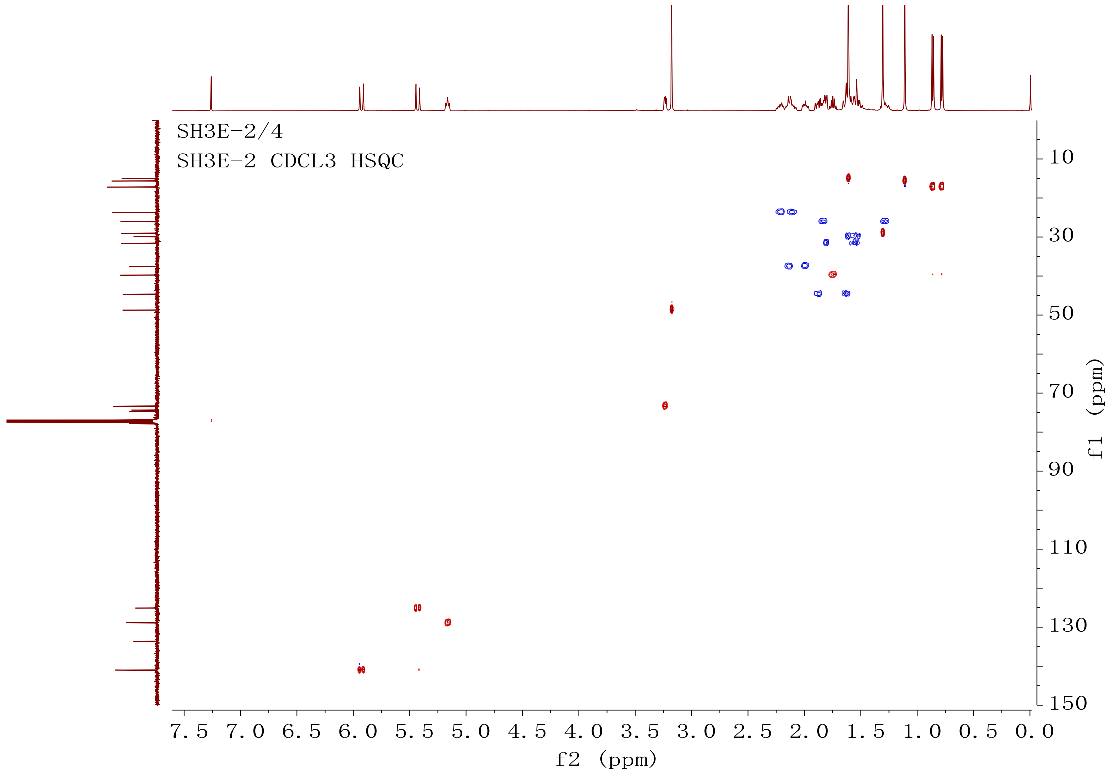

Figure S23. The HSQC spectrum of compound **3** in $CDCl_3$

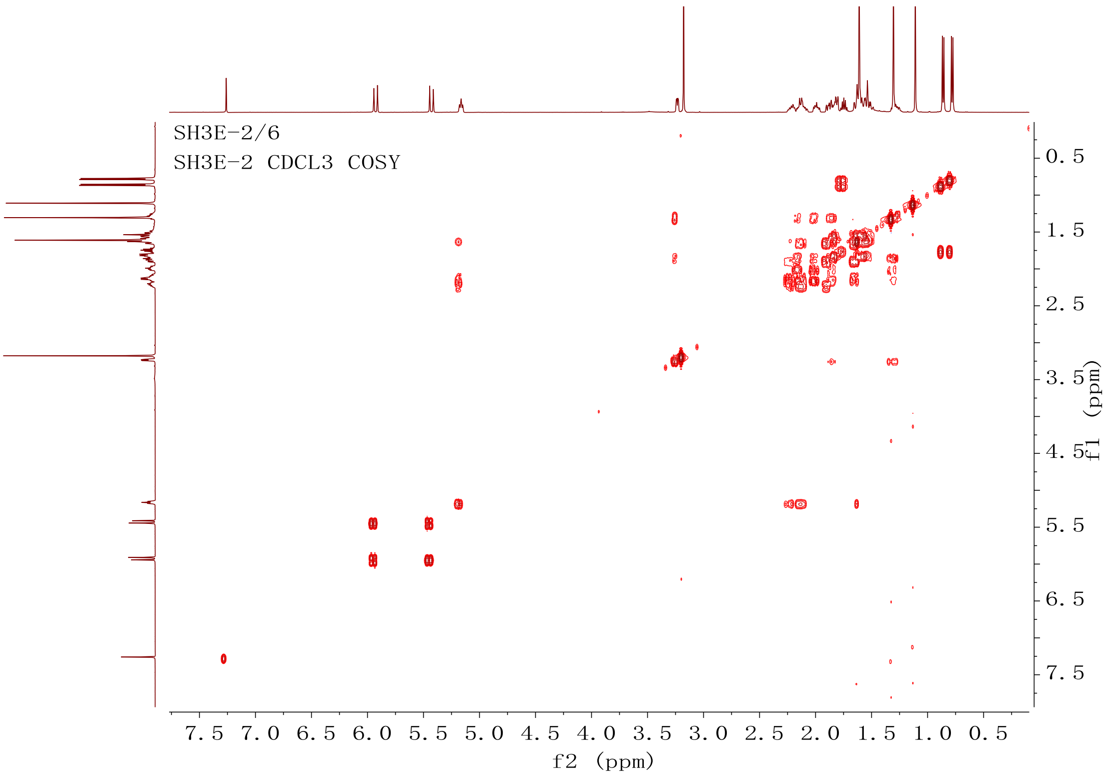

Figure S24. The ^{1}H-^{1}H COSY spectrum of compound **3** in $CDCl_3$

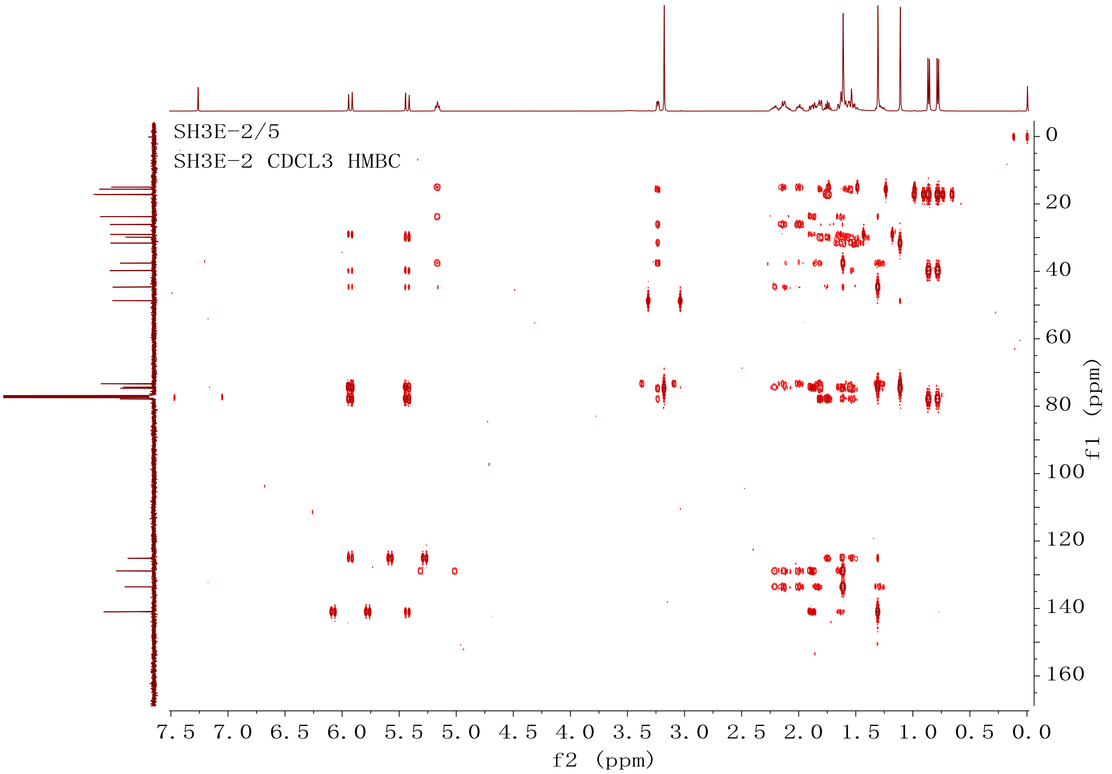

Figure S25. The HMBC spectrum of compound **3** in $CDCl_3$

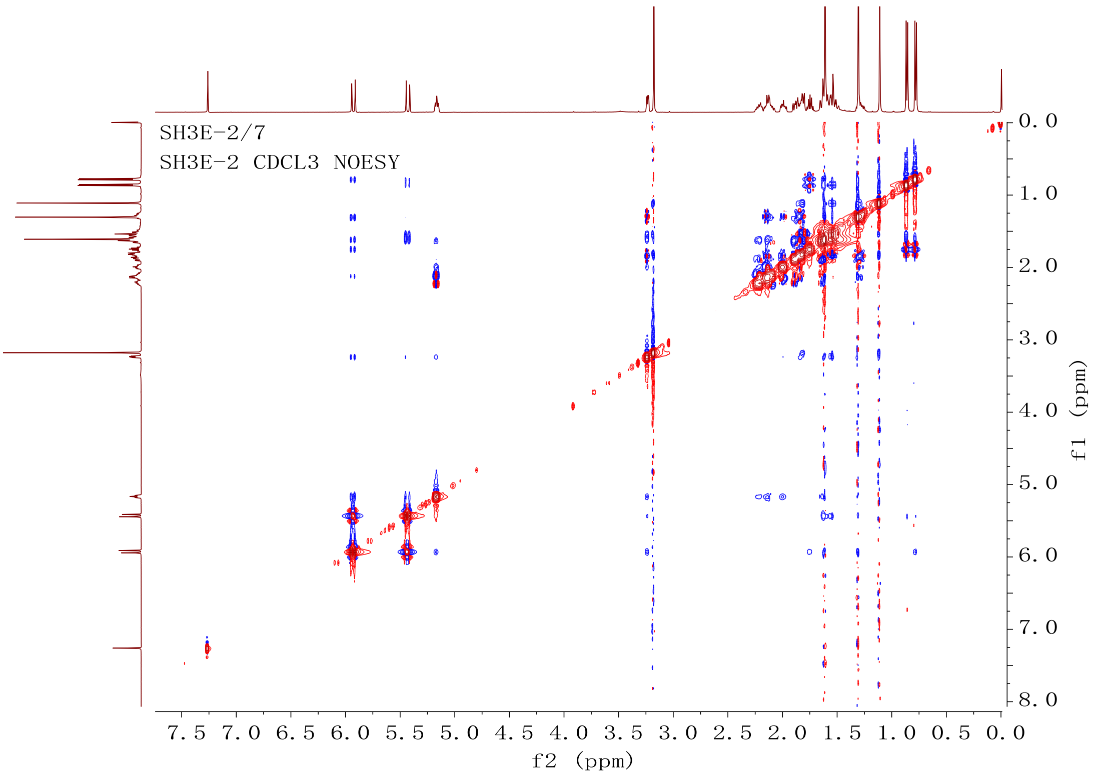

Figure S26. The ROESY spectrum of compound **3** in $CDCl_3$

Mass Spectrum SmartFormula Report

Analysis Info

Analysis Name	D:\Data\A501\WYL\2022\20220517\B502-SH3E-2-M_GB4_01_4299.d	Acquisition Date	2022-05-17 10:12:23
Method	lc-ms_as_ms-0.5MIN.m	Operator	Demo User
Sample Name	B502-SH3E-2-M	Instrument	compact 8255754.20156
Comment			

Acquisition Parameter

Source Type	ESI	Ion Polarity	Positive	Set Nebulizer	1.8 Bar
Focus	Not active	Set Capillary	3500 V	Set Dry Heater	220 °C
Scan Begin	50 m/z	Set End Plate Offset	-500 V	Set Dry Gas	4.0 l/min
Scan End	2500 m/z	Set Charging Voltage	2000 V	Set Divert Valve	Waste
		Set Corona	0 nA	Set APCI Heater	0 °C

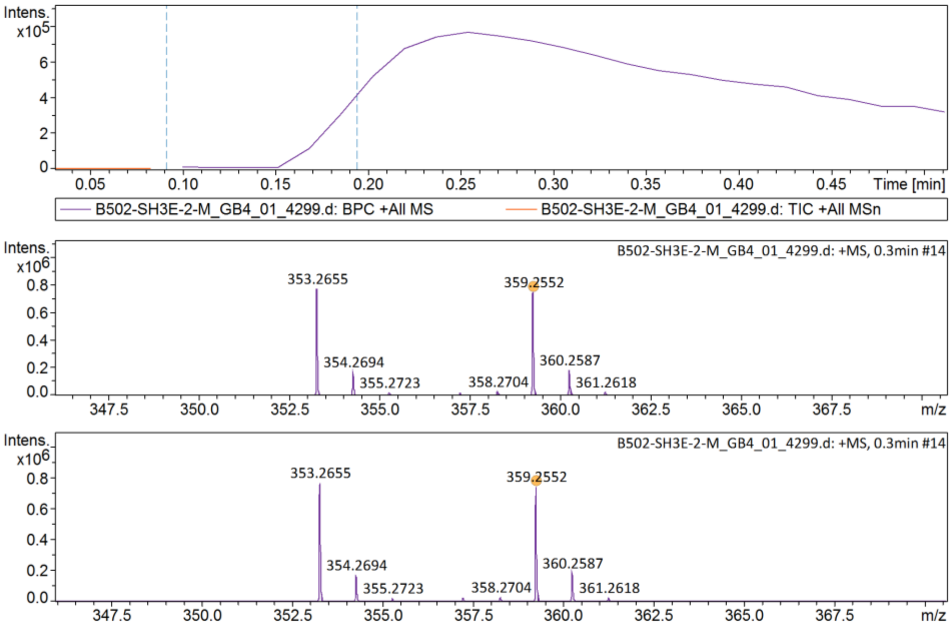

Meas. m/z	#	Ion Formula	m/z	err [ppm]	mSigma	# mSigma	Score	rdb	e⁻ Conf	N-Rule	Adduct
359.2552	1	C21H36NaO3	359.2557	1.4	6.0	1	100.00	4.0	even	ok	M+Na

Figure S27. The HRESIMS spectrum of compound **3**

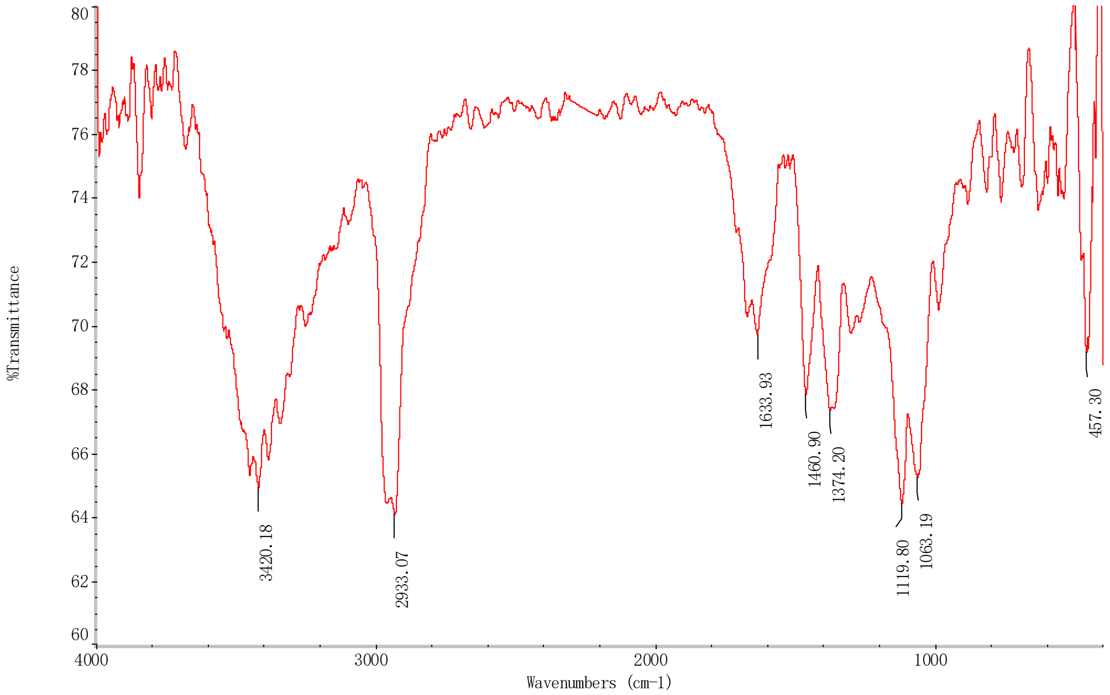

Figure S28. The IR spectrum of compound **3**

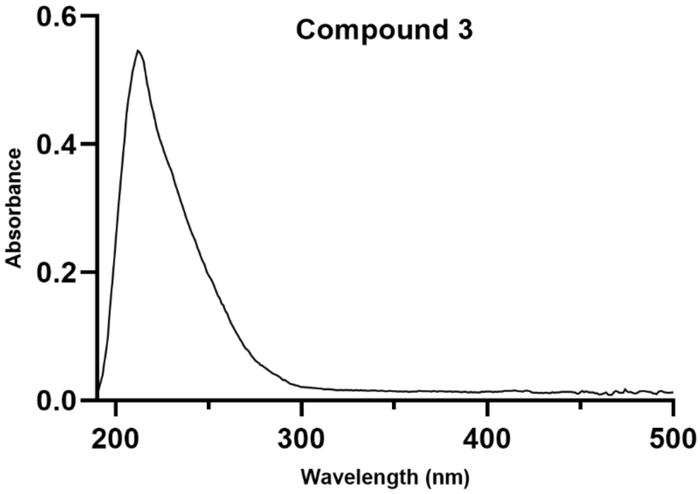

Figure S29. The UV spectrum of compound **3**

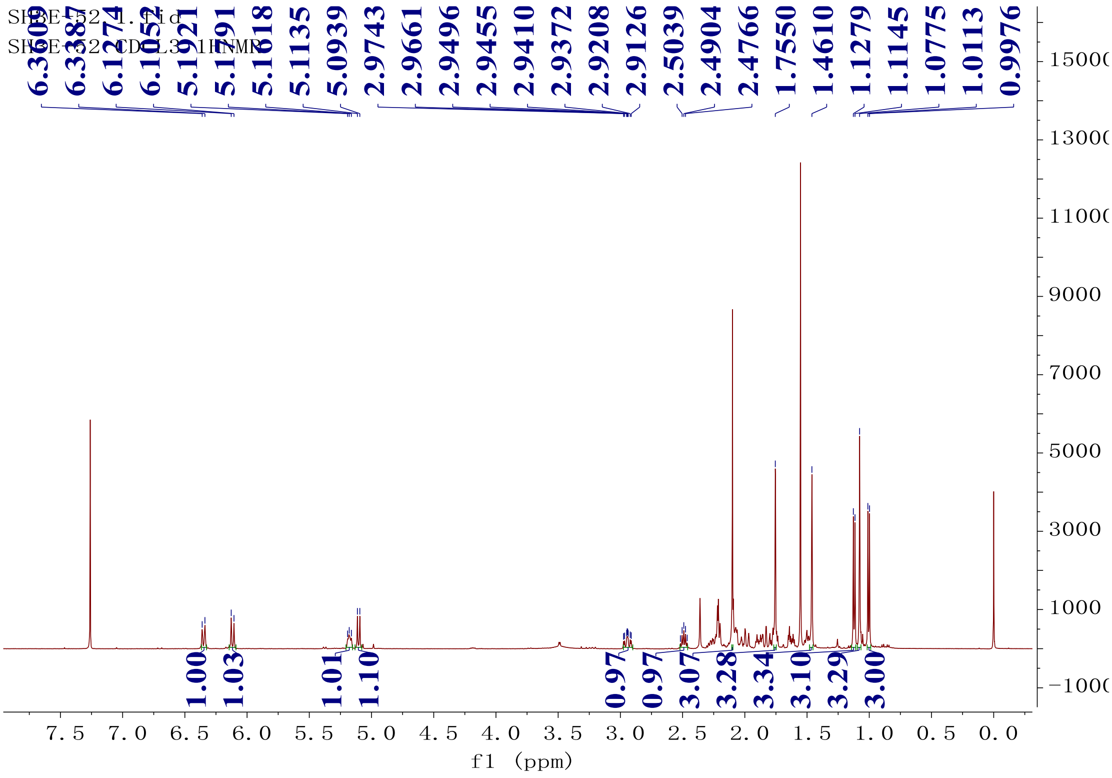

Figure S30. ^{1}H NMR spectrum (500 MHz, $CDCl_3$) of compound **4**

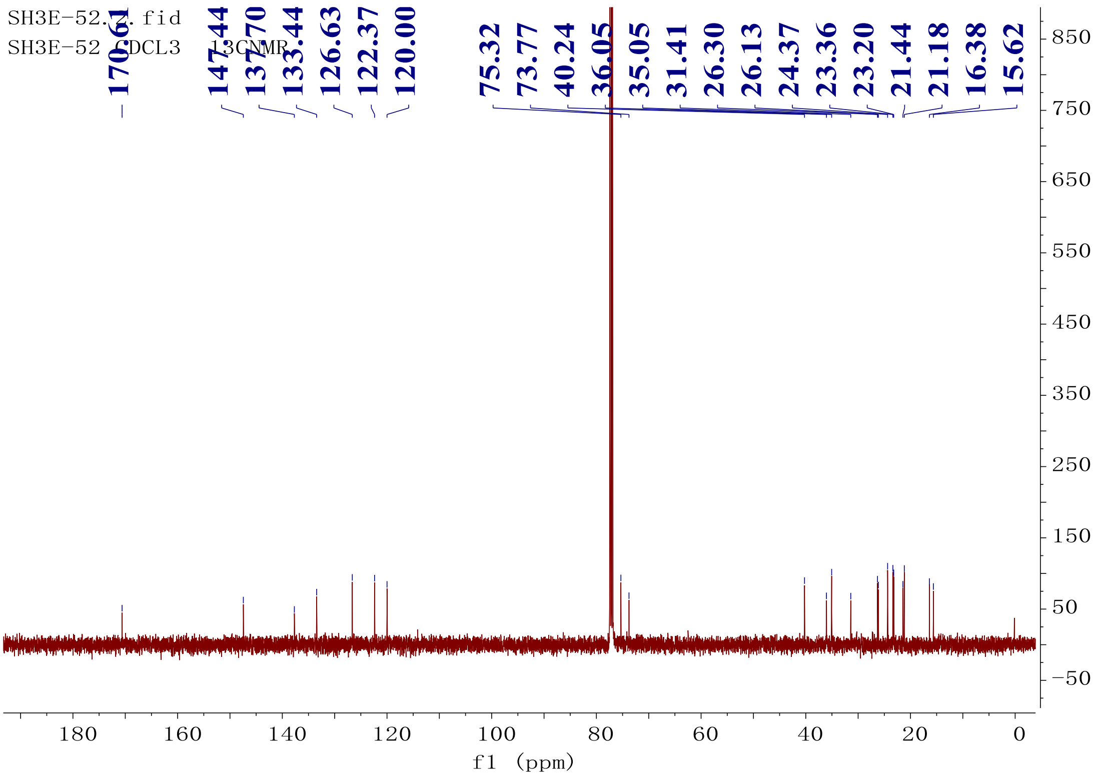

Figure S31. ^{13}C NMR spectrum (125 MHz, $CDCl_3$) of compound **4**

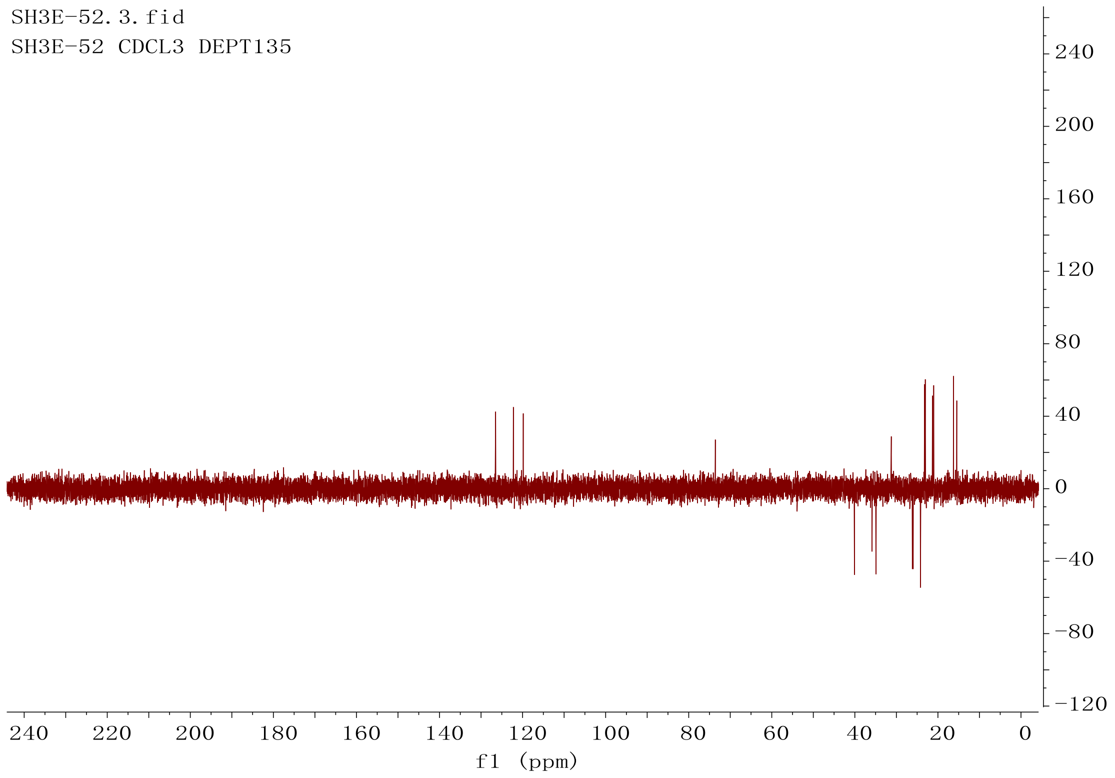

Figure S32. DEPT spectrum (125 MHz, $CDCl_3$) of compound **4**

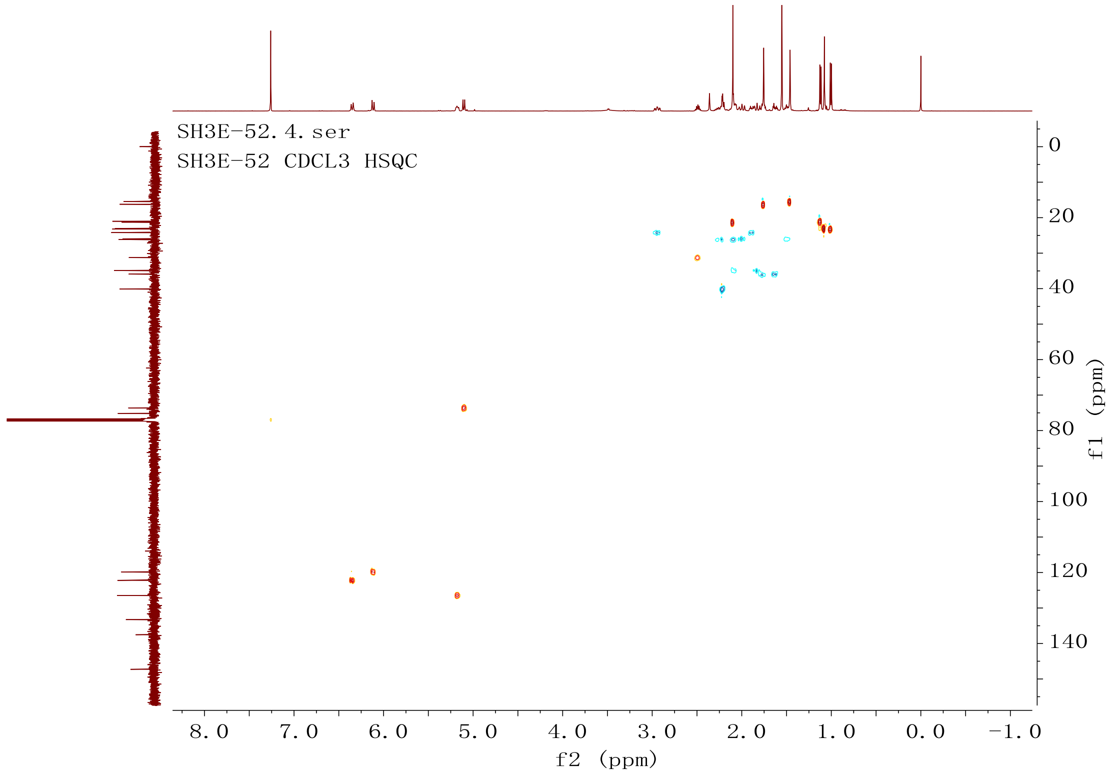

Figure S33. The HSQC spectrum of compound **4** in $CDCl_3$

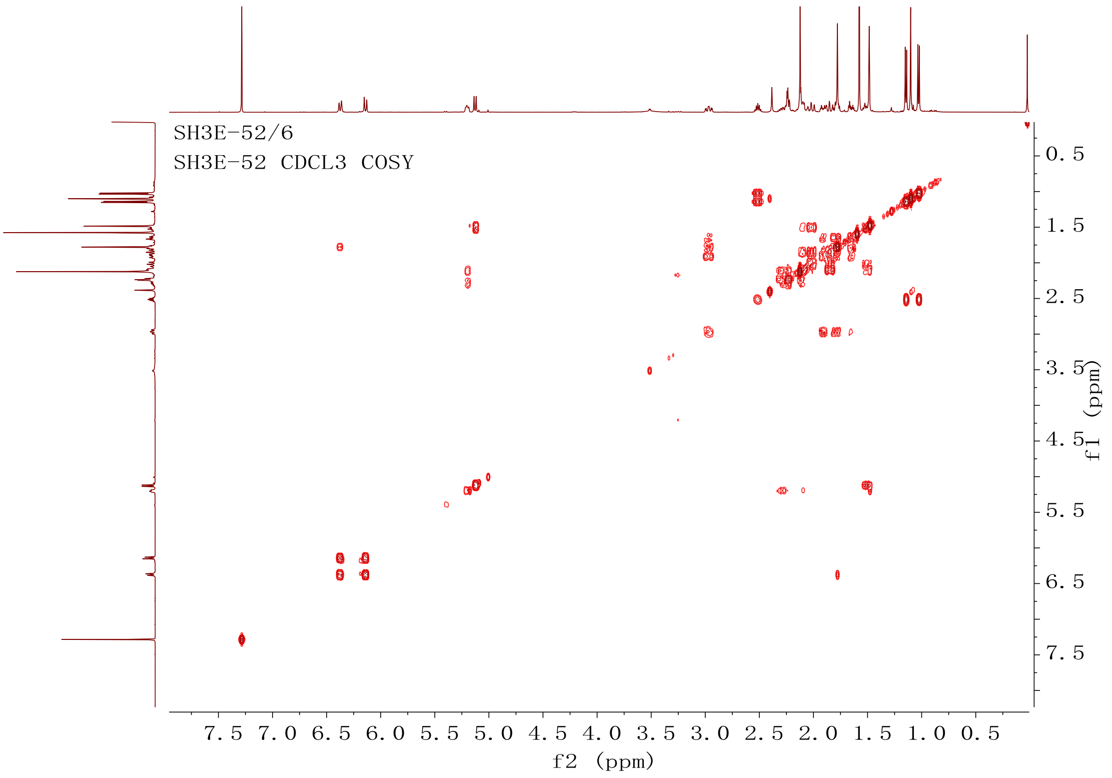

Figure S34. The ^{1}H-^{1}H COSY spectrum of compound **4** in $CDCl_3$

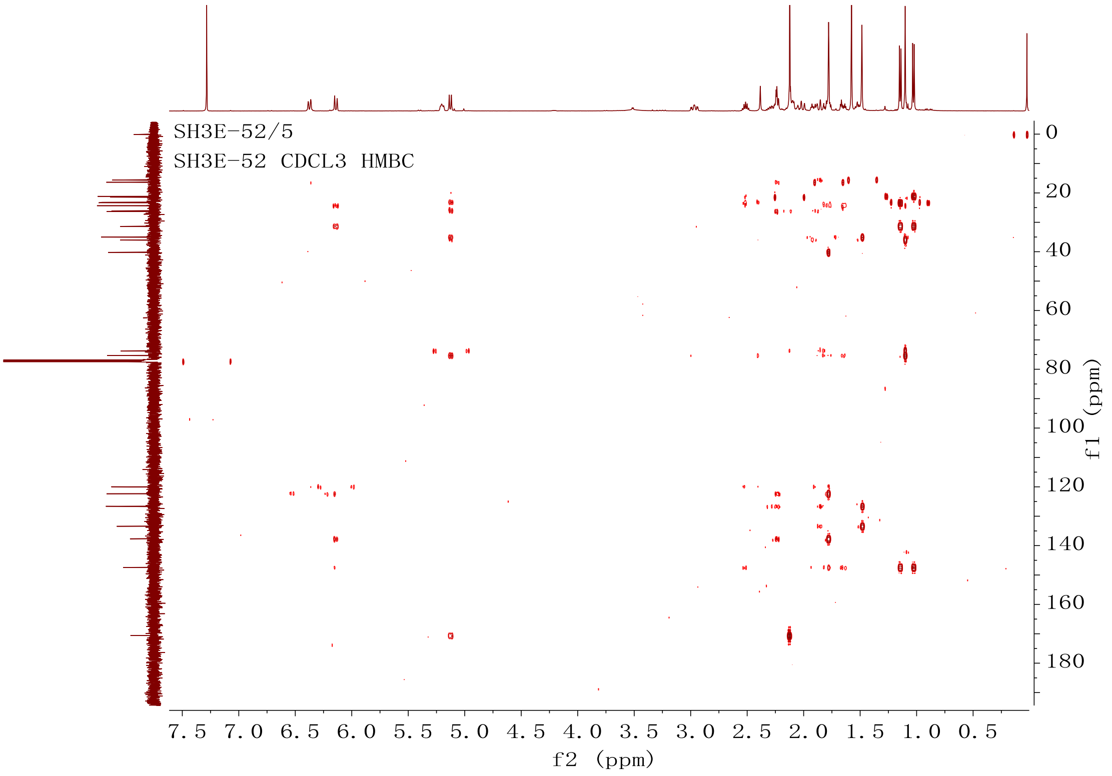

Figure S35. The HMBC spectrum of compound **4** in $CDCl_3$

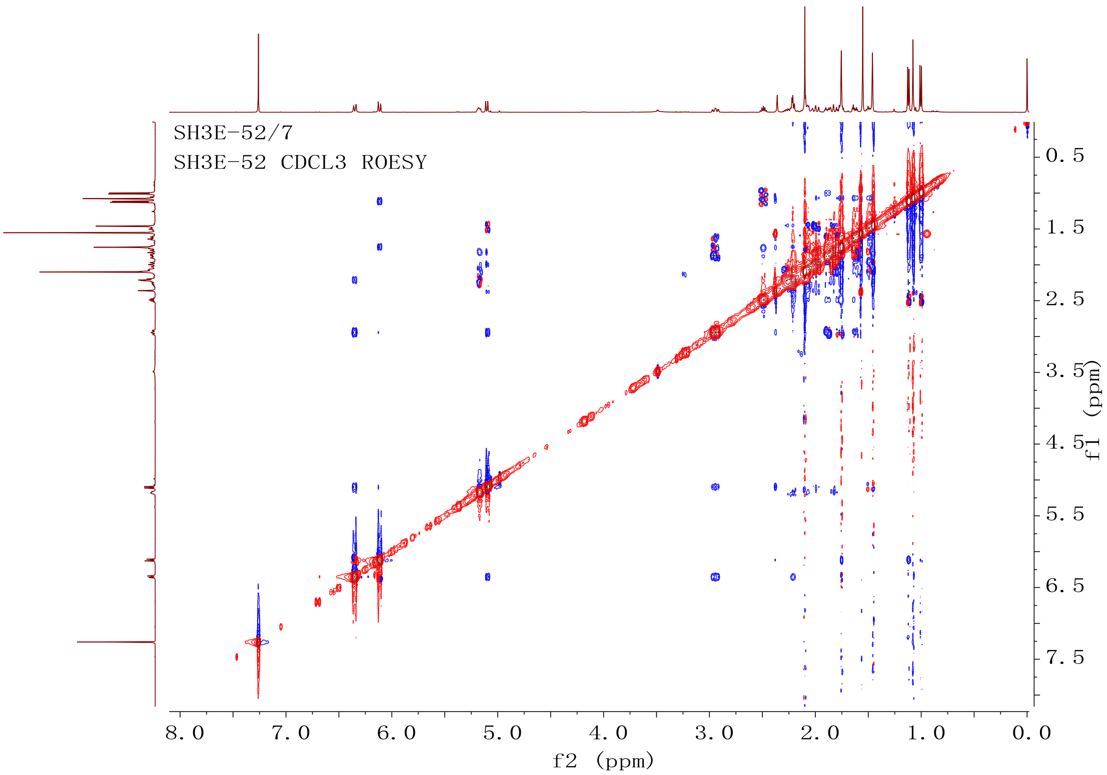

Figure S36. The ROESY spectrum of compound **4** in $CDCl_3$

Mass Spectrum SmartFormula Report

Analysis Info

Analysis Name	D:\Data\A501\WYL\2022\20220930\B502-SH3E-52_BC2_01_4852.d	Acquisition Date	2022-09-30 10:18:52
Method	lc-ms_as_ms-0.5MIN.m	Operator	Demo User
Sample Name	B502-SH3E-52	Instrument	compact 8255754.20156
Comment			

Acquisition Parameter

Source Type	ESI	Ion Polarity	Positive	Set Nebulizer	1.8 Bar
Focus	Not active	Set Capillary	4500 V	Set Dry Heater	220 °C
Scan Begin	50 m/z	Set End Plate Offset	-500 V	Set Dry Gas	4.0 l/min
Scan End	2500 m/z	Set Charging Voltage	2000 V	Set Divert Valve	Waste
		Set Corona	0 nA	Set APCI Heater	0 °C

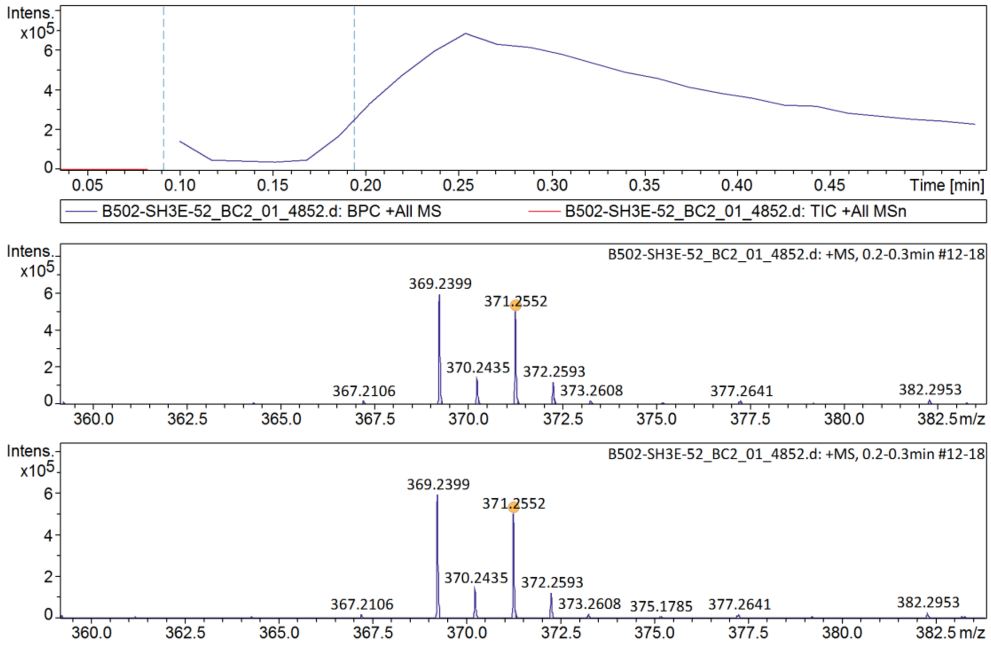

Meas. m/z	#	Ion Formula	m/z	err [ppm]	mSigma	# mSigma	Score	rdb	e⁻ Conf	N-Rule	Adduct
371.2552	1	C22H36NaO3	371.2557	1.2	2.3	1	100.00	5.0	even	ok	M+Na

Figure S37. The HRESIMS spectrum of compound **4**

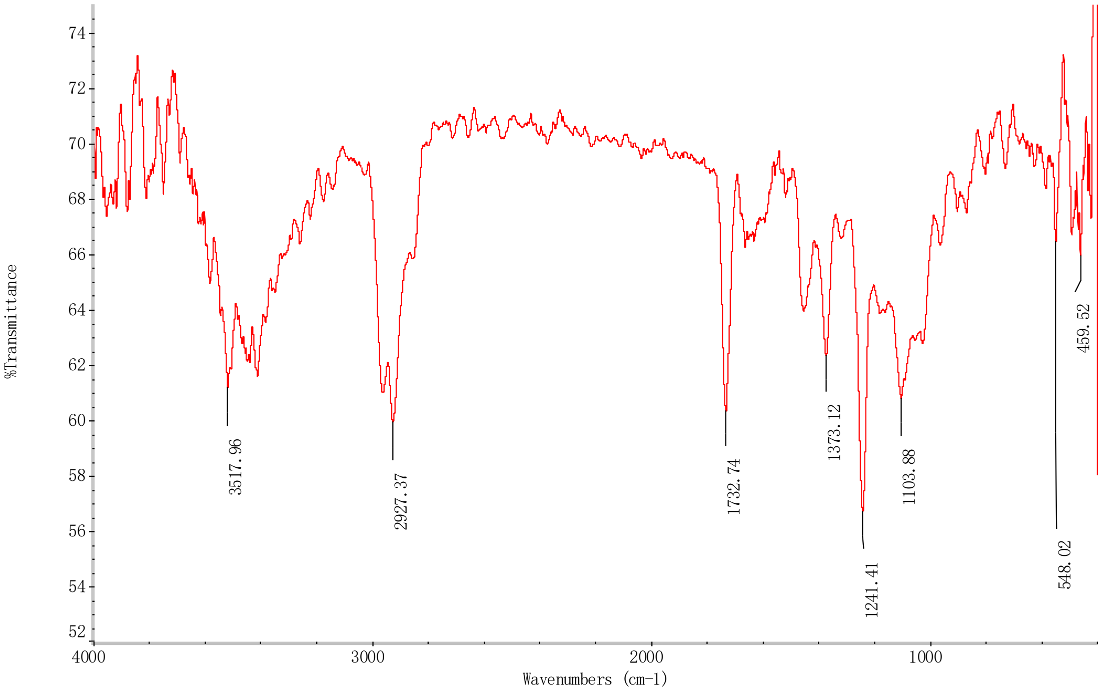

Figure S38. The IR spectrum of compound **4**

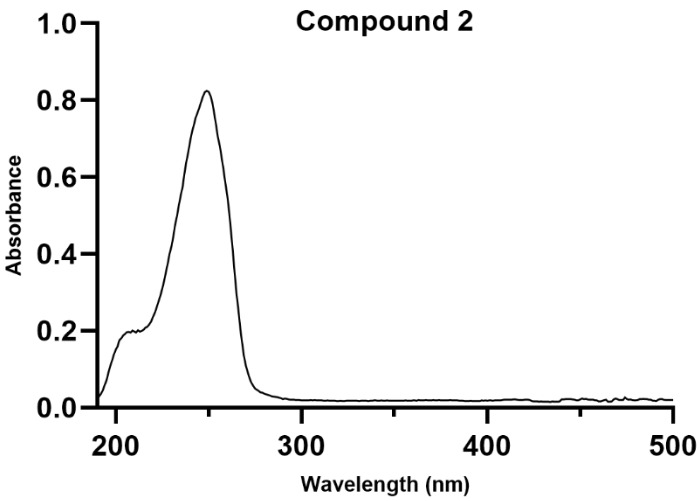

Figure S39. The UV spectrum of compound **4**

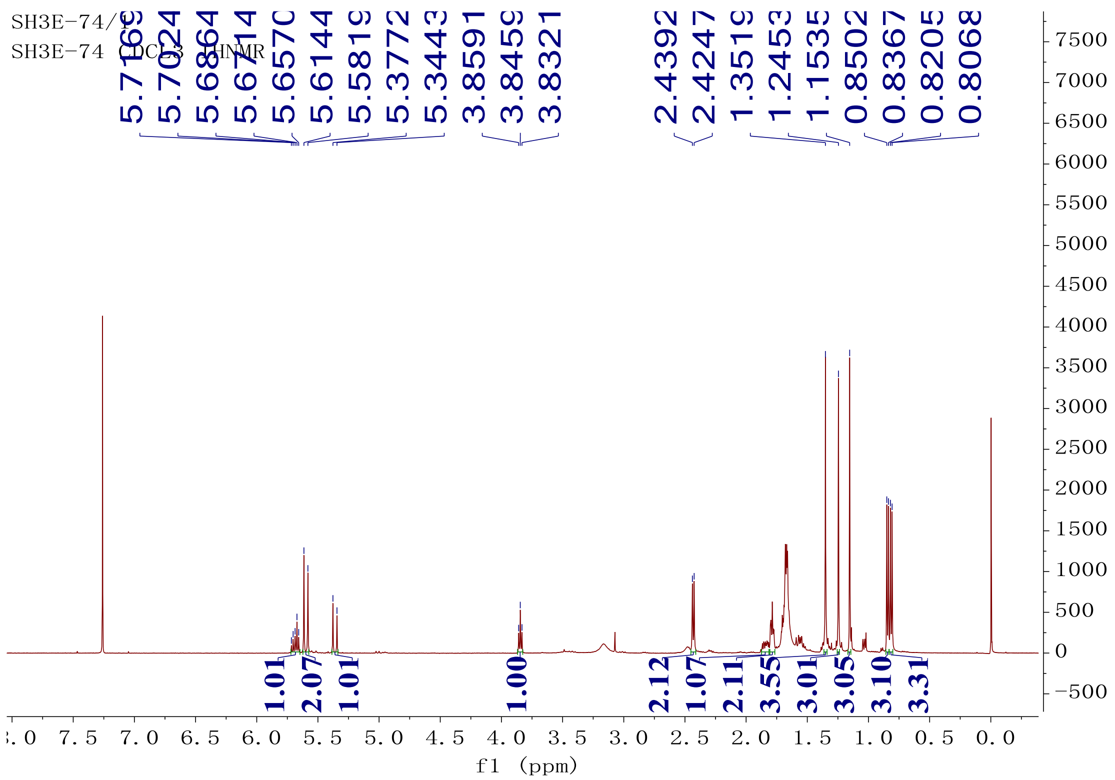

Figure S40. ^{1}H NMR spectrum (500 MHz, $CDCl_3$) of compound **5**

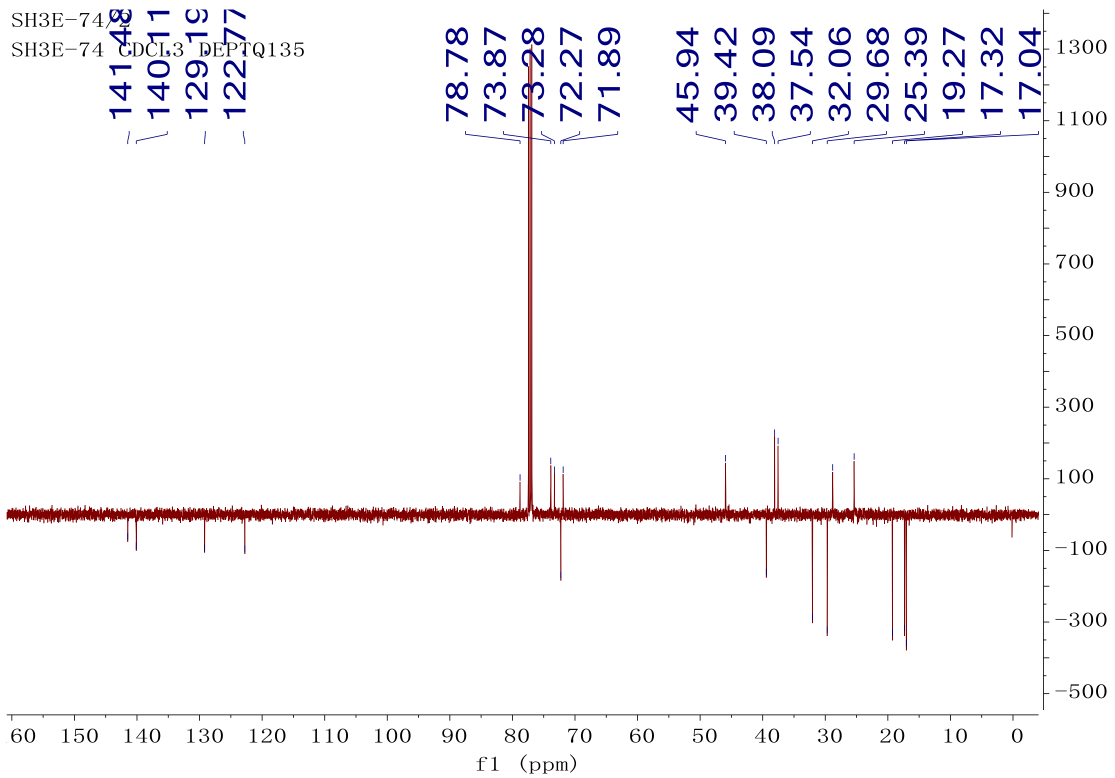

Figure S41. ^{13}C NMR spectrum (125 MHz, $CDCl_3$) of compound **5**

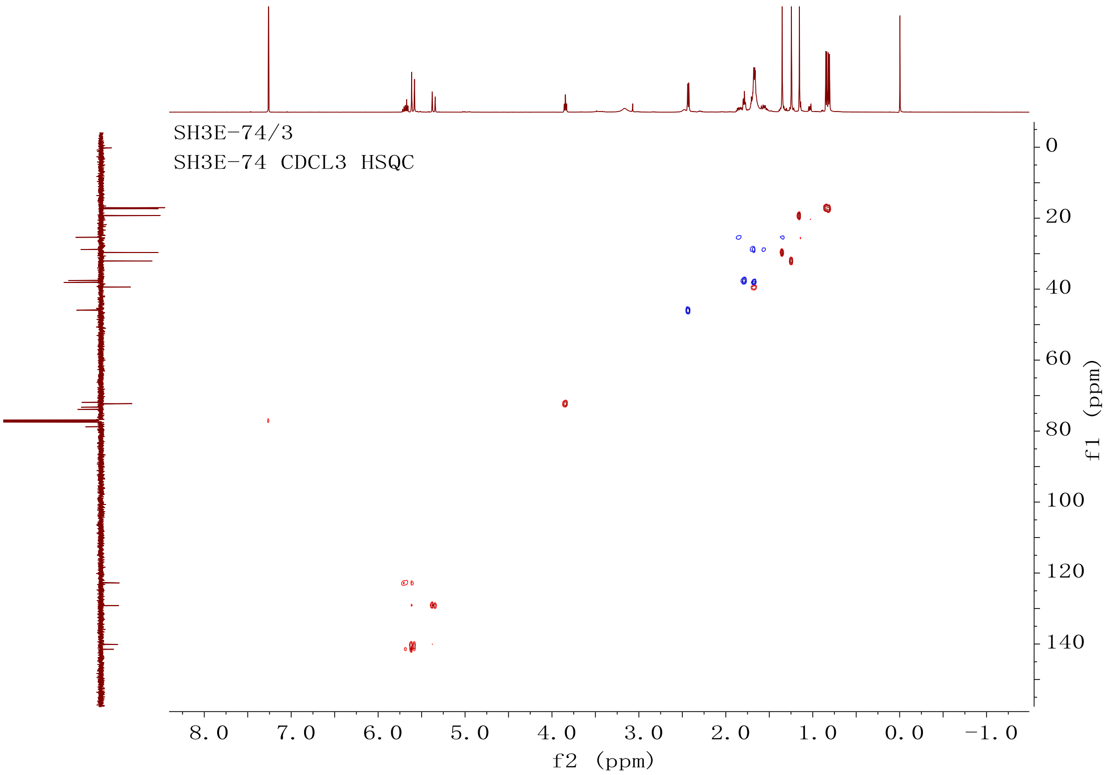

Figure S42. The HSQC spectrum of compound **5** in $CDCl_3$

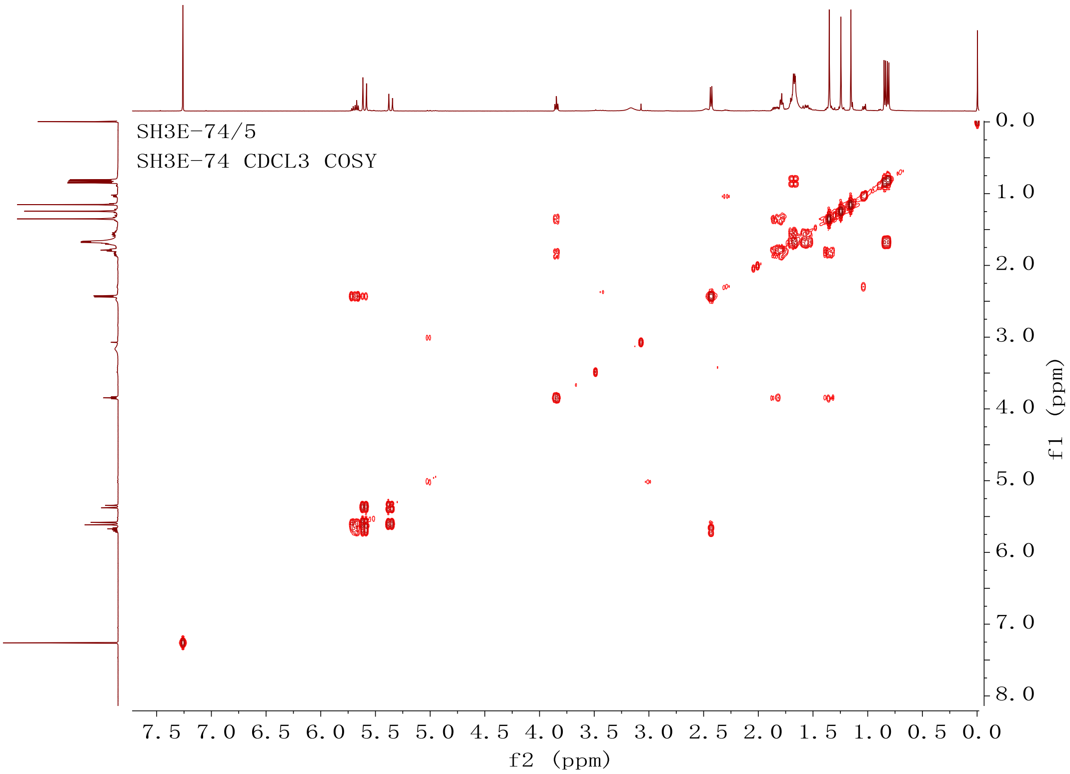

Figure S43. The ^1H-^1H COSY spectrum of compound **5** in $CDCl_3$

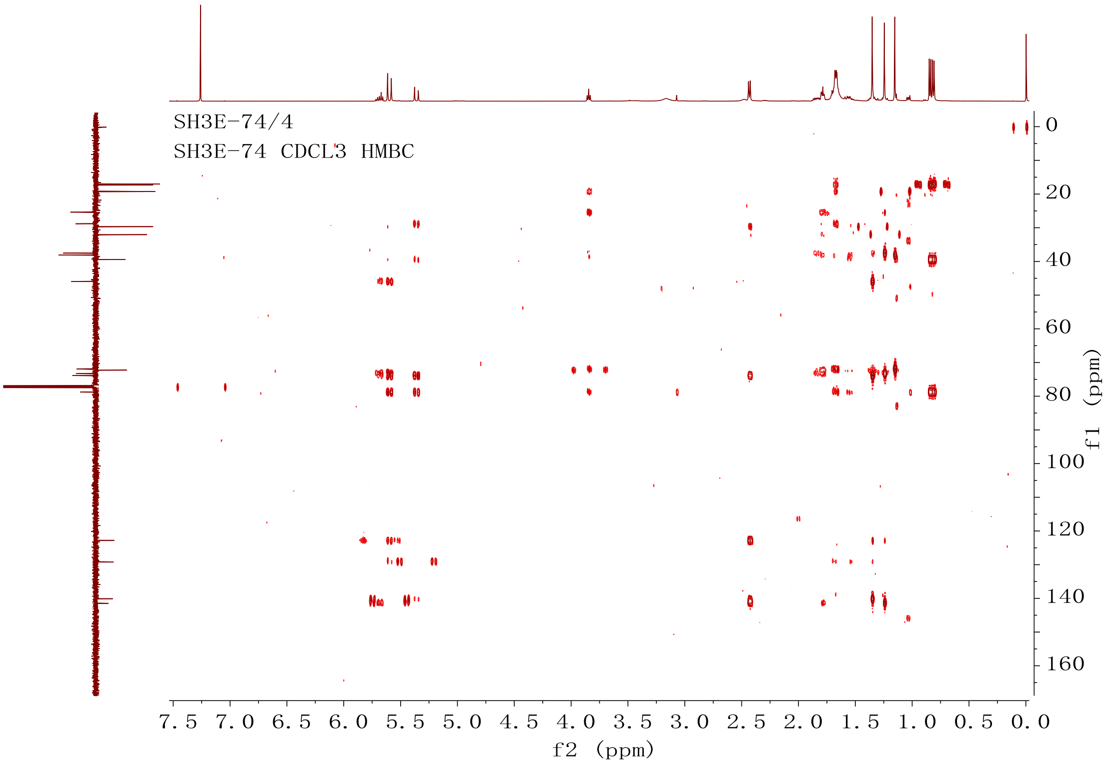

Figure S44. The HMBC spectrum of compound **5** in $CDCl_3$

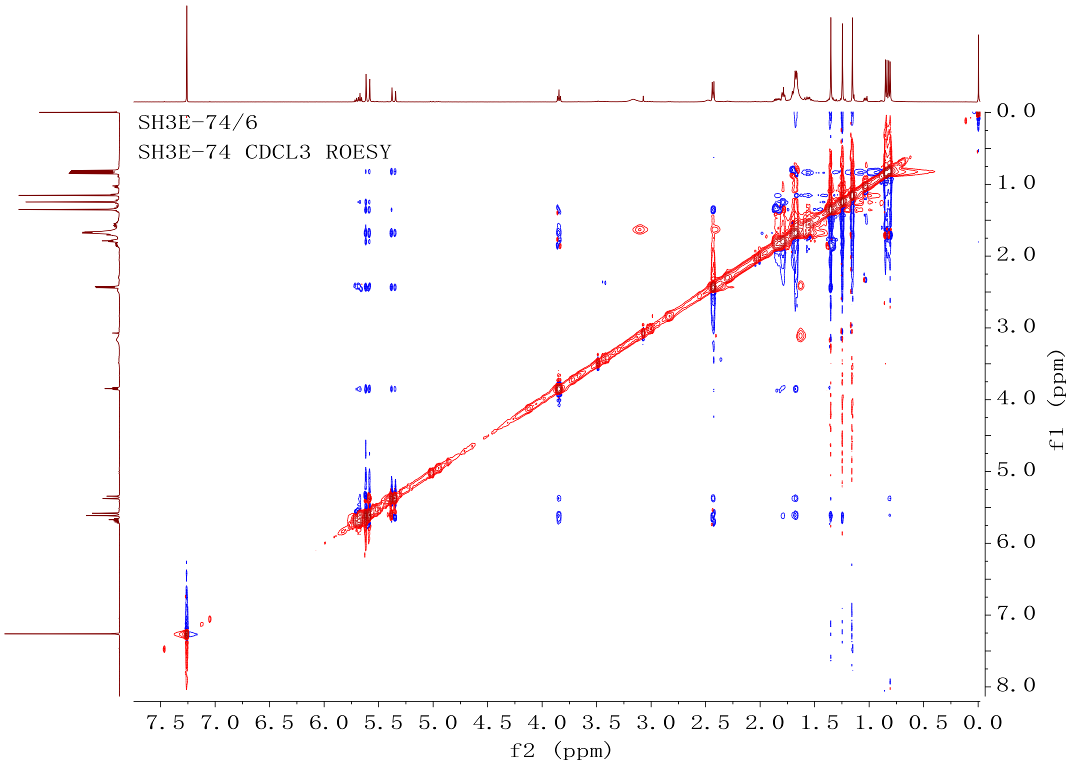

Figure S45. The ROESY spectrum of compound **5** in $CDCl_3$

Mass Spectrum SmartFormula Report

Analysis Info

		Acquisition Date	2022-11-22 16:52:51	
Analysis Name	D:\Data\A501\WYL\2022\20221122\B502-SH3E-74_GA6_01_5685.d			
Method	lc-ms_as_ms-0.5MIN-20221111.m	Operator	Demo User	
Sample Name	B502-SH3E-74	Instrument	compact	8255754.20156
Comment				

Acquisition Parameter

Source Type	ESI	Ion Polarity	Positive	Set Nebulizer	1.8 Bar
Focus	Not active	Set Capillary	3500 V	Set Dry Heater	220 °C
Scan Begin	50 m/z	Set End Plate Offset	-500 V	Set Dry Gas	4.0 l/min
Scan End	2500 m/z	Set Charging Voltage	2000 V	Set Divert Valve	Waste
		Set Corona	0 nA	Set APCI Heater	0 °C

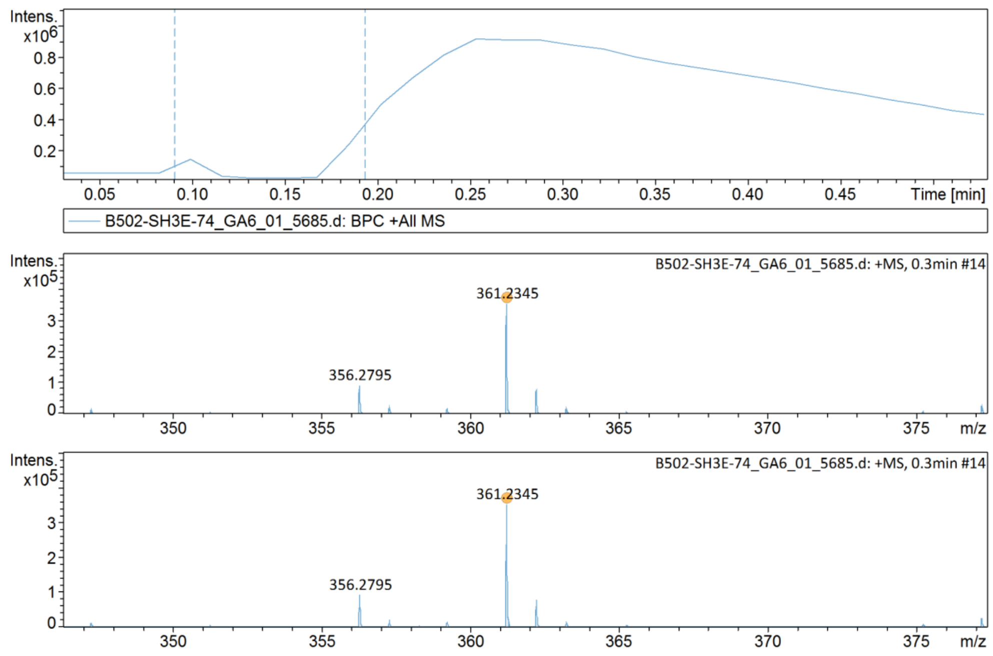

Meas. m/z	#	Ion Formula	m/z	err [ppm]	mSigma	# mSigma	Score	rdb	e⁻ Conf	N-Rule	Adduct
361.2345	1	C20H34NaO4	361.2349	1.2	4.7	1	100.00	4.0	even	ok	M+Na

Figure S46. The HRESIMS spectrum of compound **5**

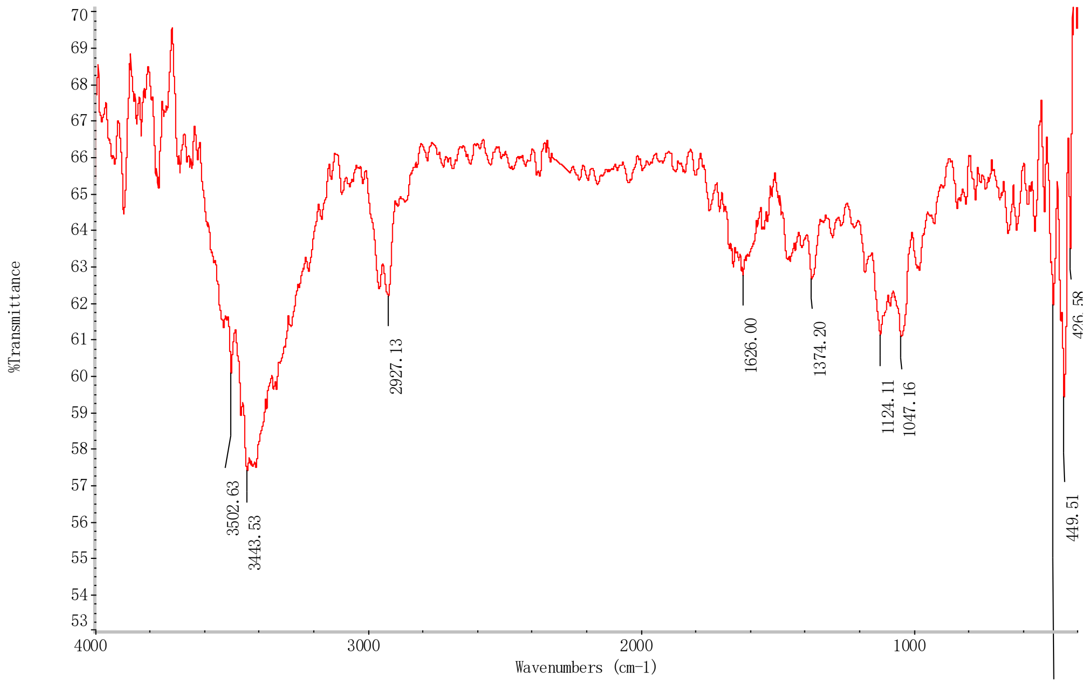

Figure S47. The IR spectrum of compound **5**

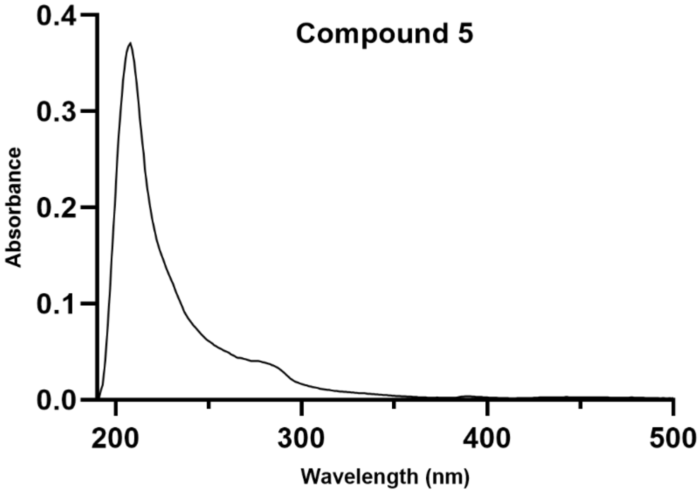

Figure S48. The UV spectrum of compound **5**

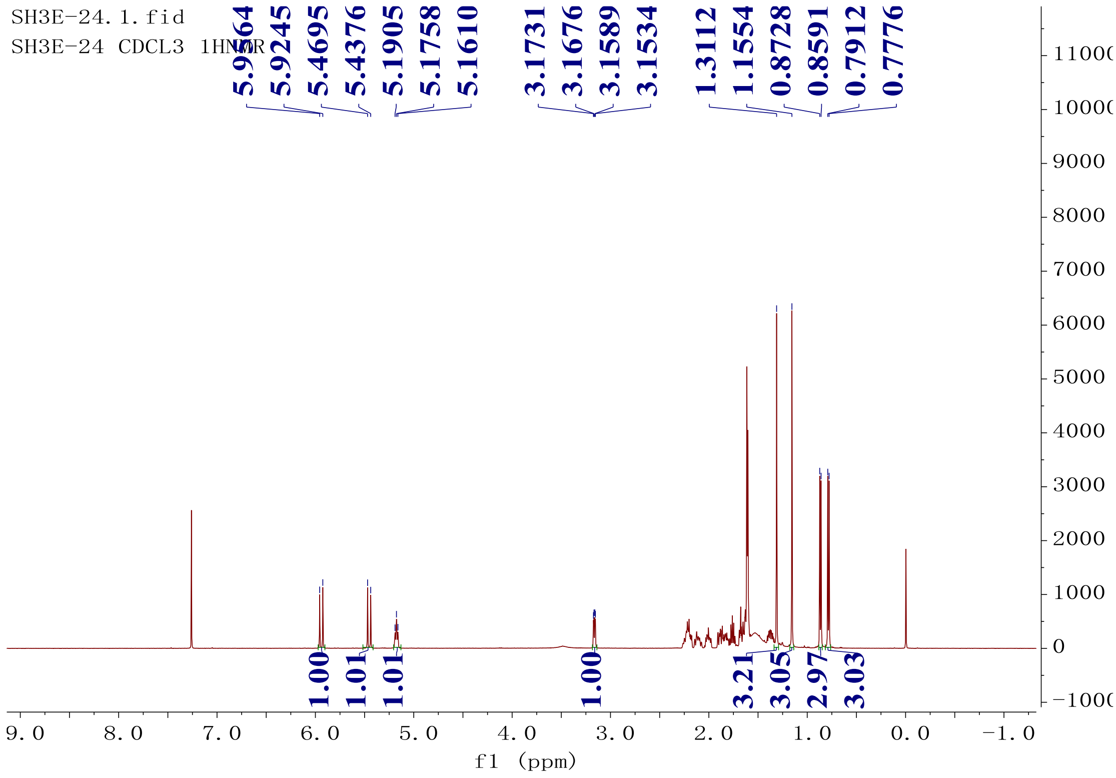

Figure S49. ^{1}H NMR spectrum (500 MHz, $CDCl_3$) of compound **6**

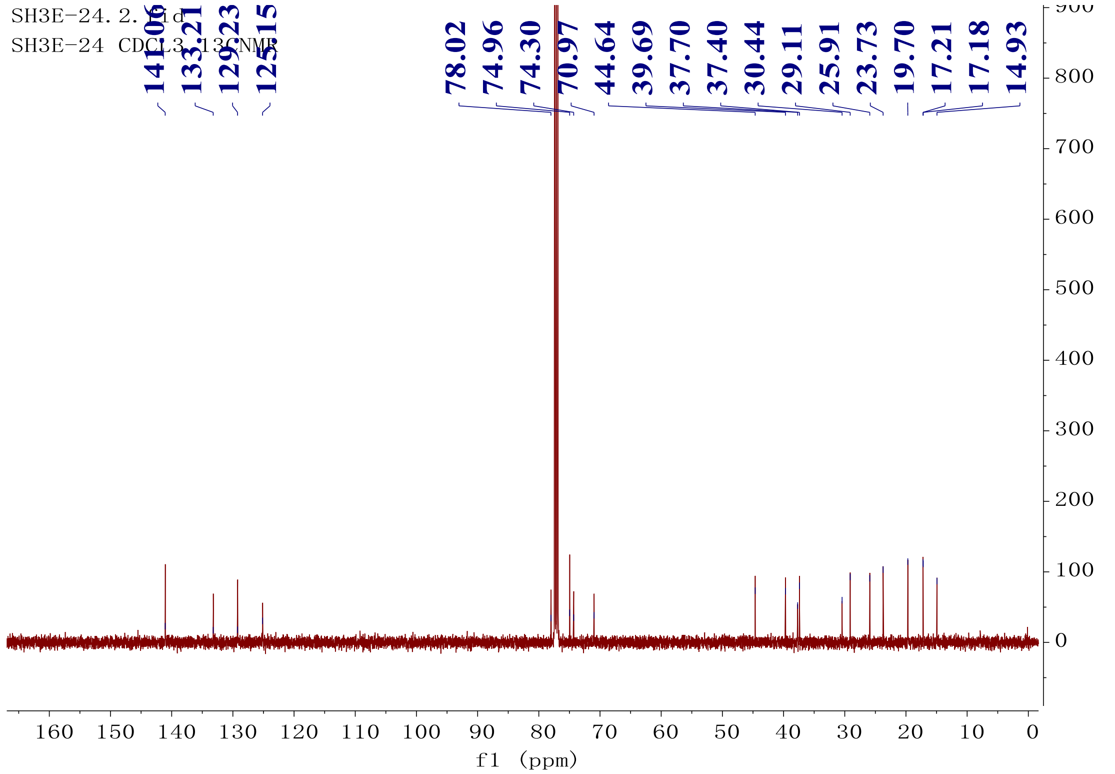

Figure S50. ^{13}C NMR spectrum (125 MHz, $CDCl_3$) of compound **6**

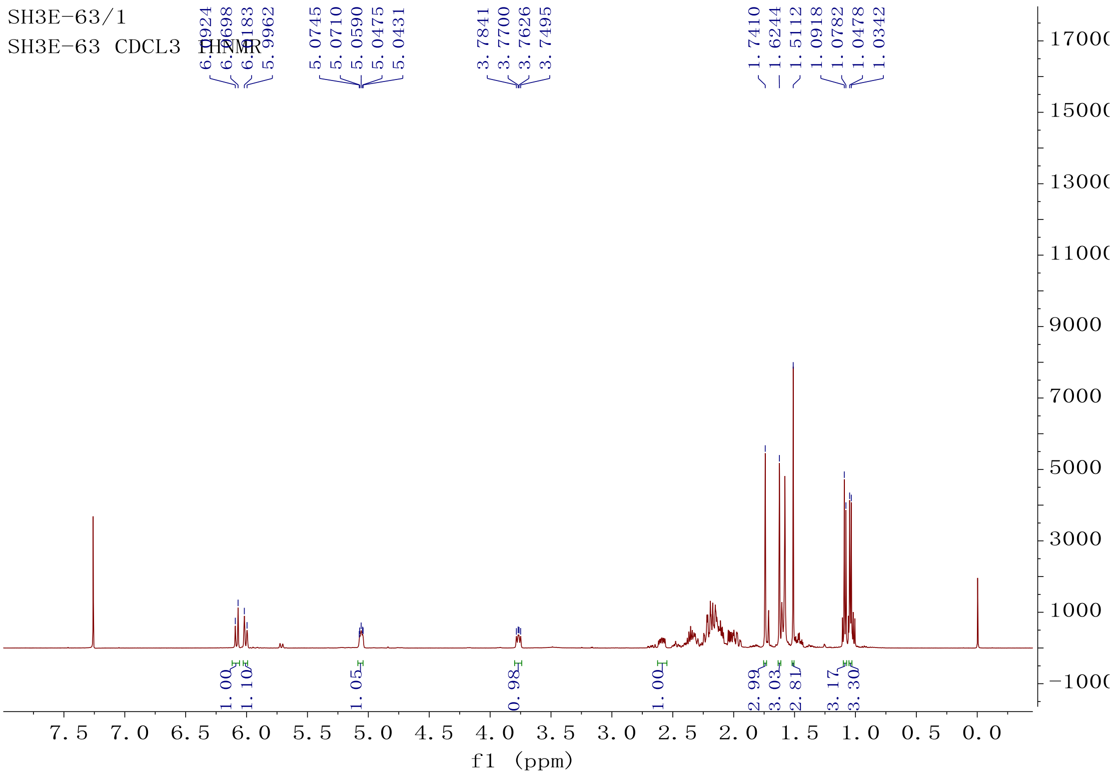

Figure S51. ^{1}H NMR spectrum (500 MHz, CDCl$_3$) of compound **7**

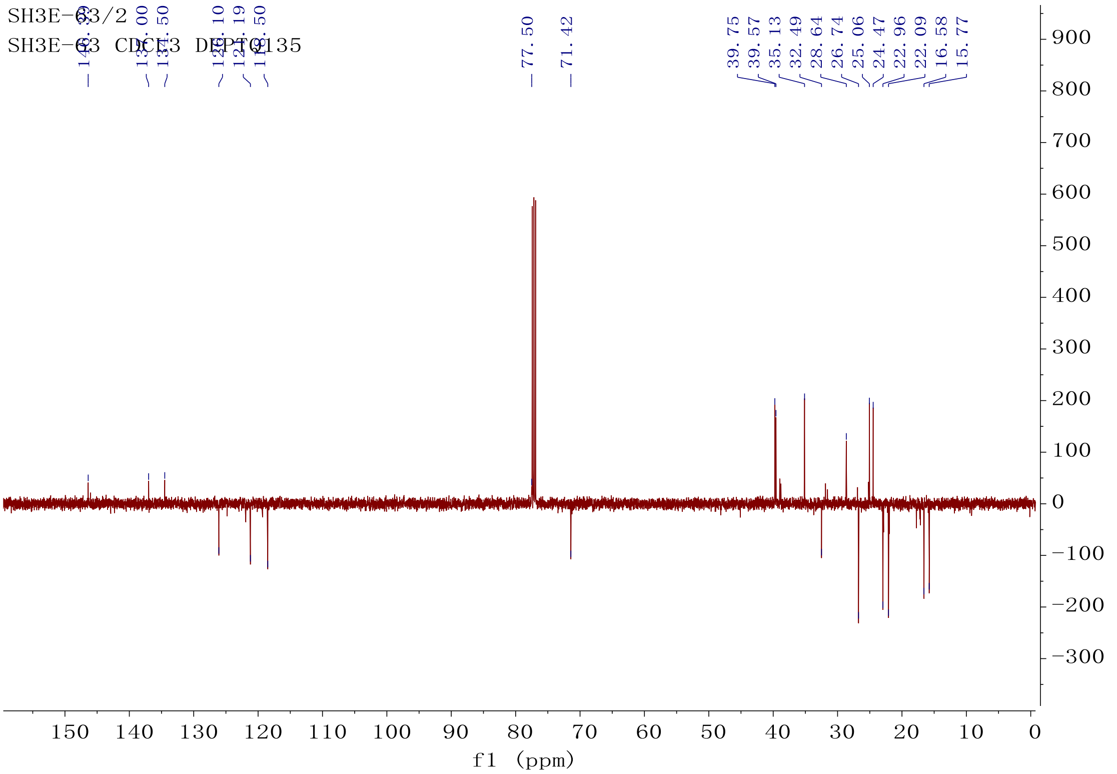

Figure S52. ^{13}C NMR spectrum (125 MHz, CDCl$_3$) of compound **7**

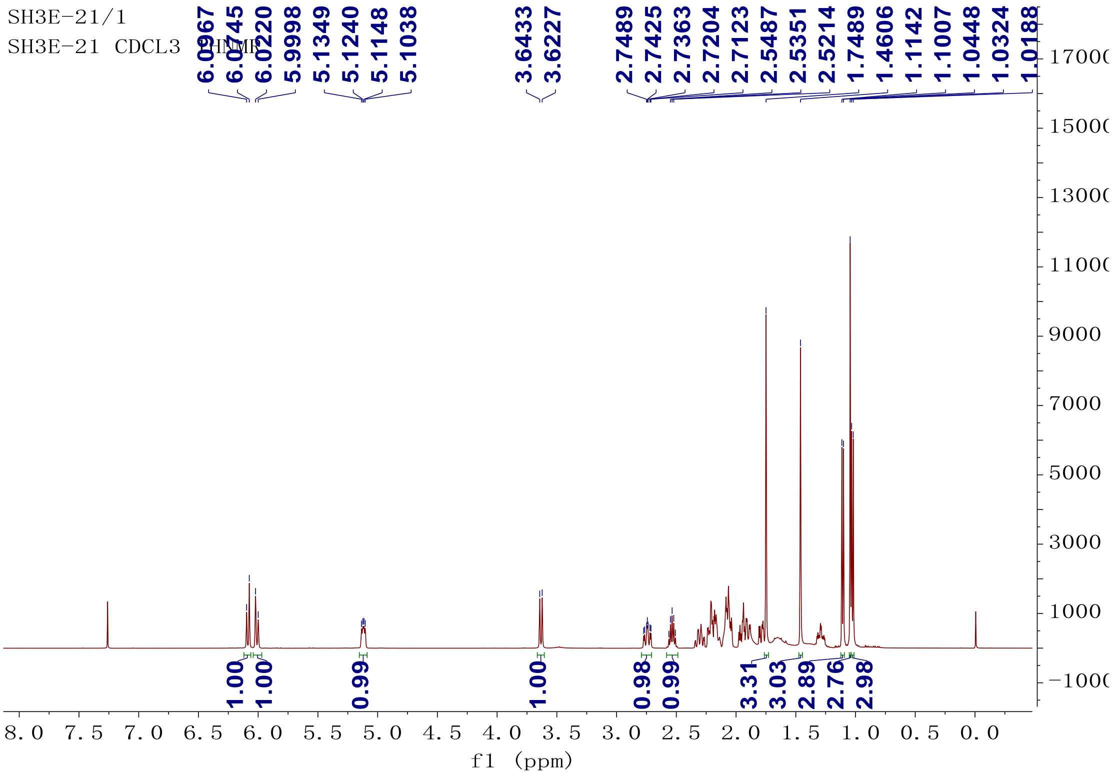

Figure S53. ^{1}H NMR spectrum (500 MHz, $CDCl_3$) of compound **8**

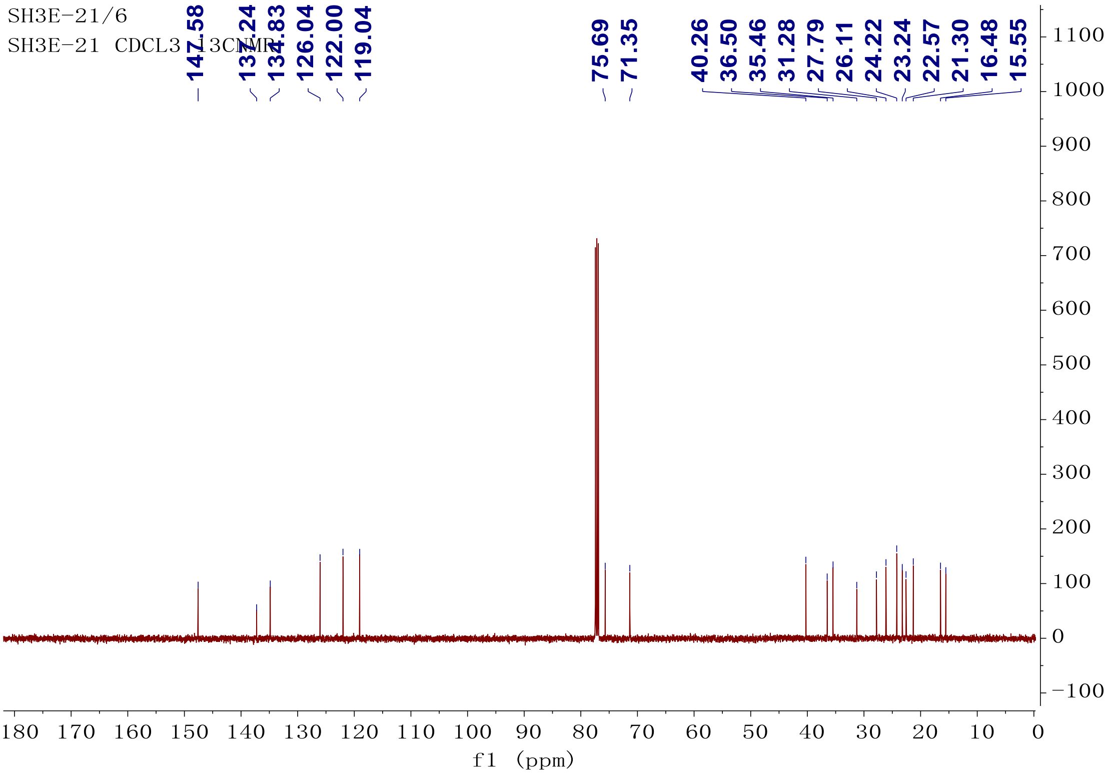

Figure S54. ^{13}C NMR spectrum (125 MHz, $CDCl_3$) of compound **8**

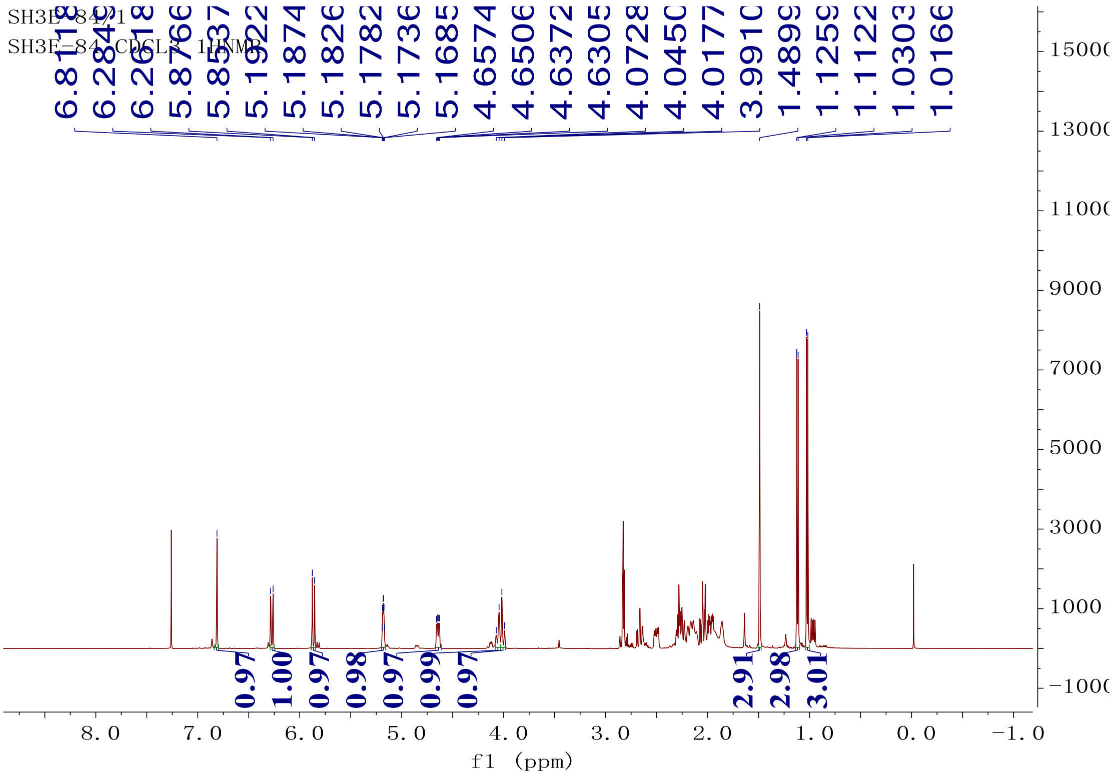

Figure S55. ^{1}H NMR spectrum (500 MHz, $CDCl_3$) of compound **9**

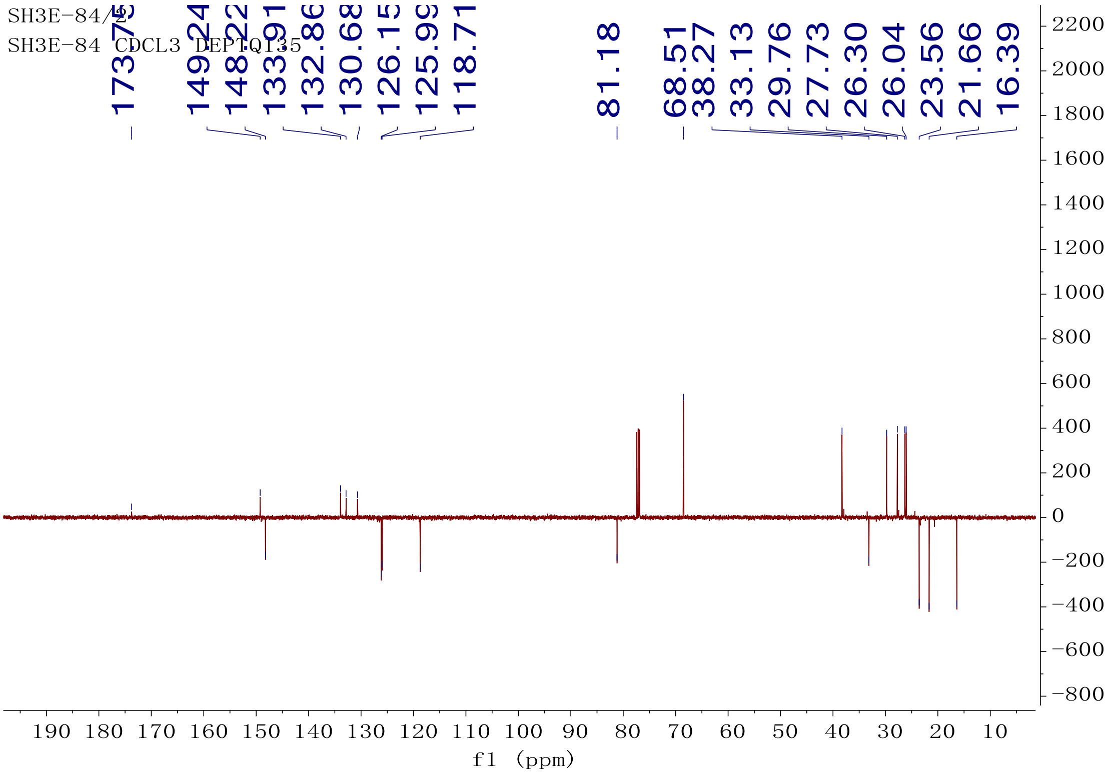

Figure S56. ^{13}C NMR spectrum (125 MHz, $CDCl_3$) of compound **9**

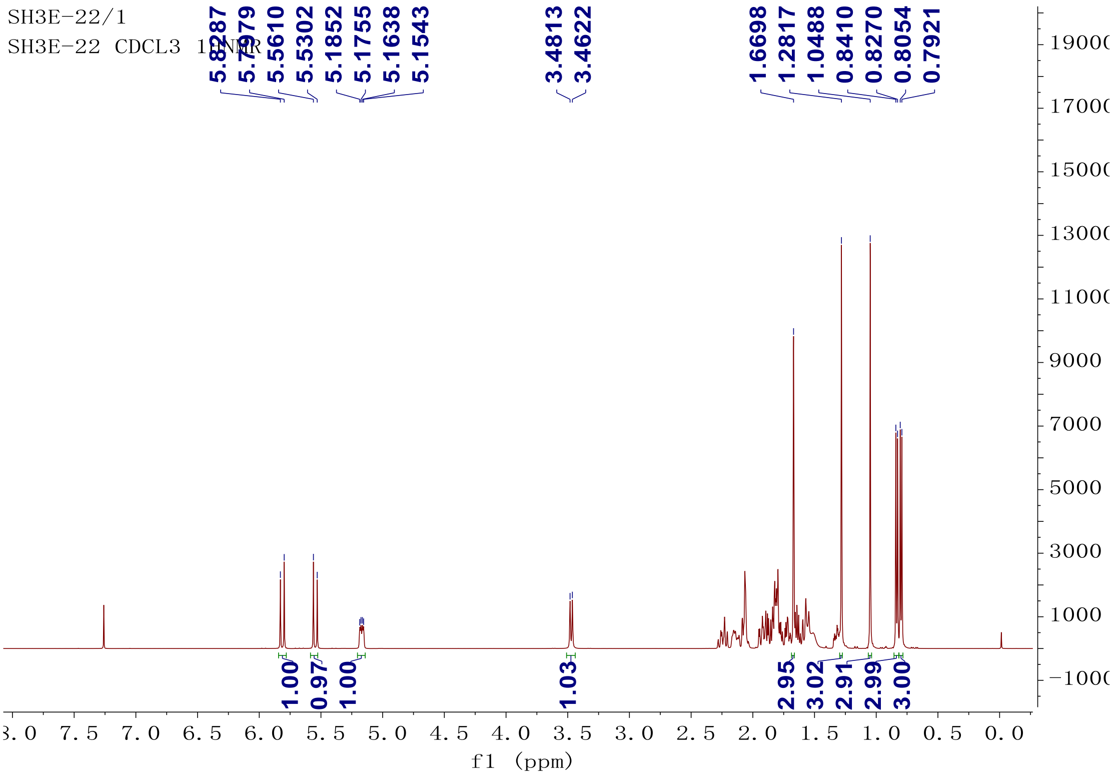

Figure S57. ^{1}H NMR spectrum (500 MHz, $CDCl_3$) of compound **10**

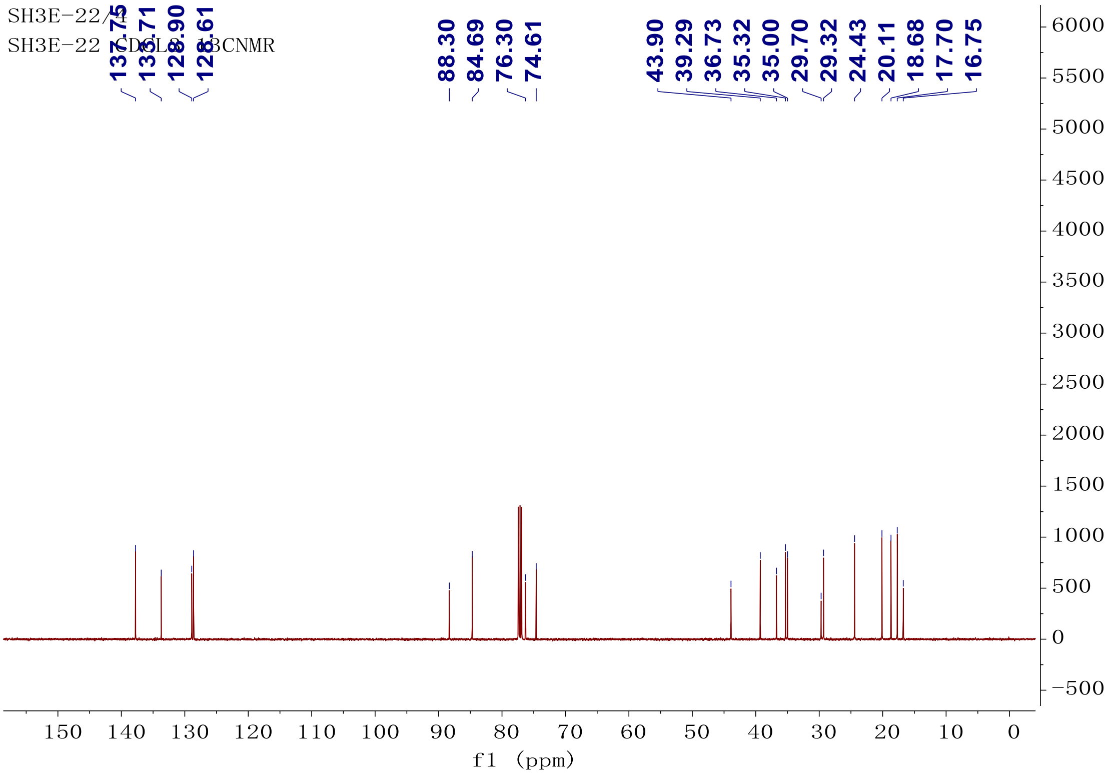

Figure S58. ^{13}C NMR spectrum (125 MHz, $CDCl_3$) of compound **10**

Table S1. Crystal data and structure refinement for compound **1**.

Identification code	SH3E-65
Empirical formula	$C_{21}H_{38}O_4$
Formula weight	354.51
Temperature/K	169.99(10)
Crystal system	monoclinic
Space group	$P2_1$
a/Å	11.1337(4)
b/Å	9.6614(3)
c/Å	19.4879(8)
α/°	90
β/°	93.623(3)
γ/°	90
Volume/Å^3	2092.07(13)
Z	4
ρ_{calc}g/cm^3	1.126
μ/mm^{-1}	0.598
F(000)	784.0
Crystal size/mm^3	0.15 × 0.12 × 0.1
Radiation	Cu Kα (λ = 1.54184)
2Θ range for data collection/°	4.544 to 133.186
Index ranges	$-13 \leq h \leq 11$, $-11 \leq k \leq 11$, $-23 \leq l \leq 23$
Reflections collected	16109
Independent reflections	7268 [R_{int} = 0.0734, R_{sigma} = 0.0639]
Data/restraints/parameters	7268/1/477
Goodness-of-fit on F^2	1.058
Final R indexes [I>=2σ (I)]	R_1 = 0.0708, wR_2 = 0.1855
Final R indexes [all data]	R_1 = 0.0795, wR_2 = 0.1895
Largest diff. peak/hole / e Å^{-3}	0.36/-0.33
Flack/Hooft parameter	0.0(2)/-0.06(12)

Table S2. Conformational analysis of the optimized isomers of **2**.

Conformations	*G* (hartree)	*ΔG* (kcal/mol)	Boltzmann distributions (%)
1*R*,4*R*,11*R*,12*S*-**2-1**	-1047.416628	0	30.1
1*R*,4*R*,11*R*,12*S*-**2-2**	-1047.416527	0.06337845	27.1
1*R*,4*R*,11*R*,12*S*-**2-3**	-1047.416	0.3940759	15.5
1*R*,4*R*,11*R*,12*S*-**2-4**	-1047.415128	0.9412641	6.1
1*R*,4*R*,11*R*,12*S*-**2-5**	-1047.415114	0.95004923	6.1
1*R*,4*R*,11*R*,12*S*-**2-6**	-1047.415083	0.96950202	5.9
1*R*,4*R*,11*R*,12*S*-**2-7**	-1047.414822	1.13328198	4.4
1*R*,4*R*,11*R*,12*S*-**2-8**	-1047.414287	1.46899951	2.5
1*R*,4*R*,11*R*,12*S*-**2-9**	-1047.414191	1.52924041	2.3

Table S3. The coordinates of the optimized conformers of **2**.

	1*R*,4*R*,11*R*,12*S*-**2-1**			1*R*,4*R*,11*R*,12*S*-**2-2**			1*R*,4*R*,11*R*,12*S*-**2-3**			1*R*,4*R*,11*R*,12*S*-**2-4**			1*R*,4*R*,11*R*,12*S*-**2-5**			1*R*,4*R*,11*R*,12*S*-**2-6**			1*R*,4*R*,11*R*,12*S*-**2-7**			1*R*,4*R*,11*R*,12*S*-**2-8**			1*R*,4*R*,11*R*,12*S*-**2-9**		
	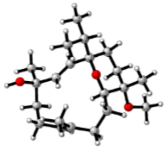			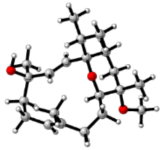			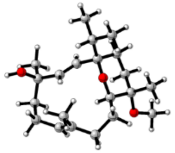			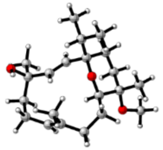			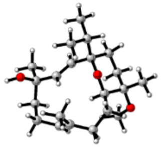			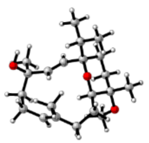			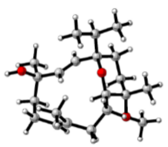			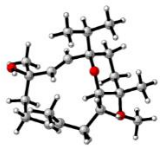			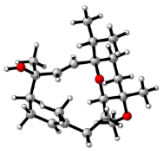		
C	-1.30764	-2.70305	-0.1605	-1.80935	-2.54725	0.15364	-1.77386	-2.54968	0.10587	-1.80068	-2.54341	0.11662	-1.39305	-2.63306	0.0407	-1.9107	-2.38688	0.31785	-1.73112	-2.4386	0.17069	-1.74535	-2.44299	0.17108	-1.90478	-2.37345	0.25536
C	-2.74277	-2.44712	-0.52086	-3.21861	-2.0671	-0.04671	-3.18287	-2.08633	-0.1281	-3.21184	-2.07371	-0.09405	-2.82204	-2.28852	-0.26636	-3.28343	-1.79774	0.16011	-3.12037	-2.02376	-0.22075	-3.14292	-2.02364	-0.18746	-3.27246	-1.78529	0.06047
C	-2.93673	-1.31666	-1.54904	-3.3683	-1.11383	-1.24836	-3.30617	-1.08023	-1.28856	-3.35299	-1.08241	-1.26728	-2.98116	-1.22185	-1.36736	-3.40645	-0.8622	-1.05856	-3.1693	-1.00728	-1.37802	-3.21511	-1.02437	-1.3604	-3.34598	-0.7859	-1.11027
C	-2.95238	0.12338	-0.98117	-3.07717	0.37103	-0.96854	-3.08519	0.40343	-0.91392	-3.08023	0.40464	-0.94879	-2.89309	0.25111	-0.90013	-2.99263	0.60187	-0.82684	-2.96474	0.47908	-0.99305	-2.96787	0.46663	-1.02935	-2.98323	0.67765	-0.76591
C	-1.67367	0.46996	-0.26119	-1.7557	0.58775	-0.27215	-1.7647	0.63073	-0.22212	-1.7526	0.61558	-0.26056	-1.58125	0.55783	-0.2234	-1.62616	0.73135	-0.19858	-1.6226	0.72266	-0.35072	-1.61972	0.69756	-0.39013	-1.6148	0.79816	-0.1436
C	-0.76182	1.35808	-0.64022	-0.72081	1.30009	-0.70602	-0.72312	1.31062	-0.68695	-0.71487	1.3192	-0.69854	-0.63293	1.38104	-0.6559	-0.56361	1.35432	-0.69831	-0.62204	1.44561	-0.83982	-0.62539	1.44822	-0.84933	-0.5434	1.37725	-0.67297
C	0.46314	1.73374	0.17433	0.51978	1.56724	0.13647	0.53422	1.57918	0.13084	0.53237	1.57683	0.13738	0.62951	1.71588	0.11937	0.737	1.54073	0.07268	0.6908	1.71947	-0.12206	0.67277	1.72085	-0.10599	0.77681	1.54336	0.0696
C	1.63044	2.12743	-0.75039	1.70871	1.9404	-0.76307	1.7126	1.92427	-0.7939	1.72111	1.93452	-0.76854	1.7844	2.04655	-0.8464	1.90293	1.785	-0.90135	1.83577	1.74995	-1.15342	1.83241	1.78733	-1.11855	1.92941	1.74221	-0.9306
C	2.26663	0.91603	-1.43502	2.24898	0.72837	-1.52391	2.23379	0.69386	-1.53866	2.2477	0.7142	-1.52577	2.33016	0.79581	-1.53845	2.28988	0.50655	-1.64788	2.16382	0.35326	-1.68479	2.18582	0.40615	-1.6731	2.27142	0.44269	-1.6629
C	2.66936	-0.15895	-0.41473	2.58953	-0.4234	-0.56705	2.57656	-0.44238	-0.56415	2.58191	-0.43524	-0.56392	2.72509	-0.27407	-0.5133	2.58929	-0.63158	-0.66435	2.46133	-0.61634	-0.52922	2.48107	-0.5817	-0.53291	2.55996	-0.6866	-0.66582
C	1.40087	-0.50722	0.38807	1.31274	-0.7432	0.23815	1.30606	-0.73403	0.26068	1.30462	-0.73977	0.2461	1.47353	-0.56666	0.3439	1.33184	-0.82869	0.21381	1.22858	-0.59745	0.39608	1.23541	-0.60147	0.3753	1.31432	-0.83444	0.23762
C	1.58108	-1.59258	1.4556	1.47726	-1.86588	1.26817	1.46419	-1.84594	1.30363	1.45934	-1.86174	1.27885	1.66589	-1.63282	1.42695	1.4629	-1.93364	1.26548	1.29672	-1.52889	1.61074	1.30906	-1.55266	1.57477	1.42584	-1.92993	1.3018
C	-0.71392	-2.63502	1.03657	-1.02246	-2.4471	1.23074	-1.02258	-2.46247	1.20907	-1.03148	-2.46896	1.20827	-0.72409	-2.5131	1.19324	-1.07198	-2.31836	1.35783	-1.11646	-2.31479	1.35289	-1.10532	-2.34325	1.34193	-1.10531	-2.32581	1.32684
C	0.77351	-2.87255	1.15977	0.39666	-2.96091	1.17486	0.40395	-2.95831	1.18654	0.3932	-2.96771	1.15752	0.74524	-2.85889	1.25513	0.29857	-2.94495	1.26138	0.33031	-2.72734	1.50301	0.34595	-2.75171	1.45105	0.26895	-2.94919	1.26767
C	-1.41485	-2.25965	2.31524	-1.4214	-1.79104	2.52528	-1.46936	-1.8378	2.50334	-1.45519	-1.85663	2.51644	-1.31244	-1.97496	2.47022	-1.35944	-1.59126	2.64393	-1.74128	-1.6985	2.57662	-1.7052	-1.7583	2.59358	-1.43948	-1.62371	2.6153
C	0.13285	2.9012	1.15139	0.19154	2.67559	1.17892	0.23442	2.71098	1.15685	0.21896	2.6925	1.17666	0.38022	2.91109	1.08673	0.55839	2.70537	1.08964	0.57686	3.07462	0.6459	0.53144	3.05769	0.68917	0.64712	2.73015	1.06821
C	-0.31363	4.17314	0.42238	1.32667	2.92843	2.17634	1.38624	2.96781	2.13402	1.36356	2.94331	2.16375	-0.02879	4.19138	0.35039	1.76369	2.89221	2.01701	-0.53527	3.05057	1.70169	-0.61211	3.00886	1.71023	1.87448	2.90256	1.96917
C	-0.89824	2.50966	2.2129	-0.24817	3.9863	0.51911	-0.19753	4.01475	0.47851	-0.21941	4.00225	0.51422	-0.63689	2.58418	2.18323	0.19304	4.02737	0.4074	1.89329	3.50183	1.3111	1.82665	3.47112	1.40284	0.30197	4.04888	0.36942
C	-3.29776	1.10416	-2.1026	-3.22298	1.17443	-2.26109	-3.27559	1.27596	-2.15508	-3.24351	1.23206	-2.21748	-3.19583	1.17581	-2.07967	-3.14065	1.38745	-2.13015	-3.21415	1.3553	-2.22145	-3.19476	1.30171	-2.28351	-3.16071	1.55144	-2.00829
O	-3.96161	0.21705	0.05495	-4.11735	0.77818	-0.04131	-4.06154	0.78553	0.08715	-4.11652	0.89309	-0.0558	-3.87489	0.47777	0.14187	-3.95348	1.10478	0.13796	-3.91157	0.83789	0.04394	-3.98362	0.9232	-0.09613	-3.87167	1.1513	0.27655
C	3.81561	0.31397	0.48311	3.77117	-0.07324	0.34147	3.77255	-0.08542	0.32261	3.76966	-0.08984	0.33851	3.91092	0.18066	0.33321	3.83014	-0.32913	0.17182	3.753	-0.24651	0.2048	3.75599	-0.20622	0.22743	3.82524	-0.40372	0.13969
O	2.98223	-1.39869	-1.07558	2.8186	-1.64031	-1.30113	2.78562	-1.67576	-1.2764	2.79806	-1.65851	-1.29124	3.17924	-1.49331	-1.13663	2.91205	-1.86522	-1.33808	2.48656	-1.97737	-0.99805	2.53388	-1.93244	-1.02785	2.83411	-1.94094	-1.32309
O	0.88193	0.65936	1.03959	0.85333	0.41696	0.94242	0.86953	0.44157	0.95304	0.85711	0.4268	0.94742	1.02534	0.63688	0.99041	1.01391	0.39229	0.90233	0.9722	0.72831	0.88173	0.947	0.7103	0.88108	1.04272	0.40391	0.91417
C	4.1442	-1.41436	-1.88755	3.98039	-1.68998	-2.11185	3.93456	-1.75085	-2.10322	3.95465	-1.72173	-2.10848	2.40606	-2.03223	-2.1988	2.04714	-2.31418	-2.37115	3.53694	-2.32877	-1.88262	3.60469	-2.25149	-1.90021	1.94023	-2.37886	-2.3359
H	-0.67815	-2.9672	-1.01116	-1.38188	-3.02984	-0.72626	-1.31298	-3.00893	-0.76957	-1.35617	-2.99472	-0.77095	-0.83993	-3.0218	-0.81543	-1.56417	-2.93189	-0.56137	-1.15587	-2.88353	-0.64219	-1.18758	-2.86873	-0.66354	-1.52546	-2.90058	-0.62125
H	-3.15487	-3.36358	-0.96154	-3.85635	-2.93937	-0.23778	-3.79572	-2.96078	-0.37992	-3.83501	-2.94579	-0.32681	-3.32716	-3.19794	-0.61446	-3.99658	-2.61924	0.01714	-3.66643	-2.91689	-0.54954	-3.70242	-2.9162	-0.49363	-3.97057	-2.60309	-0.1568
H	-3.35346	-2.23002	0.35626	-3.62374	-1.58476	0.84387	-3.62967	-1.64976	0.76643	-3.642	-1.64869	0.8166	-3.36483	-1.95932	0.62056	-3.61081	-1.26383	1.05306	-3.67943	-1.6186	0.62343	-3.68595	-1.62705	0.67388	-3.64574	-1.29864	0.96265
H	-3.89813	-1.46822	-2.05511	-4.39791	-1.15921	-1.61725	-4.31351	-1.15025	-1.71718	-4.37573	-1.12765	-1.65371	-3.96526	-1.34611	-1.83557	-4.45031	-0.83637	-1.38705	-4.15378	-1.07672	-1.85698	-4.21555	-1.07891	-1.80079	-4.36986	-0.77316	-1.50354
H	-2.16905	-1.37959	-2.32732	-2.72509	-1.44684	-2.06877	-2.61409	-1.34997	-2.09293	-2.69246	-1.39645	-2.08245	-2.24403	-1.38589	-2.16026	-2.82334	-1.26132	-1.89428	-2.43707	-1.27555	-2.1467	-2.50988	-1.33098	-2.1404	-2.70834	-1.12474	-1.93332
H	-1.52542	-0.06579	0.66761	-1.6944	0.13811	0.71155	-1.70324	0.19998	0.77024	-1.67327	0.1554	0.71973	-1.439	0.05309	0.72302	-1.55019	0.29728	0.79101	-1.50491	0.26055	0.62121	-1.48444	0.20964	0.56958	-1.54065	0.37715	0.85199
H	-0.88945	1.91375	-1.56446	-0.74653	1.77203	-1.68355	-0.75609	1.76233	-1.67383	-0.74475	1.79542	-1.67387	-0.7524	1.9084	-1.59763	-0.60386	1.80824	-1.68385	-0.71693	1.9339	-1.80602	-0.71733	1.95996	-1.80352	-0.58968	1.81671	-1.66485
H	2.383	2.65552	-0.15777	2.5056	2.37319	-0.15634	2.5216	2.36306	-0.2078	2.52427	2.36287	-0.16701	2.58003	2.54413	-0.28575	2.76524	2.16765	-0.35454	2.73069	2.1697	-0.69624	2.71494	2.21	-0.64041	2.81116	2.11247	-0.40668
H	1.28099	2.831	-1.50914	1.40067	2.71439	-1.47042	1.40003	2.68637	-1.51208	1.41739	2.70795	-1.47835	1.44174	2.75768	-1.60111	1.61919	2.5598	-1.61792	1.56336	2.41668	-1.9768	1.56233	2.46679	-1.93214	1.65103	2.51182	-1.65481

H	1.55716	0.46295	-2.13575	1.50169	0.3619	-2.23583	1.47485	0.32011	-2.2343	1.49446	0.35073	-2.23293	1.55717	0.39657	-2.20136	1.463	0.21396	-2.30151	1.31959	-0.05028	-2.25346	1.3547	0.00325	-2.2612	1.42585	0.16043	-2.29684
H	3.13162	1.24317	-2.01807	3.12797	1.02385	-2.10278	3.10777	0.97157	-2.13376	3.12697	0.99926	-2.10939	3.19135	1.04796	-2.16489	3.15956	0.68147	-2.28863	3.01519	0.41553	-2.36772	3.04536	0.49293	-2.34289	3.13331	0.58583	-2.32184
H	0.66795	-0.85019	-0.34657	0.55469	-1.03276	-0.49721	0.53579	-1.02204	-0.46206	0.54244	-1.02365	-0.48695	0.68144	-0.88965	-0.33569	0.49523	-1.06134	-0.4516	0.39034	-0.9032	-0.2323	0.41188	-0.91328	-0.27013	0.46043	-1.04924	-0.411
H	1.28525	-1.16824	2.41776	1.48526	-1.41302	2.2626	1.43421	-1.38569	2.29416	1.43746	-1.4104	2.27371	1.49408	-1.1605	2.39676	1.55883	-1.45887	2.24484	1.06585	-0.93468	2.4978	1.08207	-0.9745	2.47341	1.48358	-1.44588	2.27949
H	2.6318	-1.87046	1.54917	2.44551	-2.35033	1.13645	2.44404	-2.31478	1.20519	2.43576	-2.33541	1.1708	2.69683	-1.98915	1.43143	2.3834	-2.49445	1.09672	2.3077	-1.91428	1.7519	2.32119	-1.93899	1.70315	2.35608	-2.48436	1.17045
H	1.15359	-3.31157	0.23339	0.5437	-3.51064	0.24031	0.58091	-3.50522	0.25569	0.55587	-3.50043	0.21593	1.03422	-3.38945	0.34312	0.35779	-3.54158	0.34587	0.6317	-3.33567	0.64674	0.63372	-3.334	0.57221	0.35588	-3.54587	0.35455
H	0.96957	-3.59695	1.96028	0.56569	-3.67601	1.99047	0.56356	-3.67076	2.00617	0.5609	-3.6936	1.96355	0.93237	-3.54629	2.08949	0.45034	-3.64053	2.09673	0.45033	-3.35027	2.39804	0.48555	-3.39942	2.32525	0.40078	-3.64332	2.10733
H	-2.48813	-2.12099	2.19208	-1.21797	-2.45357	3.37377	-2.52542	-1.56966	2.50809	-0.90255	-0.92966	2.70129	-2.38203	-1.78074	2.40196	-2.38575	-1.23193	2.71326	-1.20694	-0.78888	2.87156	-2.77616	-1.57506	2.51228	-2.4648	-1.25704	2.65067
H	-1.25937	-3.02775	3.08091	-0.83278	-0.88251	2.68773	-1.29167	-2.51825	3.34339	-2.51974	-1.62743	2.55968	-1.14865	-2.67592	3.296	-1.1726	-2.24126	3.50573	-2.78798	-1.43215	2.43677	-1.54662	-2.42717	3.446	-1.29145	-2.29264	3.47009
H	-1.00691	-1.32896	2.72375	-2.47575	-1.51821	2.56053	-0.89196	-0.93039	2.70768	-1.22605	-2.52929	3.34985	-0.82312	-1.03804	2.75649	-0.693	-0.72932	2.75002	-1.67816	-2.38375	3.42912	-1.22474	-0.80831	2.85278	-0.77239	-0.76897	2.76732
H	1.0786	3.11303	1.66519	-0.66166	2.27742	1.74039	-0.61598	2.33482	1.73728	-0.63208	2.30197	1.74674	1.34886	3.08633	1.57063	-0.29271	2.3984	1.7086	0.31423	3.82463	-0.11067	0.29005	3.82419	-0.05783	-0.1993	2.45586	1.7084
H	0.43083	4.53199	-0.29086	2.18165	3.42367	1.70784	1.05524	3.64548	2.92621	2.21631	3.43444	1.68695	-0.98447	4.0615	-0.16532	2.05701	1.95049	2.48013	-1.50658	2.80463	1.27348	-1.57147	2.78007	1.24664	2.15359	1.96161	2.44253
H	-1.25011	4.01018	-0.1185	0.97564	3.58428	2.97819	1.72596	2.04233	2.59802	1.02178	3.60218	2.96709	-0.15115	5.00967	1.0647	2.6296	3.29824	1.48679	-0.31273	2.31277	2.47584	-0.41531	2.24924	2.47027	2.73962	3.27832	1.41586
H	-0.48823	4.97513	1.14428	1.67419	1.99836	2.62513	2.24228	3.44041	1.64446	1.71131	2.0129	2.61177	0.71249	4.50841	-0.38555	1.51284	3.60035	2.81192	-0.61588	4.02914	2.1822	-0.70343	3.97392	2.21538	1.65642	3.62968	2.75658
H	-1.87801	2.32471	1.76611	0.56846	4.44408	-0.0463	0.61719	4.45143	-0.10614	0.59225	4.44914	-0.0669	-1.63895	2.4499	1.76914	-0.01321	4.79352	1.15926	2.26045	2.71598	1.97477	2.63573	3.71573	0.71345	0.1293	4.83287	1.1113
H	-1.00948	3.31921	2.93983	-0.55854	4.70536	1.28165	-0.48857	4.75176	1.23149	-0.51193	4.72915	1.27639	-0.68103	3.4045	2.90519	-0.69458	3.93232	-0.22158	1.73167	4.39956	1.91344	2.17132	2.67376	2.06469	-0.59969	3.96511	-0.24072
H	-0.59462	1.60968	2.74738	-1.08963	3.84008	-0.16141	-1.04997	3.8663	-0.18753	-1.07279	3.8579	-0.15152	-0.36587	1.6733	2.71684	1.01189	4.39642	-0.21665	2.67951	3.73658	0.59238	1.64736	4.3594	2.01406	1.11777	4.38633	-0.2762
H	-2.56873	1.06394	-2.91522	-3.09935	2.24383	-2.07154	-3.16324	2.33145	-1.90118	-3.10479	2.29336	-2.00443	-3.17045	2.22009	-1.76361	-2.92837	2.44805	-1.97274	-2.52719	1.11483	-3.0362	-4.2164	1.15545	-2.6395	-2.94467	2.59541	-1.77487
H	-3.33799	2.12517	-1.71927	-4.21943	1.016	-2.67767	-4.27852	1.12699	-2.56454	-4.2491	1.0918	-2.61899	-4.19298	0.96181	-2.47401	-4.16528	1.29651	-2.49534	-3.1011	2.41111	-1.96944	-2.50954	1.01119	-3.08181	-4.19224	1.48674	-2.36542
H	-4.27597	0.85769	-2.52469	-2.48617	0.87426	-3.00988	-2.55794	1.02647	-2.94001	-2.52536	0.93061	-2.98189	-2.48205	1.03946	-2.89543	-2.46585	1.01696	-2.90546	-4.23156	1.19887	-2.59028	-3.05578	2.36293	-2.07069	-2.50836	1.23316	-2.82471
H	-4.80912	-0.03136	-0.33598	-3.90862	1.67413	0.25072	-4.93773	0.59708	-0.2727	-3.96457	0.51953	0.82011	-4.742	0.24562	-0.2148	-3.6671	1.98974	0.3958	-4.79891	0.65396	-0.29023	-3.77596	0.57234	0.77758	-4.77807	1.03854	-0.0371
H	3.44654	1.03602	1.21185	4.59534	0.37166	-0.22053	4.59253	0.34327	-0.25783	4.14453	-0.97899	0.84782	4.30896	-0.65517	0.90975	4.13124	-1.21161	0.73755	3.58901	0.62158	0.84353	4.56675	0.06722	-0.45154	4.11562	-1.28444	0.71368
H	4.25428	-0.52248	1.02952	3.46173	0.64351	1.1021	3.4784	0.64679	1.07455	4.59373	0.34909	-0.22829	4.71124	0.55658	-0.30867	4.66141	-0.04681	-0.47871	4.09039	-1.07024	0.83585	3.56827	0.64473	0.88247	4.64977	-0.15388	-0.53232
H	4.6091	0.79543	-0.09273	4.14847	-0.96142	0.85074	4.15022	-0.96741	0.84225	3.46795	0.6303	1.09906	3.62134	0.96723	1.02894	3.64756	0.48026	0.87709	4.55872	-0.00194	-0.49103	4.09914	-1.03751	0.84522	3.67833	0.42153	0.83486
H	4.20756	-2.41617	-2.31233	4.8992	-1.60866	-1.5217	3.93233	-0.98739	-2.8885	4.87742	-1.63969	-1.52468	2.48608	-1.43415	-3.11215	2.10975	-1.68566	-3.26517	4.52261	-2.21159	-1.42003	4.58014	-2.13107	-1.41728	2.23722	-3.39956	-2.57773
H	4.08748	-0.69084	-2.70778	3.96812	-2.66209	-2.60475	4.86277	-1.66302	-1.52889	3.93439	-2.69828	-2.59222	2.81494	-3.02349	-2.39579	2.3841	-3.31835	-2.62921	3.39088	-3.38043	-2.12904	3.47724	-3.29956	-2.17093	0.89712	-2.39222	-2.00495
H	5.05394	-1.22134	-1.30938	3.98481	-0.90973	-2.88042	3.90861	-2.73329	-2.57468	3.95826	-0.94861	-2.88422	1.34791	-2.14045	-1.94156	1.00124	-2.37185	-2.05425	3.51114	-1.74653	-2.80986	3.58775	-1.65035	-2.81549	2.01342	-1.77078	-3.24317

Table S4. Experimental and calculated ^{13}C NMR chemical shifts of **3**.

No.	Experimental (δ_C, ppm)	Calculated (δ_C, ppm)		Corrected (δ_C, ppm)	
		1*R*,4*S*,11*S*,12*R* **-3**	1*R*,4*S*,11*R*,12*S*-**3**	1*R*,4*S*,11*S*,12*R* **-3**	1*R*,4*S*,11*R*,12*S* -**3**
1	77.8	79.2	81.8	78.7	80.0
2	125.1	122.8	123.9	123.2	122.9
3	141	140.2	141.7	140.9	140.9
4	74.4	76.1	76.6	75.6	74.7
5	44.7	47.7	45.8	46.6	43.4
6	23.8	28.3	28.3	26.8	25.6
7	128.9	125.2	129.9	125.6	128.9
8	133.6	137.5	134.9	138.1	134.1
9	37.5	40.0	42.7	38.8	40.3
10	26.1	27.1	31.0	25.6	28.4
11	73.4	75.1	74.7	74.5	72.8
12	74.7	76.4	77.4	75.9	75.6
13	31.6	32.7	34.3	31.3	31.7
14	29.9	32.1	34.2	30.7	31.6
15	39.8	30.9	43.7	29.4	41.3
16	17.2	19.5	18.5	17.9	15.7
17	17.2	17.2	17.8	15.5	14.9
18	29	28.8	30.2	27.3	27.6
19	15	17.8	17.3	16.2	14.5
20	15.6	20.3	16.6	18.7	13.8
12-OMe	48.7	49.0	48.7	48.0	46.4
**R^2**		**0.995**	**0.9985**		
**MAE**		**2.42**	**2.26**		
**CMAE**				**1.97**	**1.34**

Table S5. Experimental and calculated ^{1}H NMR chemical shifts of **3**.

No.	Experimental	Calculated (δ_H, ppm)		Corrected (δ_H, ppm)	
	(δ_H, ppm)	1*R*,4*S*,11*S*,12*R* **-3**	1*R*,4*S*,11*R*,12*S*-**3**	1*R*,4*S*,11*S*,12*R* **-3**	1*R*,4*S*,11*R*,12*S*-**3**
2	5.43	5.65	5.67	5.30	5.33
3	5.92	6.50	6.26	6.10	5.88
5a	1.88	1.56	1.83	1.45	1.80
5b	1.63	1.67	1.65	1.55	1.63
6a	2.21	2.30	2.43	2.14	2.35
6b	2.11	2.22	2.14	2.07	2.08
7	5.16	5.47	5.64	5.13	5.30
9a	2.14	2.07	2.10	1.92	2.05
9b	1.99	2.44	2.08	2.28	2.03
10a	1.84	1.90	1.80	1.76	1.77
10b	1.29	1.89	1.39	1.75	1.39
11	3.23	3.61	3.60	3.37	3.43
13a	1.81	1.71	1.88	1.59	1.84
13b	1.54	1.62	1.40	1.50	1.40
14a	1.62	1.89	1.63	1.76	1.62
14b	1.54	1.40	1.50	1.30	1.49
15	1.75	2.54	1.54	2.37	1.53
16	0.78	0.75	0.78	0.68	0.83
17	0.86	0.83	0.81	0.76	0.85
18	1.31	1.28	1.27	1.18	1.28
19	1.61	1.83	1.80	1.70	1.77
20	1.11	1.33	1.19	1.23	1.21
12-OMe	3.18	3.25	3.25	3.04	3.10
**R^2**		**0.9733**	**0.9946**		
**MAE**		**0.23**	**0.13**		
**CMAE**				**0.18**	**0.08**

Table S6. Conformational analysis of the optimized isomers of **3**.

Conformations	*G* (hartree)	*ΔG* (kcal/mol)	Boltzmann distributions (%)
1*R*,4*S*,11*S*,12*R*-**3-1**	0	0.494867135	49.5
1*R*,4*S*,11*S*,12*R*-**3-2**	0.409763638	0.247615499	24.8
1*R*,4*S*,11*S*,12*R*-**3-3**	0.71096815	0.148845145	14.9
1*R*,4*S*,11*S*,12*R*-**3-4**	1.386795774	0.047508257	4.8
1*R*,4*S*,11*S*,12*R*-**3-5**	1.630269421	0.031484192	3.1
1*R*,4*S*,11*S*,12*R*-**3-6**	2.022462796	0.016228409	1.6
1*R*,4*S*,11*S*,12*R*-**3-7**	2.13353196	0.013451363	1.3
1*R*,4*S*,11*R*,12*S*-**3-1**	-1047.41551	0	60.1
1*R*,4*S*,11*R*,12*S*-**3-2**	-1047.413996	0.950049232	12.1
1*R*,4*S*,11*R*,12*S*-**3-3**	-1047.413887	1.018447756	10.7
1*R*,4*S*,11*R*,12*S*-**3-4**	-1047.413553	1.228035896	7.5
1*R*,4*S*,11*R*,12*S*-**3-5**	-1047.413106	1.508532598	4.7
1*R*,4*S*,11*R*,12*S*-**3-6**	-1047.412733	1.742593604	3.2
1*R*,4*S*,11*R*,12*S*-**3-7**	-1047.412143	2.11282415	1.7

Table S7. The coordinates of the optimized conformers of 1*R*,4*S*,11*S*,12*R*-**3**.

	1*R*,4*S*,11*S*,12*R*-**3-1**			1*R*,4*S*,11*S*,12*R*-**3-2**			1*R*,4*S*,11*S*,12*R*-**3-3**			1*R*,4*S*,11*S*,12*R*-**3-4**			1*R*,4*S*,11*S*,12*R*-**3-5**			1*R*,4*S*,11*S*,12*R*-**3-6**			1*R*,4*S*,11*S*,12*R*-**3-7**		
	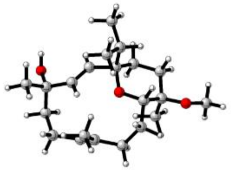			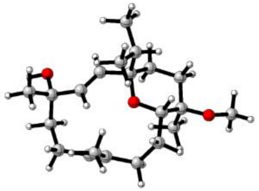			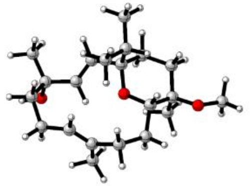			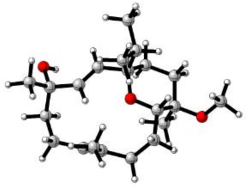			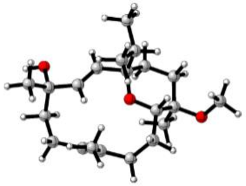			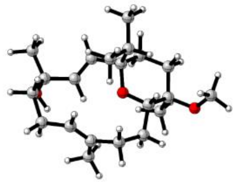			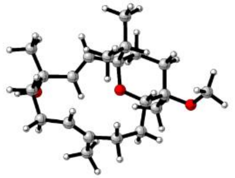		
C	-1.56091	-2.4173	-0.4557	-1.57416	-2.43732	-0.42365	-2.05572	-1.71944	1.24963	-1.73227	-2.39115	-0.38101	-1.71602	-2.37785	-0.41752	-2.01178	-1.69488	1.30423	-1.99058	-1.69684	1.30223
C	-3.04748	-2.19281	-0.56552	-3.05915	-2.19695	-0.52064	-3.42058	-1.60974	0.61821	-3.20301	-2.06229	-0.336	-3.19049	-2.0654	-0.39148	-3.41839	-1.52684	0.78665	-3.3932	-1.53914	0.77177
C	-3.4631	-0.94755	-1.38053	-3.46001	-0.96112	-1.36058	-3.90406	-0.16967	0.34042	-3.60943	-0.8201	-1.16418	-3.59593	-0.79956	-1.18281	-3.86738	-0.06761	0.54409	-3.85297	-0.08223	0.54676
C	-3.3884	0.41714	-0.66095	-3.37356	0.43016	-0.67884	-3.31379	0.52351	-0.919	-3.38342	0.57724	-0.52567	-3.38308	0.57525	-0.49558	-3.33494	0.62202	-0.74262	-3.33524	0.61044	-0.7445
C	-2.00299	0.62548	-0.12067	-1.99093	0.63069	-0.14611	-1.82036	0.54856	-0.78984	-1.9553	0.68799	-0.0784	-1.95618	0.69471	-0.06552	-1.8343	0.61096	-0.699	-1.83618	0.59154	-0.7229
C	-1.0815	1.46997	-0.56892	-1.07802	1.50442	-0.5515	-1.04795	1.57274	-0.44396	-1.033	1.54717	-0.49565	-1.03144	1.52625	-0.5286	-0.99713	1.60935	-0.43944	-1.01216	1.58769	-0.41688
C	0.34657	1.43309	-0.06443	0.35084	1.4448	-0.0527	0.39331	1.38212	-0.02274	0.4247	1.39658	-0.11235	0.42452	1.388	-0.13516	0.46088	1.35864	-0.11563	0.4504	1.35041	-0.10664
C	1.30514	1.70186	-1.23906	1.30638	1.71735	-1.22955	1.39844	1.80887	-1.10762	1.30014	1.65857	-1.35471	1.30685	1.63527	-1.37487	1.40764	1.76498	-1.26102	1.38222	1.75981	-1.2635
C	2.73899	1.26324	-0.93331	2.73814	1.26187	-0.93845	2.81405	1.31389	-0.78123	2.72589	1.129	-1.18	2.7351	1.1201	-1.17971	2.82128	1.21359	-1.02858	2.80252	1.22058	-1.04415
C	2.80084	-0.21344	-0.50518	2.7883	-0.21821	-0.52111	2.85633	-0.21143	-0.55042	2.7228	-0.36313	-0.81369	2.74019	-0.36701	-0.79425	2.80988	-0.31527	-0.83568	2.80382	-0.30755	-0.84583
C	1.82855	-0.38787	0.68823	1.82551	-0.38783	0.68035	1.78632	-0.5689	0.51084	1.85445	-0.52797	0.46165	1.85695	-0.52633	0.47196	1.81117	-0.65689	0.30284	1.82089	-0.65097	0.30557
C	1.67238	-1.81408	1.23469	1.67291	-1.81214	1.23334	1.57506	-2.06632	0.74386	1.68232	-1.96218	0.98098	1.67858	-1.95885	0.99343	1.55959	-2.14943	0.52112	1.58334	-2.14405	0.53626
C	-0.79273	-2.47863	0.63784	-0.79427	-2.48247	0.66235	-0.99718	-2.4151	0.8206	-0.85661	-2.49105	0.6253	-0.85694	-2.48682	0.60197	-1.01209	-2.41509	0.78371	-0.98653	-2.41647	0.7899
C	0.68951	-2.77619	0.52557	0.68663	-2.78065	0.53828	0.31902	-2.41157	1.57783	0.5885	-2.85902	0.35476	0.59208	-2.85428	0.35237	0.35649	-2.46897	1.43935	0.37891	-2.46396	1.45235
C	-1.30455	-2.28992	2.04091	-1.29092	-2.26905	2.06748	-1.0072	-3.24597	-0.43816	-1.20691	-2.2722	2.07299	-1.22992	-2.28261	2.046	-1.14461	-3.21983	-0.48516	-1.10982	-3.22709	-0.47582
C	0.58032	2.4192	1.12177	0.59706	2.41647	1.14278	0.63679	2.10718	1.34014	0.80975	2.31725	1.08726	0.80104	2.3266	1.05356	0.81838	2.05491	1.23746	0.81664	2.05504	1.2396
C	0.1425	3.84906	0.78251	0.16501	3.8526	0.82241	0.56477	3.63585	1.24511	0.44096	3.78499	0.83913	0.43108	3.78944	0.78009	0.80172	3.58631	1.1654	0.79585	3.58596	1.16014
C	-0.11303	1.94664	2.40212	-0.09485	1.93188	2.41951	-0.30679	1.58655	2.42765	0.18214	1.8317	2.39647	0.16752	1.86095	2.36713	-0.07474	1.55571	2.37662	-0.06645	1.55869	2.38783
C	-4.41842	0.51092	0.47371	-4.40383	0.56646	0.4514	-3.94776	1.90208	-1.10107	-4.33227	0.81407	0.64949	-4.31723	0.74953	0.71038	-3.94686	2.00909	-0.88051	-3.9403	2.00857	-0.86482
O	-3.71968	1.37406	-1.68047	-3.62546	1.4376	-1.67073	-3.58374	-0.28754	-2.08414	-3.72954	1.57949	-1.49397	-3.65255	1.60904	-1.455	-3.79198	-0.11405	-1.90243	-3.71119	-0.17658	-1.89651
C	2.49064	-1.13649	-1.68556	2.45279	-1.13117	-1.70257	2.62737	-0.96452	-1.86539	2.22474	-1.20173	-1.98743	2.2696	-1.22359	-1.96616	2.43091	-1.01005	-2.1431	2.41312	-1.00965	-2.1458
O	4.08007	-0.51819	0.07949	4.07042	-0.54141	0.04714	4.08962	-0.59917	0.07867	4.05055	-0.87041	-0.56817	4.06901	-0.85974	-0.52182	4.1163	-0.83997	-0.52866	4.11792	-0.82196	-0.55283
O	0.52811	0.07258	0.37429	0.52566	0.07834	0.37304	0.52801	-0.03995	0.14164	0.55676	0.01099	0.2618	0.55998	0.00786	0.25979	0.55004	-0.06978	0.02727	0.55203	-0.07611	0.04143
C	5.20778	-0.46535	-0.7755	5.18915	-0.49092	-0.81952	5.27411	-0.43173	-0.67965	4.9278	-0.11659	0.25254	4.93081	-0.08253	0.29297	4.87435	-0.21098	0.49128	4.88208	-0.18589	0.45775
H	-1.06258	-2.53308	-1.41881	-1.08757	-2.57162	-1.39034	-1.9321	-1.12457	2.15396	-1.34718	-2.54055	-1.39041	-1.31414	-2.51248	-1.42217	-1.79995	-1.12174	2.20598	-1.7855	-1.11967	2.20331
H	-3.48049	-3.06	-1.07988	-3.51147	-3.06932	-1.00905	-3.4769	-2.17223	-0.31455	-3.7553	-2.91325	-0.75416	-3.72278	-2.90777	-0.85116	-3.58591	-2.10633	-0.1243	-3.53257	-2.08654	-0.16131

H	-3.52436	-2.17252	0.41657	-3.524	-2.14355	0.4659	-4.15517	-2.06551	1.29543	-3.5622	-1.95901	0.69004	-3.5717	-2.00434	0.62996	-4.11111	-1.94765	1.52681	-4.09042	-1.9835	1.49455
H	-2.83952	-0.87176	-2.27644	-2.83618	-0.91282	-2.25819	-4.99474	-0.19144	0.21417	-3.06138	-0.84042	-2.11441	-3.04618	-0.76905	-2.1283	-4.96044	-0.05237	0.47802	-4.9501	-0.07078	0.5014
H	-4.49514	-1.07014	-1.72508	-4.49498	-1.09048	-1.70275	-3.70839	0.46539	1.21054	-4.67234	-0.88245	-1.418	-4.66063	-0.87239	-1.43988	-3.58996	0.54622	1.40762	-3.57524	0.53549	1.40699
H	-1.72164	-0.05242	0.67082	-1.69934	-0.08978	0.60145	-1.36531	-0.42736	-0.86131	-1.63728	-0.08897	0.59847	-1.64841	-0.04995	0.65129	-1.40461	-0.37995	-0.75411	-1.41601	-0.39518	-0.84336
H	-1.30112	2.15884	-1.37928	-1.3112	2.24631	-1.30778	-1.44857	2.57671	-0.35219	-1.28064	2.35922	-1.17314	-1.28041	2.28872	-1.25874	-1.34576	2.6328	-0.34931	-1.37638	2.60033	-0.27979
H	0.93402	1.15581	-2.11073	0.92464	1.18508	-2.10513	1.06345	1.37738	-2.05407	0.82717	1.17116	-2.21099	0.84337	1.13034	-2.22599	0.99562	1.36321	-2.18933	0.96324	1.35056	-2.18527
H	1.28304	2.75967	-1.51053	1.29269	2.77835	-1.48869	1.39455	2.89195	-1.24522	1.3195	2.72651	-1.58326	1.31961	2.69927	-1.62143	1.43545	2.84973	-1.3809	1.3997	2.84432	-1.38828
H	3.16883	1.8679	-0.12906	3.18041	1.85618	-0.13314	3.1978	1.80483	0.11818	3.23164	1.70208	-0.39917	3.22868	1.70755	-0.40183	3.25633	1.69733	-0.15072	3.2431	1.7106	-0.17243
H	3.36042	1.42817	-1.81751	3.35417	1.42701	-1.82646	3.48716	1.58685	-1.59836	3.30214	1.27604	-2.0985	3.31937	1.25958	-2.09437	3.47168	1.4606	-1.87297	3.44229	1.46929	-1.89624
H	2.26739	0.22104	1.49126	2.27226	0.22111	1.47891	2.12359	-0.12162	1.45503	2.36312	0.0366	1.25479	2.35806	0.04137	1.26807	2.20248	-0.24432	1.24141	2.21983	-0.22991	1.23727
H	2.66618	-2.26836	1.23793	2.66734	-2.26487	1.23103	1.52051	-2.59026	-0.21119	2.64814	-2.46061	0.86223	2.64542	-2.45873	0.88972	1.41955	-2.65045	-0.43729	1.45054	-2.65553	-0.41755
H	1.37989	-1.7349	2.28579	1.38866	-1.72833	2.28632	2.46553	-2.44767	1.24848	1.50707	-1.9085	2.05939	1.48766	-1.9022	2.06912	2.46857	-2.57581	0.95269	2.49424	-2.55878	0.97535
H	0.95045	-2.8906	-0.52554	0.93873	-2.9014	-0.51441	0.4747	-3.4212	1.98199	0.73626	-2.95612	-0.71993	0.75463	-2.9512	-0.72	0.5059	-3.49079	1.81364	0.531	-3.48379	1.83142
H	0.86162	-3.76063	0.98416	0.8642	-3.7616	1.00224	0.24993	-1.74516	2.44259	0.75825	-3.86171	0.77269	0.75464	-3.85754	0.772	0.37344	-1.8177	2.31823	0.39098	-1.8086	2.3284
H	-0.94857	-1.34075	2.455	-2.37769	-2.27996	2.14536	-0.52145	-2.71565	-1.26517	-2.2806	-2.22829	2.25279	-0.79688	-1.35157	2.42605	-0.69864	-2.69214	-1.33667	-0.57766	-4.1798	-0.38543
H	-0.932	-3.0786	2.70387	-0.94049	-1.30751	2.45661	-2.01279	-3.5044	-0.76709	-0.77148	-1.33582	2.43643	-0.83344	-3.09014	2.6713	-2.18047	-3.43995	-0.74154	-0.66472	-2.69968	-1.32719
H	-2.3923	-2.29023	2.10531	-0.90167	-3.03977	2.74176	-0.45276	-4.17959	-0.29674	-0.79763	-3.07209	2.69974	-2.3062	-2.23599	2.20938	-0.61635	-4.17502	-0.40249	-2.14346	-3.44853	-0.73777
H	1.65729	2.4437	1.31989	1.67514	2.43322	1.33652	1.65605	1.8648	1.65272	1.898	2.26899	1.20385	1.88864	2.28187	1.17796	1.84497	1.7684	1.4821	1.84574	1.77264	1.47822
H	0.38331	4.5232	1.60871	0.64965	4.24908	-0.07247	1.30821	4.04773	0.56019	0.79778	4.40656	1.66456	-0.65198	3.91039	0.69544	-0.1861	3.97687	0.90771	1.50988	3.9766	0.4328
H	0.63496	4.24065	-0.11015	-0.91614	3.91233	0.6724	-0.41945	3.98435	0.92119	0.88012	4.18132	-0.07891	0.77168	4.42395	1.6027	1.06692	4.00364	2.14043	-0.19294	3.97304	0.90049
H	-0.93743	3.89925	0.61908	0.42144	4.51673	1.65213	0.75271	4.07511	2.22849	-0.64276	3.91227	0.77504	0.88367	4.17469	-0.13611	1.5172	3.97898	0.44059	1.06038	4.00922	2.13289
H	-1.19731	1.90825	2.27051	0.24661	0.93908	2.71351	-1.34312	1.86982	2.22586	0.48158	2.48237	3.22268	0.4633	0.84276	2.62136	-1.10767	1.88924	2.2489	-1.10039	1.89275	2.26896
H	0.09994	2.63726	3.22271	-1.17831	1.88563	2.2838	-0.02986	1.99873	3.40192	0.48721	0.81367	2.63972	-0.92324	1.88543	2.30547	0.28424	1.93643	3.33676	0.30094	1.94079	3.34433
H	0.22153	0.95284	2.70053	0.11089	2.61958	3.24446	-0.27143	0.49996	2.49092	-0.90892	1.84692	2.33616	0.47191	2.51801	3.18656	-0.08572	0.46753	2.41965	-0.07783	0.47058	2.43203
H	-4.21	-0.20179	1.27439	-4.36971	1.57957	0.85607	-5.02879	1.80607	-1.23904	-4.13088	0.12064	1.46753	-4.10548	0.02472	1.49934	-5.03098	1.92554	-0.97997	-5.03076	1.94423	-0.92473
H	-4.40163	1.51148	0.91613	-5.41471	0.3755	0.07621	-3.78278	2.54225	-0.23127	-4.20455	1.83099	1.02515	-4.19484	1.75144	1.12582	-3.73018	2.6262	-0.00646	-3.69469	2.63256	-0.00237
H	-5.42246	0.32001	0.08787	-4.21096	-0.13323	1.2673	-3.53292	2.39561	-1.98193	-5.3696	0.68707	0.33048	-5.36229	0.62307	0.40893	-3.558	2.51001	-1.76913	-3.57622	2.50182	-1.76801
H	-3.69696	2.25073	-1.27677	-4.50514	1.27922	-2.0331	-4.53945	-0.41285	-2.13899	-3.15445	1.45564	-2.25957	-4.56496	1.5051	-1.74986	-3.23657	-0.8964	-1.99786	-4.67267	-0.26405	-1.89135
H	1.4261	-1.11734	-1.91744	1.38427	-1.10414	-1.91477	2.93731	-2.0066	-1.77601	1.1488	-1.10165	-2.12262	1.19624	-1.1312	-2.12451	2.6934	-2.0669	-2.0955	2.68679	-2.06371	-2.09898
H	2.77393	-2.16449	-1.45674	2.73406	-2.16223	-1.48512	3.18534	-0.51428	-2.68927	2.46004	-2.25251	-1.82004	2.50752	-2.27087	-1.78127	2.97927	-0.56741	-2.97751	2.94486	-0.56418	-2.98951
H	3.03148	-0.8283	-2.58347	2.97952	-0.82037	-2.60797	1.56983	-0.94661	-2.13161	2.72608	-0.89045	-2.90704	2.78741	-0.92058	-2.87936	1.36292	-0.926	-2.3436	1.34157	-0.93712	-2.33074
H	6.07246	-0.69847	-0.15418	5.11279	-1.2154	-1.63752	5.4504	0.61537	-0.94947	5.79449	-0.75406	0.42839	5.2726	0.82551	-0.21468	5.23768	0.7767	0.1887	4.34071	-0.08984	1.40499
H	5.15	-1.20219	-1.58385	5.34139	0.50695	-1.24521	6.09376	-0.76889	-0.04516	5.26648	0.80184	-0.23806	4.47738	0.19864	1.24936	4.32249	-0.10983	1.43186	5.75101	-0.82317	0.62311
H	5.35196	0.52837	-1.21352	6.05768	-0.74398	-0.21145	5.26897	-1.0353	-1.59376	4.48946	0.14316	1.22189	5.79771	-0.71143	0.49685	5.73501	-0.85657	0.66699	5.23246	0.80531	0.15115

Table S8. The coordinates of the optimized conformers of 1*R*,4*S*,11*R*,12*S*-**3**.

	1*R*,4*S*,11*R*,12*S*-**3-1**			1*R*,4*S*,11*R*,12*S*-**3-2**			1*R*,4*S*,11*R*,12*S*-**3-3**			1*R*,4*S*,11*R*,12*S*-**3-4**			1*R*,4*S*,11*R*,12*S*-**3-5**			1*R*,4*S*,11*R*,12*S*-**3-6**			1*R*,4*S*,11*R*,12*S*-**3-7**		
	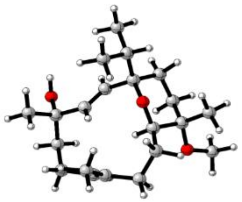			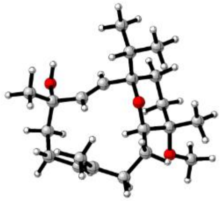			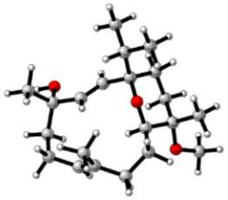			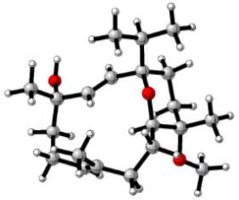			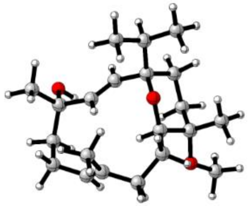			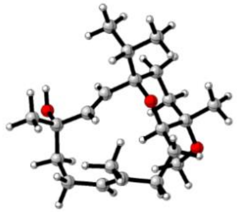			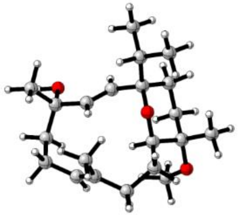		
C	-1.24857	-2.67961	-0.23549	-1.81671	-2.45574	-0.06506	-1.80416	-2.54165	0.09248	-1.72543	-2.39727	0.06549	-1.783	-2.44314	0.25845	-2.00848	-2.27569	0.14006	-1.97301	-2.33616	0.24449
C	-2.66961	-2.47319	-0.67779	-3.20818	-1.99858	-0.39914	-3.21096	-2.08157	-0.1651	-3.10364	-2.00991	-0.39106	-3.17141	-2.01451	-0.12504	-3.35408	-1.65896	-0.11673	-3.32977	-1.72817	0.02884
C	-2.87168	-1.25342	-1.59735	-3.25501	-0.81801	-1.38812	-3.30577	-1.03714	-1.2976	-3.12688	-0.88607	-1.44573	-3.20705	-1.05668	-1.33569	-3.3341	-0.55507	-1.19253	-3.35542	-0.69978	-1.12228
C	-2.92757	0.13689	-0.93189	-3.01451	0.59279	-0.81537	-3.04564	0.44098	-0.92387	-2.93287	0.56217	-0.95012	-2.94364	0.44686	-1.07668	-2.90416	0.85419	-0.74095	-2.92902	0.74979	-0.79065
C	-1.64657	0.49318	-0.21511	-1.66577	0.73653	-0.14971	-1.72528	0.63314	-0.23206	-1.58334	0.78118	-0.30821	-1.61395	0.68144	-0.40285	-1.52805	0.87793	-0.1195	-1.5655	0.81504	-0.16215
C	-0.75057	1.3796	-0.63626	-0.63402	1.413	-0.64282	-0.70137	1.34169	-0.69149	-0.57894	1.47304	-0.83483	-0.6343	1.45294	-0.85763	-0.44486	1.42378	-0.66134	-0.49726	1.40864	-0.68003
C	0.49434	1.78136	0.13201	0.67548	1.62822	0.10011	0.56446	1.59726	0.11186	0.75639	1.7317	-0.15558	0.67492	1.71722	-0.13354	0.894	1.52826	0.05415	0.82641	1.54044	0.05755
C	1.65703	2.08065	-0.83454	1.83947	1.78269	-0.89425	1.74671	1.88965	-0.82685	1.88188	1.66566	-1.20846	1.82318	1.75001	-1.16246	2.04451	1.60156	-0.96583	1.98527	1.6818	-0.94534
C	2.25109	0.81087	-1.44984	2.21013	0.45288	-1.5561	2.23301	0.62827	-1.54378	2.15263	0.23349	-1.6758	2.16438	0.35359	-1.68637	2.27957	0.25356	-1.65198	2.27713	0.35951	-1.65854
C	2.62692	-0.22016	-0.37339	2.47255	-0.64112	-0.50865	2.55201	-0.49242	-0.54306	2.41383	-0.69987	-0.48089	2.46471	-0.60935	-0.52609	2.48721	-0.86133	-0.61803	2.52576	-0.76534	-0.64542
C	1.35225	-0.46979	0.45472	1.20777	-0.7294	0.36786	1.27674	-0.73145	0.29061	1.18553	-0.58106	0.44198	1.22399	-0.60461	0.38815	1.24203	-0.87239	0.29742	1.27702	-0.85303	0.26162
C	1.47033	-1.51966	1.56325	1.23816	-1.79805	1.46549	1.40365	-1.82821	1.35435	1.17949	-1.4819	1.679	1.30188	-1.52724	1.60999	1.24766	-1.94475	1.39048	1.34573	-1.93725	1.34145
C	-0.73741	-2.66986	1.00089	-1.19687	-2.49081	1.11976	-1.0748	-2.46476	1.21071	-1.19179	-2.3362	1.29112	-1.13289	-2.27535	1.41556	-1.28568	-2.30216	1.26529	-1.19042	-2.30742	1.32869
C	0.75126	-2.83864	1.21011	0.242	-2.94707	1.20904	0.35597	-2.94957	1.21014	0.25832	-2.70976	1.50938	0.31006	-2.70755	1.5422	0.08107	-2.94719	1.2712	0.17858	-2.94422	1.28614
C	-1.5389	-2.42426	2.25196	-1.81003	-2.03584	2.41758	-1.54891	-1.85984	2.50495	-1.91614	-1.83169	2.51125	-1.71154	-1.59271	2.62689	-1.7028	-1.66766	2.56504	-1.53782	-1.61567	2.61956
C	0.21604	3.01602	1.0382	0.52832	2.85853	1.04209	0.30053	2.75813	1.11473	0.72488	3.12194	0.55383	0.57115	3.06624	0.64591	0.86525	2.75831	1.00713	0.7405	2.74441	1.03985
C	-0.20677	4.25291	0.23859	1.74634	3.07985	1.94484	1.46728	3.01118	2.07486	-0.40206	3.21875	1.58911	-0.5722	3.05545	1.66771	2.11636	2.87625	1.88348	1.97753	2.89058	1.93193
C	-0.80496	2.71921	2.13903	0.17865	4.14203	0.28275	-0.11308	4.05466	0.41159	2.057	3.48014	1.22852	1.87653	3.45181	1.35639	0.60097	4.07052	0.26247	0.42989	4.06287	0.32453
C	-4.1006	0.24075	0.0545	-4.11122	0.98307	0.18723	-4.16373	0.9888	-0.02188	-4.03447	0.96828	0.04073	-4.05563	1.06649	-0.22676	-3.90987	1.44963	0.25683	-3.9418	1.42473	0.14835
O	-3.16723	1.02364	-2.03862	-3.11531	1.44172	-1.97215	-3.01385	1.20504	-2.14084	-3.07143	1.34298	-2.14977	-3.02373	1.12768	-2.33957	-2.94128	1.62195	-1.95568	-2.87475	1.4862	-2.02371
C	3.79875	0.25717	0.48781	3.73327	-0.35094	0.30977	3.75948	-0.14268	0.33136	3.72278	-0.3602	0.23576	3.74396	-0.21966	0.21952	3.78031	-0.6559	0.16581	3.80246	-0.518	0.1536
O	2.88689	-1.50513	-0.96515	2.523	-1.93866	-1.12766	2.72839	-1.74836	-1.22229	2.37594	-2.07808	-0.89275	2.51038	-1.97041	-0.99047	2.64592	-2.15693	-1.23023	2.74989	-2.03891	-1.28324
O	0.90227	0.75191	1.0538	0.94233	0.53089	0.99565	0.87623	0.47038	0.95902	1.0152	0.77273	0.88457	0.94495	0.71855	0.86664	1.0888	0.40509	0.93787	1.05263	0.40434	0.91974
C	4.02455	-1.60546	-1.80215	3.60849	-2.19002	-2.0014	3.86173	-1.86924	-2.06197	3.39454	-2.51066	-1.776	3.57904	-2.31621	-1.85268	1.73344	-2.53463	-2.24863	1.84769	-2.45	-2.29776
H	-0.55191	-2.84773	-1.05709	-1.24728	-2.79463	-0.93085	-1.32386	-2.9836	-0.78082	-1.08247	-2.76219	-0.73585	-1.23805	-2.93536	-0.54764	-1.5776	-2.76082	-0.73653	-1.58607	-2.85824	-0.63143

H	-2.97609	-3.35528	-1.25342	-3.73266	-2.8345	-0.87817	-3.80388	-2.95123	-0.47372	-3.56475	-2.88846	-0.85906	-3.74041	-2.90849	-0.40824	-4.0235	-2.44804	-0.48	-4.02619	-2.53255	-0.23713
H	-3.35607	-2.42538	0.16943	-3.78673	-1.76707	0.49706	-3.69028	-1.70638	0.74121	-3.75222	-1.75294	0.44827	-3.71103	-1.57778	0.71733	-3.8127	-1.28786	0.80166	-3.72787	-1.28619	0.94416
H	-3.81408	-1.36896	-2.14184	-4.23703	-0.79034	-1.87049	-4.30829	-1.08318	-1.74146	-4.09154	-0.90321	-1.96246	-4.18772	-1.11583	-1.81733	-4.33427	-0.44982	-1.62384	-4.37266	-0.64774	-1.53
H	-2.07737	-1.22333	-2.34963	-2.52029	-0.97596	-2.18364	-2.59989	-1.29683	-2.09224	-2.3621	-1.07683	-2.20502	-2.47584	-1.40209	-2.0774	-2.67091	-0.85137	-2.01114	-2.71066	-1.04664	-1.93556
H	-1.47847	-0.01535	0.72679	-1.5652	0.26333	0.82083	-1.64385	0.16494	0.74113	-1.45976	0.34501	0.67609	-1.48169	0.17961	0.54665	-1.45018	0.43711	0.86783	-1.48686	0.35352	0.81436
H	-0.90601	1.8791	-1.58815	-0.71873	1.8732	-1.62286	-0.77598	1.81546	-1.66424	-0.69178	1.90675	-1.82518	-0.76299	1.98797	-1.79518	-0.50473	1.85569	-1.65593	-0.56994	1.87565	-1.6562
H	2.43255	2.62359	-0.28607	2.711	2.18358	-0.37377	2.56988	2.3223	-0.25561	2.79944	2.07653	-0.78909	2.71399	2.18107	-0.7074	2.95798	1.92328	-0.46433	2.88107	2.026	-0.42711
H	1.31548	2.74376	-1.63259	1.56999	2.51359	-1.66098	1.4484	2.64086	-1.56243	1.61689	2.2983	-2.06087	1.5455	2.40942	-1.99006	1.81511	2.36215	-1.71659	1.73422	2.45029	-1.68077
H	1.52339	0.34105	-2.11978	1.39356	0.10847	-2.19911	1.46097	0.25748	-2.226	1.29111	-0.16048	-2.22444	1.32566	-0.05929	-2.25619	1.40946	0.01712	-2.27101	1.41939	0.10104	-2.2861
H	3.12182	1.07766	-2.05514	3.08576	0.59754	-2.19486	3.1111	0.8694	-2.14929	3.00324	0.23272	-2.36292	3.01797	0.41943	-2.3666	3.1447	0.30648	-2.3202	3.14257	0.45955	-2.32101
H	0.59833	-0.80324	-0.26026	0.38875	-0.95388	-0.31918	0.49882	-1.01154	-0.42596	0.33268	-0.84995	-0.18099	0.39736	-0.92981	-0.24661	0.37002	-1.02498	-0.34314	0.41637	-1.04509	-0.38468
H	1.04798	-1.08829	2.47388	1.01771	-1.30577	2.41553	1.33151	-1.35441	2.33626	0.84909	-0.87499	2.52541	1.11239	-0.91948	2.49763	1.21902	-1.43794	2.35773	1.38008	-1.44136	2.31423
H	2.51596	-1.74237	1.78264	2.23626	-2.22777	1.56246	2.38944	-2.29186	1.30143	2.18602	-1.82827	1.92138	2.30689	-1.93621	1.72298	2.17825	-2.51305	1.36183	2.27247	-2.50436	1.24493
H	1.21013	-3.24655	0.30685	0.5286	-3.44758	0.28084	0.55628	-3.48864	0.27989	0.62372	-3.2959	0.66401	0.5821	-3.33578	0.69036	0.21306	-3.53004	0.35507	0.27319	-3.53924	0.3729
H	0.93166	-3.56013	2.01644	0.34703	-3.68396	2.01514	0.50485	-3.66558	2.02875	0.3485	-3.33928	2.40295	0.43837	-3.31826	2.44465	0.15242	-3.65721	2.10453	0.28993	-3.6427	2.12513
H	-2.60939	-2.33953	2.06827	-2.85776	-1.75217	2.32312	-2.60572	-1.59421	2.49544	-1.88391	-2.5758	3.31456	-1.18617	-0.65453	2.83598	-2.71443	-1.2635	2.54186	-1.41015	-2.29608	3.4684
H	-1.38743	-3.23479	2.97314	-1.74503	-2.82555	3.17406	-1.3867	-2.55255	3.33795	-1.43699	-0.9297	2.90779	-2.77117	-1.36091	2.5247	-1.65213	-2.39412	3.38331	-0.86553	-0.76927	2.79439
H	-1.21619	-1.5038	2.75091	-1.26963	-1.17392	2.82383	-0.97829	-0.95448	2.73646	-2.96204	-1.59169	2.32341	-1.59138	-2.2198	3.51685	-1.02399	-0.85102	2.83279	-2.56058	-1.24044	2.64592
H	1.17605	3.23061	1.52329	-0.32074	2.60466	1.68727	-0.55099	2.41128	1.71167	0.529	3.85722	-0.23707	0.34892	3.83128	-0.10873	0.01292	2.56742	1.66948	-0.10992	2.50346	1.68818
H	-1.15526	4.08355	-0.27931	2.61394	3.438	1.38363	2.32878	3.45393	1.56696	-0.39867	4.20634	2.05769	-0.40084	2.2874	2.42526	2.99682	3.16947	1.30491	1.78646	3.63192	2.71317
H	-0.34963	5.10285	0.91088	1.51663	3.8384	2.69859	1.16016	3.71336	2.85536	-1.38452	3.06048	1.14515	-0.63182	4.02191	2.17505	1.96384	3.64465	2.64675	2.23068	1.94643	2.41345
H	0.5359	4.54663	-0.50573	2.02645	2.16171	2.46025	1.79007	2.08824	2.55576	-0.26454	2.47254	2.37474	-1.53777	2.85981	1.20238	2.3349	1.93505	2.38695	2.85032	3.23514	1.37016
H	-0.52023	1.8386	2.71429	0.00241	4.95981	0.98632	-0.37771	4.81596	1.15019	2.87789	3.60126	0.52048	2.19123	2.65692	2.0356	1.42506	4.32322	-0.41084	-0.47941	3.99774	-0.27631
H	-1.79997	2.54565	1.7218	-0.7224	4.0274	-0.32349	-0.97716	3.90836	-0.23955	2.33862	2.70991	1.94919	1.72444	4.35773	1.94886	0.49946	4.8939	0.97416	1.24982	4.36509	-0.3335
H	-0.87406	3.56932	2.82359	0.99295	4.45249	-0.37821	0.70263	4.46018	-0.19409	1.95877	4.42533	1.76871	2.69665	3.65615	0.66664	-0.31654	4.02834	-0.32851	0.28934	4.86353	1.05548
H	-5.0335	-0.04363	-0.43751	-4.07696	0.37064	1.09051	-4.19774	0.4808	0.9443	-3.95889	0.42346	0.98377	-4.04673	0.67236	0.79053	-4.91491	1.43609	-0.17074	-3.97465	0.94577	1.12916
H	-3.95738	-0.39624	0.92949	-3.98561	2.0251	0.49573	-4.00082	2.05286	0.15785	-3.95549	2.03407	0.27443	-3.91381	2.1473	-0.17372	-3.9238	0.90208	1.20144	-3.66718	2.47134	0.28972
H	-4.19853	1.26931	0.41386	-5.09629	0.87457	-0.27222	-5.13922	0.86219	-0.50267	-5.01952	0.78227	-0.39332	-5.03237	0.86343	-0.67259	-3.64401	2.48578	0.48558	-4.94846	1.38315	-0.28022
H	-3.20803	1.92274	-1.68876	-2.98273	2.35219	-1.67907	-3.85085	1.06113	-2.59767	-2.96401	2.27165	-1.90831	-2.3626	0.73801	-2.92547	-2.70924	2.53271	-1.73401	-3.74686	1.4361	-2.43245
H	4.21594	-0.56786	1.06755	3.54295	0.45103	1.02315	3.48316	0.60754	1.07219	3.60147	0.54217	0.83522	3.56082	0.64632	0.85571	4.01055	-1.5426	0.75741	4.63177	-0.29658	-0.52237
H	4.60099	0.68448	-0.1182	4.0453	-1.23353	0.87039	4.12311	-1.02271	0.86416	4.01929	-1.1713	0.9029	4.08704	-1.03718	0.85543	4.61311	-0.48746	-0.52112	3.68259	0.3134	0.84682
H	3.46124	1.02353	1.18587	4.56661	-0.04243	-0.32576	4.58498	0.25991	-0.26027	4.53905	-0.18677	-0.46937	4.55326	0.03542	-0.46861	3.70448	0.1947	0.84184	4.06856	-1.4056	0.72845
H	4.05601	-2.63664	-2.15381	4.57419	-2.13748	-1.48684	4.80083	-1.77303	-1.50606	3.37348	-1.97305	-2.73024	3.44268	-3.36837	-2.10241	0.68754	-2.41213	-1.94956	2.09709	-3.48817	-2.51847
H	3.96049	-0.94275	-2.67216	3.46985	-3.20347	-2.37784	3.81568	-2.86805	-2.49607	4.39298	-2.41045	-1.3365	3.57006	-1.73395	-2.78071	1.89925	-1.9809	-3.17856	0.80097	-2.40603	-1.97974
H	4.95414	-1.3914	-1.26353	3.62498	-1.5015	-2.85338	3.85737	-1.13575	-2.87582	3.20407	-3.56635	-1.96897	4.55511	-2.19521	-1.37038	1.91566	-3.59275	-2.43796	1.96025	-1.86259	-3.21493

Table S9. Conformational analysis of the optimized isomers of **4**.

Conformations	*G* (hartree)	*ΔG* (kcal/mol)	Boltzmann distributions (%)
11*S*,12*R*-**4-1**	-1085.577298	0	88.7
11*S*,12*R*-**4-2**	-1085.574709	1.624621837	5.7
11*S*,12*R*-**4-3**	-1085.574684	1.640309572	5.6

Table S10. The coordinates of the optimized conformers of **4**.

	11*S*,12*R*-**4-1**			11*S*,12*R*-**4-2**			11*S*,12*R*-**4-3**		
		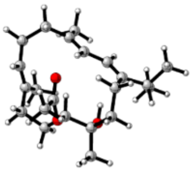			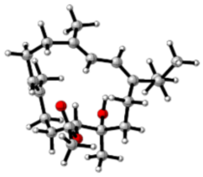			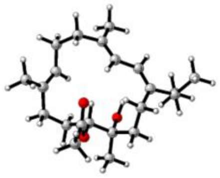	
C	3.08762	1.36628	0.00373	2.67461	1.40715	0.10277	-2.23112	1.839	-0.94897
C	2.94987	2.80018	-0.41803	2.73358	2.65431	-0.73134	-2.12611	3.23628	-0.40061
C	1.75611	3.02719	-1.38231	1.64452	2.65504	-1.83774	-1.13929	3.33682	0.79154
C	0.4062	2.89399	-0.72183	0.24664	2.6501	-1.27465	0.28152	2.99205	0.42033
C	-0.3241	1.775	-0.89907	-0.47354	1.51108	-1.28611	0.79097	1.78848	0.75038
C	-1.65661	1.52153	-0.3778	-1.78737	1.31107	-0.70074	2.11953	1.29424	0.43054
C	-2.38012	0.38726	-0.50946	-2.42789	0.13641	-0.51926	2.58249	0.03656	0.59434
C	-1.8603	-0.85725	-1.19588	-1.86002	-1.20916	-0.9072	1.75677	-1.10467	1.13954
C	-1.68802	-2.08996	-0.28037	-1.52957	-2.13365	0.28529	1.44254	-2.21093	0.10853
C	-0.75702	-1.9543	0.94068	-0.3819	-1.7029	1.22417	0.53011	-1.84775	-1.08143
C	0.68065	-1.53555	0.54368	0.93356	-1.46702	0.43825	-0.84542	-1.31152	-0.61156
C	1.64814	-1.46123	1.71762	2.22101	-1.30953	1.24922	-1.84789	-1.05888	-1.732
C	2.69588	0.80567	1.15245	2.35537	1.28699	1.39518	-3.14596	0.91941	-0.62564
C	2.92583	-0.66716	1.40709	2.3084	-0.04948	2.11317	-3.17164	-0.46763	-1.24029
C	2.00685	1.554	2.26444	2.01563	2.44424	2.29826	-4.25427	1.14637	0.3683
C	-0.01772	4.07274	0.11096	-0.20367	3.9382	-0.64151	1.01293	4.04735	-0.36319
C	-3.8245	0.30695	-0.03133	-3.83321	0.10373	0.07425	4.03109	-0.30696	0.26141
C	-4.13863	1.12981	1.22208	-3.95285	0.85609	1.40641	4.4578	0.12903	-1.14651
C	-4.7744	0.69349	-1.18095	-4.86104	0.61747	-0.94789	4.97688	0.26422	1.33118
O	1.20868	-2.52554	-0.38943	1.11926	-2.63332	-0.42136	-1.42862	-2.30058	0.28892
C	-0.76542	-3.26551	1.72054	-0.21774	-2.75201	2.31809	0.38556	-3.07251	-1.98039
O	-1.25319	-0.95533	1.84942	-0.71874	-0.48045	1.90375	1.12674	-0.82907	-1.9028
C	1.54489	-2.16059	-1.63869	1.50249	-2.43112	-1.69349	-1.78287	-1.92711	1.53147
O	1.42179	-1.0415	-2.08393	1.70541	-1.33827	-2.17584	-1.60984	-0.81873	1.98858
C	2.08118	-3.3384	-2.40761	1.61416	-3.73573	-2.43458	-2.43283	-3.06845	2.26533
H	3.52707	0.71106	-0.74723	2.87002	0.50108	-0.46536	-1.4444	1.56063	-1.64519
H	3.86259	3.1068	-0.94107	3.70909	2.71428	-1.22918	-3.09814	3.60697	-0.06796
H	2.85553	3.46596	0.44286	2.644	3.5582	-0.12637	-1.80078	3.91524	-1.19569
H	1.83811	2.31849	-2.21105	1.79184	1.77632	-2.4716	-1.49064	2.66995	1.58341
H	1.85072	4.03192	-1.81236	1.79449	3.53896	-2.46931	-1.17878	4.35944	1.18697
H	0.12461	1.00068	-1.5115	-0.00611	0.65172	-1.75617	0.13504	1.12107	1.30098
H	-2.12968	2.3486	0.14089	-2.30103	2.20592	-0.36116	2.81218	2.01977	0.01417
H	-2.57775	-1.14868	-1.97263	-2.61484	-1.73052	-1.50874	0.83613	-0.75049	1.59987
H	-0.92557	-0.65688	-1.71432	-0.98893	-1.10263	-1.55305	2.33176	-1.5808	1.94358
H	-2.66364	-2.39695	0.10582	-1.29955	-3.12665	-0.10589	0.99169	-3.05436	0.6372

H	-1.33354	-2.92143	-0.89581	-2.41882	-2.24665	0.91169	2.37856	-2.58417	-0.31636
H	0.64506	-0.57719	0.02836	0.80087	-0.59969	-0.20166	-0.69556	-0.39731	-0.04378
H	1.12254	-1.0149	2.56266	3.05205	-1.33065	0.53846	-2.06686	-2.00307	-2.23639
H	1.92022	-2.47707	2.01498	2.35416	-2.18994	1.88217	-1.3711	-0.41479	-2.47266
H	3.44133	-1.11546	0.55237	3.20355	-0.12872	2.74534	-3.61129	-1.16056	-0.51438
H	3.60055	-0.77648	2.26637	1.46391	-0.03245	2.80507	-3.88277	-0.46109	-2.07674
H	2.40553	1.26056	3.24139	2.20004	3.41897	1.84858	-5.22299	0.85603	-0.05393
H	2.10386	2.63497	2.17133	2.59213	2.39096	3.22865	-4.09375	0.51293	1.24783
H	0.93582	1.32726	2.28024	0.95905	2.40107	2.58648	-4.33097	2.1776	0.71085
H	0.76859	4.35132	0.8197	0.01285	4.7868	-1.29849	1.04028	4.98935	0.19584
H	-0.18373	4.95222	-0.5223	0.33664	4.12334	0.29327	2.03712	3.77523	-0.61313
H	-0.92575	3.89723	0.68608	-1.26754	3.95772	-0.40807	0.49406	4.26399	-1.30338
H	-4.02782	-0.74026	0.21624	-4.07713	-0.94469	0.27519	4.12374	-1.39735	0.30085
H	-5.16619	0.94247	1.54465	-4.95821	0.74234	1.82174	5.47704	-0.20596	-1.35872
H	-3.47503	0.86614	2.0491	-3.23633	0.47266	2.13568	3.79424	-0.29524	-1.90287
H	-4.0487	2.20434	1.0431	-3.76877	1.92671	1.28302	4.44165	1.2168	-1.25509
H	-5.81751	0.53456	-0.89245	-4.69599	1.67661	-1.16445	4.96001	1.3576	1.31872
H	-4.58388	0.10326	-2.08063	-4.79132	0.06908	-1.89122	6.00646	-0.06127	1.15475
H	-4.64944	1.7489	-1.43935	-5.88007	0.50445	-0.5659	4.68661	-0.06291	2.33326
H	-0.2312	-3.16014	2.66542	-1.18223	-2.95417	2.78801	-0.09568	-2.80769	-2.92236
H	-1.79336	-3.55685	1.94549	0.46661	-2.40402	3.09278	1.37043	-3.48347	-2.21099
H	-0.29619	-4.0629	1.14234	0.16968	-3.68539	1.90547	-0.20807	-3.84639	-1.49085
H	-1.38847	-0.13644	1.3476	-0.9917	0.17229	1.2417	1.42922	-0.11034	-1.32736
H	1.29158	-4.08277	-2.53555	0.61312	-4.14395	-2.59707	-1.8326	-3.97479	2.17372
H	2.43444	-3.01322	-3.3836	2.09541	-3.57557	-3.39689	-2.56326	-2.80832	3.31343
H	2.89282	-3.815	-1.85526	2.1745	-4.46519	-1.84848	-3.40963	-3.27571	1.82106

Table S11. Conformational analysis of the optimized isomers of **5.**

Conformations	*G* (hartree)	*ΔG* (kcal/mol)	Boltzmann distributions (%)
1*S*,4*R*,8*R*,11*S*,12*R*-**5-1**	-1083.371222	0	19.5
1*S*,4*R*,8*R*,11*S*,12*R*-**5-2**	-1083.371053	0.106049	16.3
1*S*,4*R*,8*R*,11*S*,12*R*-**5-3**	-1083.371031	0.119854	15.9
1*S*,4*R*,8*R*,11*S*,12*R*-**5-4**	-1083.370921	0.18888	14.2
1*S*,4*R*,8*R*,11*S*,12*R*-**5-5**	-1083.370709	0.321912	11.3
1*S*,4*R*,8*R*,11*S*,12*R*-**5-6**	-1083.370464	0.475652	8.7
1*S*,4*R*,8*R*,11*S*,12*R*-**5-7**	-1083.370057	0.731048	5.7
1*S*,4*R*,8*R*,11*S*,12*R*-**5-8**	-1083.369897	0.83145	4.8
1*S*,4*R*,8*R*,11*S*,12*R*-**5-9**	-1083.36964	0.99272	3.6

Table S12. The coordinates of the optimized conformers of **5**.

	1*S*,4*R*,8*R*,11*S*,12*R*-**5-1**			1*S*,4*R*,8*R*,11*S*,12*R*-**5-2**			1*S*,4*R*,8*R*,11*S*,12*R*-**5-3**			1*S*,4*R*,8*R*,11*S*,12*R*-**5-4**			1*S*,4*R*,8*R*,11*S*,12*R*-**5-5**			1*S*,4*R*,8*R*,11*S*,12*R*-**5-6**			1*S*,4*R*,8*R*,11*S*,12*R*-**5-7**			1*S*,4*R*,8*R*,11*S*,12*R*-**5-8**			1*S*,4*R*,8*R*,11*S*,12*R*-**5-9**		
	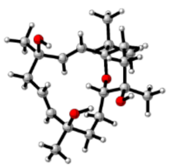			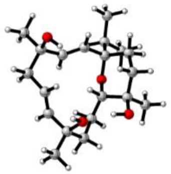			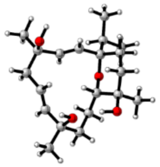			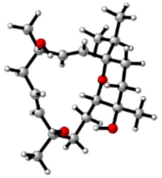			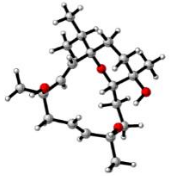			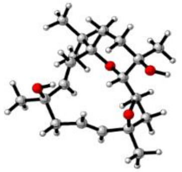			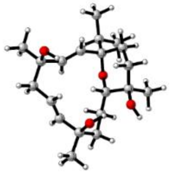			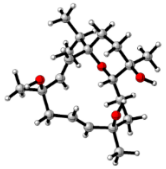			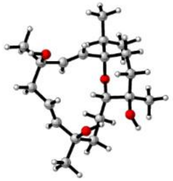		
C	0.15362	3.13679	0.22653	-0.92272	2.90927	-0.37147	0.32321	3.15953	0.1848	0.17824	3.14599	0.20131	-0.97468	2.89176	-0.35882	-0.91083	2.89914	-0.40246	0.17516	3.13931	0.22306	-0.98127	2.88129	-0.38468	0.2429	3.13918	0.21266
C	0.80713	1.85547	-0.23108	-1.21525	1.51661	0.13065	0.94532	1.85284	-0.24505	0.82112	1.85358	-0.23865	-1.25113	1.49712	0.13176	-1.20694	1.51542	0.12311	0.81687	1.85209	-0.2348	-1.24764	1.49022	0.1204	0.87457	1.84889	-0.23001
C	1.5132	1.01985	0.52279	-1.74883	0.5213	-0.5702	1.48245	0.9339	0.54968	1.52043	1.02014	0.52414	-1.73427	0.48774	-0.58379	-1.74987	0.5147	-0.56228	1.51898	1.01297	0.51871	-1.73851	0.47445	-0.58039	1.50661	0.97279	0.54186
C	2.03648	-0.31326	0.00323	-1.9043	-0.89072	-0.02866	1.98824	-0.40828	0.0289	2.03167	-0.32343	0.01771	-1.88243	-0.9226	-0.03487	-1.91256	-0.88988	-0.00576	2.0334	-0.32458	0.00228	-1.88355	-0.92994	-0.01746	2.01646	-0.36238	0.01315
C	2.28987	-1.29409	1.16216	-1.85599	-1.93283	-1.16651	2.18662	-1.40856	1.18275	2.26461	-1.30031	1.1852	-1.81656	-1.97079	-1.16629	-1.88122	-1.93916	-1.13535	2.27674	-1.3042	1.164	-1.82536	-1.98649	-1.13958	2.24497	-1.35789	1.1641
C	0.98247	-1.80878	1.77151	-0.44128	-2.16897	-1.70385	0.85796	-1.91997	1.75031	0.95224	-1.8304	1.77235	-0.39733	-2.19233	-1.69755	-0.47834	-2.1511	-1.70837	0.96881	-1.81294	1.77636	-0.41484	-2.18409	-1.69895	0.92984	-1.85976	1.7668
C	0.019	-2.35231	0.70646	0.58803	-2.42274	-0.5876	-0.10912	-2.41226	0.65736	-0.00601	-2.37404	0.69612	0.63214	-2.42342	-0.57658	0.56524	-2.39864	-0.61575	0.00316	-2.35022	0.71551	0.62312	-2.40204	-0.59493	-0.04069	-2.36951	0.69703
C	-0.19438	-1.21529	-0.30891	0.47258	-1.20792	0.35738	-0.25748	-1.233	-0.3282	-0.20001	-1.22004	-0.31038	0.49444	-1.20477	0.36101	0.46792	-1.20513	0.35507	-0.20059	-1.21679	-0.30961	0.49746	-1.19763	0.35954	-0.22525	-1.21991	-0.31418
C	-1.15668	-1.44543	-1.46882	1.43255	-1.10204	1.53571	-1.23166	-1.37081	-1.49122	-1.16085	-1.41995	-1.47602	1.45369	-1.07365	1.53737	1.43945	-1.13246	1.52813	-1.16454	-1.44458	-1.46922	1.46561	-1.08694	1.53217	-1.19605	-1.41598	-1.47368
C	3.29853	-0.0799	-0.87074	-3.22057	-1.05367	0.78337	3.28034	-0.2017	-0.80677	3.30693	-0.10423	-0.84238	-3.20157	-1.09929	0.76927	-3.21758	-1.03932	0.82498	3.2995	-0.10114	-0.86861	-3.19278	-1.10156	0.80242	3.28928	-0.14296	-0.8481
C	4.43448	0.57404	-0.07843	-3.26246	-0.18299	2.04135	4.41987	0.3819	0.03386	4.44238	0.53575	-0.038	-3.2657	-0.22809	2.02595	-3.23744	-0.15582	2.07454	4.4375	0.54713	-0.07458	-3.24409	-0.21662	2.05004	4.43195	0.47422	-0.0363
C	3.7889	-1.34444	-1.58326	-4.46596	-0.8058	-0.07457	3.73861	-1.46444	-1.54342	3.7899	-1.37418	-1.5502	-4.44355	-0.86825	-0.0983	-4.47243	-0.79219	-0.01926	3.78389	-1.37043	-1.5768	-4.44397	-0.881	-0.05459	3.75959	-1.40621	-1.57643
O	1.06943	-0.89193	-0.90253	-0.85255	-1.17938	0.91692	1.02595	-0.94526	-0.90698	1.06987	-0.89076	-0.89756	-0.83194	-1.19747	0.91843	-0.84885	-1.18547	0.92762	1.06506	-0.8985	-0.90532	-0.82176	-1.20231	0.92693	1.0465	-0.91965	-0.90571
C	0.51272	-3.647	0.07058	0.34842	-3.74796	0.1251	0.3888	-3.68513	-0.01545	0.52803	-3.64349	0.04447	0.41146	-3.74813	0.14304	0.39389	-3.74825	0.06813	0.48517	-3.65144	0.08909	0.4674	-3.74519	0.1054	0.42451	-3.66914	0.05455
O	-1.27425	-2.58319	1.31327	1.89803	-2.51272	-1.16092	-1.36827	-2.74599	1.25554	-1.2501	-2.73599	1.30911	1.94478	-2.49654	-1.14644	1.85012	-2.35137	-1.29333	-1.2516	-2.57732	1.41376	1.91221	-2.34632	-1.26405	-1.29897	-2.58974	1.39148
C	-1.32558	3.13947	-0.25689	0.52244	3.29533	0.0608	-1.16353	3.17721	-0.24523	-1.3096	3.14026	-0.25765	0.46309	3.29282	0.07782	0.52304	3.30218	0.04816	-1.3063	3.15195	-0.25306	0.44943	3.30279	0.0546	-1.23576	3.16034	-0.26678
C	-2.04889	1.88125	0.12252	1.50946	2.21164	-0.25637	-1.88214	1.92159	0.14975	-2.01046	1.87133	0.12695	1.45424	2.21858	-0.25373	1.53447	2.23848	-0.26128	-2.03581	1.89806	0.12872	1.46752	2.25143	-0.26949	-1.97079	1.91537	0.12867
C	-2.60882	1.02417	-0.72616	2.29471	1.59912	0.62527	-2.58544	1.1478	-0.67191	-2.63246	1.0437	-0.7077	2.27751	1.63745	0.6144	2.2605	1.5836	0.64026	-2.59577	1.04209	-0.72085	2.24175	1.63746	0.62052	-2.59019	1.08858	-0.70815
C	-3.24717	-0.29235	-0.32552	3.21013	0.43373	0.32151	-3.24899	-0.15865	-0.2961	-3.24849	-0.28421	-0.32421	3.20348	0.48151	0.30895	3.20549	0.44033	0.32402	-3.24382	-0.27026	-0.32209	3.19768	0.50385	0.30387	-3.24843	-0.21739	-0.30565
C	-2.66222	-1.46818	-1.15651	2.90359	-0.78895	1.22077	-2.73053	-1.33663	-1.15584	-2.66251	-1.45182	-1.15478	2.91924	-0.73997	1.21684	2.90964	-0.78785	1.22927	-2.67256	-1.4445	-1.16367	2.9285	-0.71724	1.22696	-2.70278	-1.39312	-1.16213
C	0.8917	4.35611	-0.32292	-1.92483	3.91967	0.18426	1.06888	4.34287	-0.44312	0.90583	4.35297	-0.38774	-1.99745	3.88247	0.21023	-1.93057	3.91355	0.11253	0.91939	4.35176	-0.33326	-2.01781	3.86609	0.1715	1.00407	4.34004	-0.36256
O	0.20256	3.27244	1.65479	-1.04617	2.99276	-1.79961	0.30753	3.30046	1.61288	0.2494	3.31504	1.62506	-1.05838	2.87428	-1.79169	-1.00941	2.95027	-1.8341	0.23209	3.27819	1.65062	-1.06378	2.84856	-1.81727	0.28862	3.17898	1.6464
C	-4.76133	-0.21619	-0.56769	4.67524	0.84699	0.511	-4.77046	-0.04363	-0.45688	-4.76954	-0.23775	-0.5169	4.66526	0.91369	0.48432	4.65104	0.89664	0.56679	-4.75783	-0.18012	-0.5584	4.64044	0.98054	0.52473	-4.76456	-0.10901	-0.52089
O	-3.07627	-0.51673	1.0806	3.03667	-0.01776	-1.04167	-2.96308	-0.51142	1.07744	-2.97319	-0.60211	1.05995	3.02668	0.0199	-1.05017	3.12744	0.09674	-1.06522	-3.07496	-0.5059	1.08193	3.10606	0.14431	-1.08038	-3.06231	-0.46194	1.09476
H	0.59997	1.58032	-1.26239	-0.87822	1.31908	1.14371	0.87412	1.64854	-1.31132	0.6079	1.56499	-1.26501	-0.95412	1.31855	1.16048	-0.86973	1.33202	1.13874	0.60466	1.57687	-1.26508	-0.94238	1.32251	1.14851	0.7218	1.61345	-1.28041
H	1.72073	1.26905	1.55943	-2.0849	0.69671	-1.58844	1.52712	1.10352	1.62153	1.73815	1.27981	1.55624	-2.00986	0.65312	-1.62013	-2.08198	0.6799	-1.5834	1.73111	1.26325	1.55415	-2.0224	0.62923	-1.61609	1.63944	1.18714	1.59689
H	2.88858	-0.80579	1.9349	-2.51132	-1.62036	-1.98316	2.77366	-0.93851	1.97655	2.84892	-0.80516	1.96546	-2.47255	-1.67087	-1.98711	-2.55855	-1.64289	-1.93917	2.87851	-0.81798	1.93576	-2.49922	-1.70519	-1.95168	2.84882	-0.88653	1.94346
H	2.87648	-2.13866	0.79734	-2.2616	-2.87397	-0.78405	2.77238	-2.25617	0.82287	2.86513	-2.1407	0.83297	-2.21172	-2.915	-0.7802	-2.26185	-2.88409	-0.73683	2.85957	-2.15279	0.80172	-2.19629	-2.93427	-0.73844	2.82012	-2.20866	0.79438
H	1.19613	-2.58729	2.51237	-0.43767	-3.01182	-2.40184	1.03661	-2.72633	2.46827	1.15407	-2.61491	2.50807	-0.38015	-3.04028	-2.3893	-0.47661	-2.98801	-2.41237	1.17029	-2.59392	2.51488	-0.39509	-3.02955	-2.39253	1.12057	-2.65375	2.49437
H	0.46618	-0.99895	2.29746	-0.103	-1.291	-2.26661	0.34897	-1.11786	2.29723	0.4264	-1.02766	2.30241	-0.06903	-1.31405	-2.26547	-0.16235	-1.26394	-2.26751	0.45677	-1.00084	2.30334	-0.10741	-1.29913	-2.26605	0.42661	-1.04929	2.30439
H	-0.54558	-0.35265	0.25847	0.6043	-0.3179	-0.25862	-0.56601	-0.36839	0.26022	-0.54616	-0.35919	0.26305	0.60921	-0.3159	-0.25968	0.59846	-0.30555	-0.24751	-0.54679	-0.34906	0.25364	0.60786	-0.30248	-0.25345	-0.55342	-0.35139	0.25846
H	-0.89113	-2.36498	-1.99902	1.39255	-2.01536	2.13731	-1.0206	-2.28378	-2.05672	-0.90211	-2.33026	-2.0255	1.4293	-1.98219	2.14726	1.40419	-2.06191	2.10506	-0.90933	-2.36912	-1.9964	1.44928	-2.00773	2.12392	-0.95893	-2.33789	-2.01398
H	-0.95764	-0.62913	-2.16862	1.03164	-0.30635	2.17036	-1.00274	-0.53935	-2.16396	-0.96524	-0.58988	-2.161	1.04138	-0.27812	2.16488	1.0329	-0.35969	2.18598	-0.95643	-0.6348	-2.17349	1.04355	-0.31133	2.17676	-0.97672	-0.59996	-2.16719
H	2.97223	0.6285	-1.64102	-3.22561	-2.10222	1.10511	3.00735	0.54032	-1.56588	2.99792	0.60793	-1.61648	-3.19662	-2.14722	1.09331	-3.22398	-2.08477	1.15671	2.98002	0.60761	-1.64146	-3.18269	-2.14607	1.13725	2.98572	0.58397	-1.61032
H	4.82424	-0.09771	0.69164	-2.37405	-0.32995	2.65549	5.27304	0.62793	-0.6037	5.28393	0.77145	-0.69455	-2.37824	-0.35871	2.64523	-2.34063	-0.29934	2.67728	5.27168	0.78725	-0.73877	-4.1121	-0.48539	2.65858	5.27057	0.72213	-0.69211

H	5.26478	0.82191	-0.74463	-3.33341	0.87813	1.79044	4.11624	1.29447	0.55195	4.12749	1.46337	0.44521	-3.35459	0.83121	1.77354	-3.30871	0.90277	1.81355	4.12046	1.47326	0.4099	-2.34926	-0.3378	2.66065	4.12169	1.39097	0.46997
H	4.11241	1.49652	0.40968	-4.13965	-0.44095	2.64139	4.76904	-0.33238	0.78474	4.81341	-0.14009	0.73769	-4.14148	-0.5013	2.62139	-4.10647	-0.4046	2.68999	4.81994	-0.12515	0.69869	-3.33874	0.83943	1.78661	4.80517	-0.22058	0.7214
H	4.22604	-2.06492	-0.88651	-4.48564	0.22154	-0.44984	2.92427	-1.90072	-2.1212	4.20701	-2.10194	-0.84877	-4.46869	0.1565	-0.48033	-4.48846	0.23024	-0.40772	2.9705	-1.85795	-2.11382	-4.47284	0.13876	-0.4493	4.1716	-2.14823	-0.88694
H	4.56687	-1.08413	-2.30652	-5.369	-0.95435	0.52317	4.11682	-2.22627	-0.85622	4.58136	-1.12361	-2.26232	-5.34956	-1.02093	0.49402	-5.36814	-0.92529	0.59281	4.214	-2.09222	-0.87703	-5.34321	-1.02571	0.54977	4.55206	-1.15207	-2.28612
H	2.97692	-1.83611	-2.11853	-4.52578	-1.47939	-0.9314	4.5531	-1.21831	-2.23075	2.97816	-1.8533	-2.09697	-4.49148	-1.54756	-0.95136	-4.55011	-1.47657	-0.86606	4.56589	-1.11688	-2.29811	-4.50206	-1.57129	-0.89816	2.94314	-1.87026	-2.12929
H	0.73191	-4.39107	0.84178	-0.60917	-3.75662	0.64446	-0.34557	-4.04618	-0.73823	1.45877	-3.46028	-0.49125	-0.54978	-3.77099	0.65509	1.22041	-3.95263	0.75489	-0.27133	-4.07265	-0.57906	0.45642	-4.55728	-0.62536	0.62997	-4.4204	0.82081
H	1.41993	-3.48156	-0.51091	1.13781	-3.93369	0.85604	0.53467	-4.46723	0.73429	-0.20208	-4.04522	-0.66075	1.19833	-3.91483	0.88139	0.3603	-4.55013	-0.67307	0.7047	-4.389	0.86467	-0.45872	-3.77839	0.67794	1.33048	-3.51133	-0.52946
H	-0.24899	-4.06078	-0.59192	0.36089	-4.56716	-0.59863	1.33191	-3.52212	-0.5358	0.70985	-4.40349	0.80891	0.44468	-4.57145	-0.5754	-0.52648	-3.77285	0.65044	1.38661	-3.48856	-0.50056	1.28989	-3.92557	0.80381	-0.33553	-4.07006	-0.62215
H	-1.16121	-3.10643	2.11607	2.24001	-1.61554	-1.31915	-1.87897	-1.92898	1.39282	-1.79743	-1.93875	1.41712	2.26913	-1.59417	-1.31291	2.45884	-2.95926	-0.85815	-1.69233	-3.34394	1.02936	2.52931	-2.93012	-0.8081	-1.75502	-3.33699	0.98746
H	-1.80074	4.01963	0.19092	0.7714	4.22496	-0.46237	-1.618	4.05496	0.22848	-1.78568	4.01095	0.20663	0.71256	4.23277	-0.43111	0.7632	4.24029	-0.46433	-1.77385	4.03473	0.19738	0.68211	4.24916	-0.45105	-1.70595	4.05174	0.16979
H	-1.34607	3.27155	-1.34296	0.53353	3.50743	1.13387	-1.22147	3.3239	-1.32685	-1.35493	3.27877	-1.34171	0.47247	3.50016	1.15199	0.51314	3.5073	1.12305	-1.33114	3.28441	-1.33896	0.44657	3.5099	1.12932	-1.26022	3.29064	-1.35309
H	-2.07008	1.61616	1.17699	1.51718	1.86104	-1.28615	-1.74317	1.61836	1.18329	-1.93591	1.58535	1.17381	1.40822	1.85568	-1.2765	1.63848	1.93399	-1.30009	-2.06123	1.6362	1.18397	1.5138	1.92667	-1.30419	-1.9283	1.64657	1.17918
H	-2.61987	1.24516	-1.79307	2.29605	1.92389	1.66452	-2.7075	1.43651	-1.71486	-2.71301	1.29913	-1.763	2.30581	1.98283	1.64679	2.19225	1.85694	1.69283	-2.60145	1.26191	-1.78801	2.20595	1.93657	1.66799	-2.63875	1.32581	-1.77076
H	-2.93716	-2.3926	-0.6432	3.38864	-1.64845	0.75558	-3.0321	-2.25358	-0.647	-2.92547	-2.37176	-0.63022	3.41646	-1.59549	0.75694	3.44073	-1.63975	0.79524	-2.98136	-2.37083	-0.67115	3.47608	-1.56499	0.8052	-3.02645	-2.31999	-0.67991
H	-3.18306	-1.48783	-2.11878	3.4104	-0.62463	2.176	-3.27577	-1.3128	-2.10375	-3.19722	-1.47336	-2.10865	3.42615	-0.56086	2.16944	3.3898	-0.61021	2.19633	-3.18723	-1.4433	-2.12927	3.40631	-0.5155	2.19053	-3.22149	-1.37111	-2.12542
H	0.3759	5.27433	-0.03047	-2.92728	3.68388	-0.17773	0.5738	5.28134	-0.18213	1.92885	4.38957	-0.00859	-1.75295	4.90654	-0.09095	-1.66995	4.91923	-0.22752	0.41158	5.27453	-0.04116	-1.78374	4.88964	-0.13957	2.02209	4.35648	0.0315
H	1.90859	4.38404	0.07337	-1.93517	3.89603	1.27559	2.09881	4.37962	-0.07683	0.94064	4.29199	-1.47706	-2.99459	3.63753	-0.1606	-2.92467	3.66293	-0.26256	1.93825	4.37345	0.05831	-3.01088	3.60637	-0.20039	1.05429	4.29344	-1.45294
H	0.94447	4.31972	-1.4127	-1.66329	4.93066	-0.13892	1.10383	4.25742	-1.53184	0.39453	5.27752	-0.10753	-2.01597	3.84907	1.30196	-1.96134	3.91336	1.20379	0.96694	4.31184	-1.42317	-2.04006	3.84406	1.26352	0.508	5.27671	-0.08815
H	-0.17308	2.47359	2.04527	-0.54204	2.26775	-2.1894	1.22105	3.31459	1.92387	-0.08385	2.5118	2.04373	-0.99341	3.78497	-2.10323	-0.46713	2.23848	-2.19635	-0.14654	2.48284	2.04529	-0.99036	3.75547	-2.13779	-0.06409	4.03149	1.92824
H	-4.98886	0.07981	-1.59492	5.33229	-0.01152	0.35229	-5.24513	-1.00466	-0.24451	-5.02621	0.03935	-1.54172	5.33145	0.06272	0.32297	4.91531	1.67194	-0.1555	-5.22705	-1.14982	-0.37049	4.77628	1.39863	1.52532	-5.17971	0.62673	0.17133
H	-5.22271	-1.18875	-0.37492	4.9463	1.63187	-0.20077	-5.17178	0.70457	0.2324	-5.20676	-1.21545	-0.30038	4.92011	1.69839	-0.2335	4.77914	1.30333	1.57298	-5.19119	0.55324	0.12506	5.33698	0.14712	0.39743	-5.00355	0.20262	-1.54078
H	-5.20388	0.51785	0.10932	4.84622	1.23521	1.51745	-5.03632	0.25929	-1.47214	-5.21756	0.50237	0.15205	4.84067	1.30804	1.48776	5.33808	0.05535	0.4403	-4.98568	0.12503	-1.58269	4.88582	1.75204	-0.20827	-5.24293	-1.07365	-0.32967
H	-2.43229	-1.23314	1.22399	3.3399	0.66545	-1.65116	-3.31449	0.16842	1.66475	-3.41938	0.03202	1.63374	3.25044	0.7294	-1.66399	2.6302	-0.73338	-1.1785	-2.40128	-1.19427	1.22793	2.64466	-0.70804	-1.17698	-2.41338	-1.17588	1.22798
